# Mechanism of *Radix Rhei Et Rhizome* Intervention in Cerebral Infarction: A Research Based on Chemoinformatics and Systematic Pharmacology

**DOI:** 10.1155/2021/6789835

**Published:** 2021-09-06

**Authors:** Wang Xiang, Zhiyong Long, Jinsong Zeng, Xiaofei Zhu, Mengxia Yuan, Jiamin Wu, Yonghe Wu, Liang Liu

**Affiliations:** ^1^The Affiliated Hospital of Guilin Medical University, Guilin, Guangxi Province, China; ^2^Shantou University Medical College, Shantou University, Shantou, Guangdong, China; ^3^The First Affiliated Hospital of Hunan University of Chinese Medicine, Changsha, Hunan Province, China; ^4^Hunan University of Chinese Medicine, Changsha, Hunan Province, China; ^5^Shanghai University of Traditional Chinese Medicine, Shanghai, China

## Abstract

**Objective:**

To explore the therapeutic targets, network modules, and coexpressed genes of *Radix Rhei Et Rhizome* intervention in cerebral infarction (CI), and to predict significant biological processes and pathways through network pharmacology. To explore the differential proteins of *Radix Rhei Et Rhizome* intervention in CI, conduct bioinformatics verification, and initially explain the possible therapeutic mechanism of *Radix Rhei Et Rhizome* intervention in CI through proteomics.

**Methods:**

The TCM database was used to predict the potential compounds of Radix Rhei Et Rhizome, and the PharmMapper was used to predict its potential targets. GeneCards and OMIM were used to search for CI-related genes. Cytoscape was used to construct a protein-protein interaction (PPI) network and to screen out core genes and detection network modules. Then, DAVID and Metascape were used for enrichment analysis. After that, in-depth analysis of the proteomics data was carried out to further explore the mechanism of *Radix Rhei Et Rhizome* intervention in CI.

**Results:**

(1) A total of 14 *Radix Rhei Et Rhizome* potential components and 425 potential targets were obtained. The core components include sennoside A, palmidin A, emodin, toralactone, and so on. The potential targets were combined with 297 CI genes to construct a PPI network. The targets shared by *Radix Rhei Et Rhizome* and CI include ALB, AKT1, MMP9, IGF1, CASP3, etc. The biological processes that *Radix Rhei Et Rhizome* may treat CI include platelet degranulation, cell migration, fibrinolysis, platelet activation, hypoxia, angiogenesis, endothelial cell apoptosis, coagulation, and neuronal apoptosis. The signaling pathways include Ras, PI3K-Akt, TNF, FoxO, HIF-1, and Rap1 signaling pathways. (2) Proteomics shows that the top 20 proteins in the differential protein PPI network were Syp, Syn1, Mbp, Gap43, Aif1, Camk2a, Syt1, Calm1, Calb1, Nsf, Nefl, Hspa5, Nefh, Ncam1, Dcx, Unc13a, Mapk1, Syt2, Dnm1, and Cltc. Differential protein enrichment results show that these proteins may be related to synaptic vesicle cycle, vesicle-mediated transport in synapse, presynaptic endocytosis, synaptic vesicle endocytosis, axon guidance, calcium signaling pathway, and so on.

**Conclusion:**

This study combined network pharmacology and proteomics to explore the main material basis of *Radix Rhei Et Rhizome* for the treatment of CI such as sennoside A, palmidin A, emodin, and toralactone. The mechanism may be related to the regulation of biological processes (such as synaptic vesicle cycle, vesicle-mediated transport in synapse, presynaptic endocytosis, and synaptic vesicle endocytosis) and signaling pathways (such as Ras, PI3K-Akt, TNF, FoxO, HIF-1, Rap1, and axon guidance).

## 1. Introduction

Cerebral infarction (CI) or ischemic stroke (IS) mainly results from blood supply disturbances in local brain tissue areas, leading to necrosis of ischemic hypoxic lesions in the brain tissue, which results in the manifestation of corresponding neurological deficits [1]. Epidemiological studies have shown that stroke has become the disease with the highest mortality rate in China [2, 3]. CI is divided into cerebral thrombosis, cerebral embolism, and lacunar infarction according to the different pathogenesis. Among them, cerebral thrombosis is the most common type of CI, accounting for about 60% [4]. Timely thrombolysis to restore blood supply after infarction is the most important measure to save the ischemic area. Although reperfusion after ischemia can restore its function, ischemia-reperfusion injury makes the irreversible damage to the brain tissue after the blood flow restored [5–7]. Cerebral ischemia-reperfusion injury (CIR) is mainly related to the formation of free radicals (oxygen and lipid free radicals), oxidative stress, energy metabolism disorders, apoptosis, excitatory amino acid toxicity, calcium overload, inflammation, and so on [6–9]. Currently, the preventive and therapeutic drugs for CIR include excitatory amino acid-regulating drugs, neurotrophic growth factors, free radical scavengers, nitric oxide synthase inhibitors, intracellular calcium overload inhibitors, and natural plant active compounds (flavonoids, saponins, polysaccharides) [8, 10–13]. Of particular importance is that natural plant active compounds are becoming potential CIR drugs.

**Figure 1:**
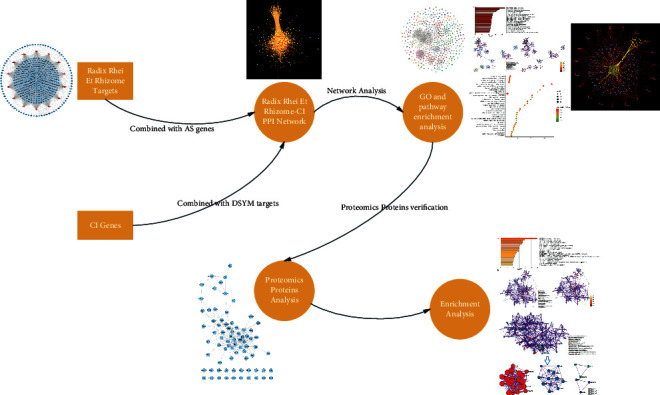
The idea and process of this research.

*Radix Rhei Et Rhizome* is an important part of the traditional Chinese medicine (TCM) formulas for the treatment of CI in the acute phase, which has a long history of medicinal use [14–18]. Modern medical research proves that rhubarb aglycones have significant protective effects on ischemic brain tissue: it can maintain the integrity of the blood-brain barrier, reduce inflammation, inhibit apoptosis, and protect nerves [15–19]. However, its specific mechanism is still unclear. Therefore, this research hopes to propose a new method to analyze the regulatory mechanism of *Radix Rhei Et Rhizome* on CI biological networks. The development of high-throughput omics and chemoinformatics has given the opportunity to analyze the mechanisms of natural plant components for disease treatment [20–24]. Therefore, based on previous research, this study will integrate proteomics and chemoinformatics strategies to further explore the molecular mechanism of *Radix Rhei Et Rhizome*'s intervention in CI and provide reference information for new drug development and its clinical application. The idea and process of this research are shown in Figure 1.

## 2. Material and Methods

### 2.1. Construction of Pharmacodynamic Molecular Database and *Radix Rhei Et Rhizome's* Compounds Prediction

All compounds of *Radix Rhei Et Rhizome* were obtained from the traditional Chinese medicine database and analysis platforms TCMSP database (http://lsp.nwu.edu.cn/) [25] and TCM@Taiwan (http://tcm.cmu.edu.tw/zh-tw/) [26]. In order to obtain potential active compounds from these compounds, this study used drug-likeness (DL), Caco-2 permeability, and oral bioavailability (OB) indicators [20–24, 27–30] and combined literature [31] to predict potential pharmacological compounds in *Radix Rhei Et Rhizome*. The standard was OB ≥ 30%, DL ≥ 0.18, and Caco-2 permeability > −0.4. After the potential compound prediction, a total of 9 *Radix Rhei Et Rhizome'*s potential compounds were obtained: (-)-catechin, aloe-emodin, beta-sitosterol, daucosterol, eupatin, mutatochrome, palmidin A, rhein, and toralactone. Meanwhile, due to the limitation of the pharmacokinetic parameter model, in order to avoid the omission of potential compounds, a large number of studies related to *Radix Rhei Et Rhizome* were searched to supplement its active compounds. Finally, according to references [32, 33], a total of 5 oral absorbable compounds with bioactivity were supplemented: chrysophanol, danthron, emodin, sennoside A, and physcion. The 3D structure of all screened compounds was saved in mol2 format.

### 2.2. Potential Targets Prediction and CI Gene Collection

In addition to screening the active components of *Radix Rhei Et Rhizome*, determining the targets of the active ingredients is also an important step to clarify the biological basis of TCM. The PharmMapper server platform (http://lilab-ecust.cn/pharmmapper/) was used to predict potential targets. After importing the “mol2” format file, the number of returned targets was set to 300, and the pharmacophore model was selected as the setting condition [34]. The PDB ID of the protein target was imported into UniProt KB (https://www.uniprot.org/uniprot/), with the species restricted to “Homo sapiens” (for potential targets) (Table S1) or “*Rattus norvegicus*” (for proteomics data) (Table S2), to obtain the official symbol of *Radix Rhei Et Rhizome* potential target.

The keyword “cerebral infarction” was entered into the GeneCards database (http://www.genecards.org/) [35] and the OMIM database (http://www.ncbi.nlm.nih.gov/omim) [36] to search for reported CI-related genes. The genes in the GeneCards database with relevance score >1 were selected. After removing duplicate genes and false positive genes, the CI gene set was obtained (Table S3).

### 2.3. Network Construction and Analysis Methods

In system pharmacology, the construction and analysis of biological network diagrams are very important for TCM pharmacological analysis. The network formed by nodes and edges (connections between nodes) is a mathematical-based and quantifiable mapping of various regulatory relationships under complex biological systems. String 11.0 (https://string-db.org/) was used to query protein-protein interaction (PPI) relationships [37]. The results were saved in TSV format, and the node1, node2, and Combinedscore information in the file was retained and imported into Cytoscape 3.7.1 software to draw the relevant network [38]. The “NetworkAnalyzer” plugin that comes with Cytoscape software was used to analyze the degree and betweenness of the network. These two parameters are often used to illustrate the importance of nodes, that is, the higher the degree and betweenness, the more important the node in the network. The clusters of networks were detected by MCODE (Cytoscape's plugin). The MCODE algorithm was originally a clustering algorithm designed to detect protein complexes in PPI networks, which can detect tightly connected regions (i.e., molecular complexes) in large-scale protein interaction networks [38]. This method can now also be used to detect clusters in other types of networks.

### 2.4. Gene Ontology (GO) Enrichment, Pathway Enrichment, and Reactome Enrichment Analysis

DAVID ver. 6.8 (https://david-d.ncifcrf.gov) was used for the GO enrichment analysis of targets and genes in clusters and for the pathway enrichment analysis of targets and genes in PPI networks [39]. The Reactome Pathway Database (https://reactome.org/) was used for reactome pathway enrichment [40].

## 3. Results and Discussion

### 3.1. Potential Compound-Potential Target Network of *Radix Rhei Et Rhizome*

**Figure 2:**
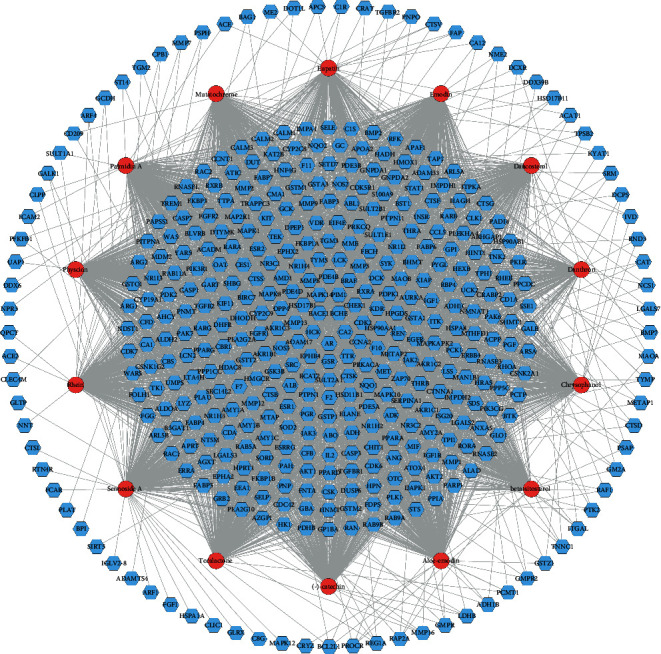
Potential Compound-Potential Target Network of *Radix Rhei Et Rhizome* (Blue hexagons stand for potential targets. Red circles stand for potential compounds.).

A total of 14 components and 425 targets were used to construct the potential compound-potential target network of *Radix Rhei Et Rhizome*. In this network, nodes near the center have a greater degree than nodes near the periphery (Figure 2).

### 3.2. *Radix Rhei Et Rhizome*-CI PPI Network Analysis

#### 3.2.1. *Radix Rhei Et Rhizome*-CI PPI Network Construction

**Figure 3:**
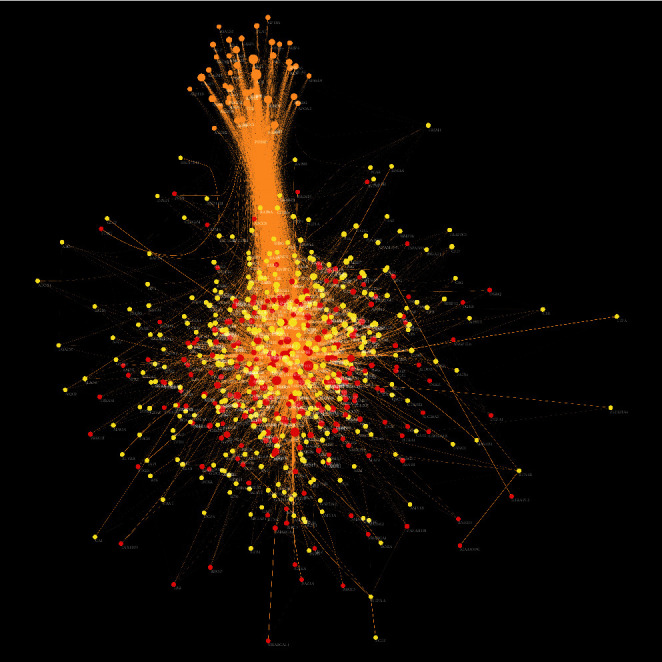
*Radix Rhei Et Rhizome*-CI PPI network (Red, yellow, and orange circles stand for CI genes, *Radix Rhei Et Rhizome* targets, and *Radix Rhei Et Rhizome*-CI targets, respectively. The larger the node size, the higher the degree of the node. The thicker the line, the greater the edge betweenness of the node.).

**Figure 4:**
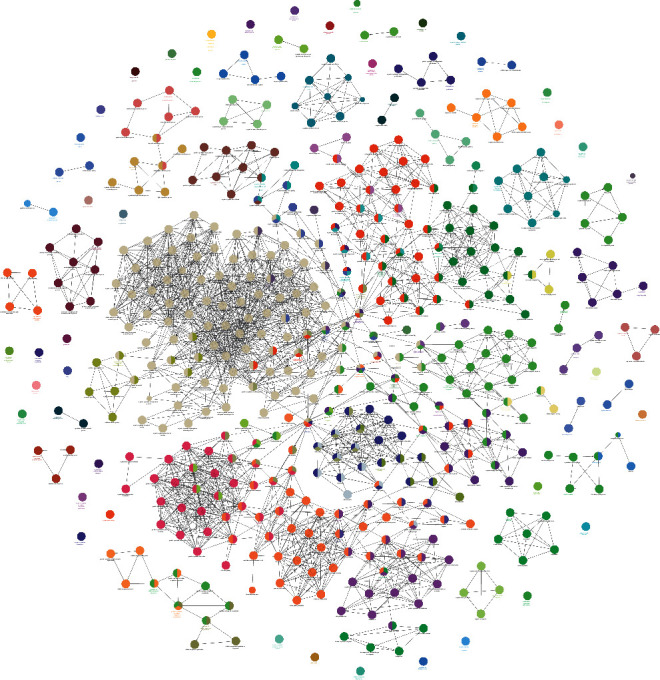
The preliminary enrichment results by ClueGO.

**Figure 5:**
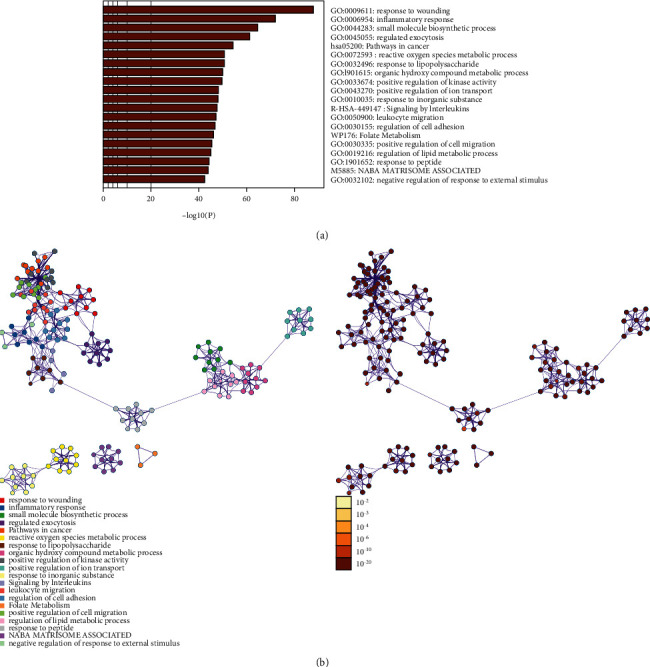
The Metascape results: (a) top biological processes, signaling pathways, and reactome pathways and (b) PPI network colored by enrichment results or *P*-values.

The *Radix Rhei Et Rhizome*-CI PPI network is composed of 645 nodes (371 potential target nodes, 231 CI gene nodes, and 43 *Radix Rhei Et Rhizome*-CI target nodes) and 14,119 edges. The following are the top 20 nodes in the network: (1) *Radix Rhei Et Rhizome* targets: EGFR (203 edges), SRC (201 edges), MAPK1 (193 edges), and MAPK8 (167 edges). (2) CI genes: INS (292 edges), IL6 (273 edges), VEGFA (244 edges), TNF (241 edges), TP53 (235 edges), EGF (210 edges), CXCL8 (178 edges), IL10 (163 edges), IL1B (160 edges), CCL2 (159 edges), and APP (157 edges). (3) *Radix Rhei Et Rhizome*-CI targets: ALB (302 edges), AKT1 (266 edges), MMP9 (191 edges), IGF1 (182 edges), and CASP3 (170 edges) (Figure 3). The preliminary enrichment results of biological processes and signaling pathways are shown in Figures 4 and 5.

In this study, a total of 14 *Radix Rhei Et Rhizome* compounds and 425 potential targets were predicted for analysis using the network pharmacological method. Although the number of predicted targets for each potential compound is different, the overlap of the target set of some compounds is large. In other words, Radix Rhei Et Rhizome's compounds have common targets probably because these compounds come from the same structural parent. For example, rhein, aloe-emodin, chrysophanol, physcion, and emodin are known as rhubarb aglycones.

In terms of the blood-brain barrier, studies have shown that emodin can maintain the integrity of the blood-brain barrier, reduce inflammation, and inhibit apoptosis [41–45]. In another study, emodin reduced blood-brain barrier permeability and reduced infarct size by inhibiting the expression of connexin 43 (Cx43) and aquaporin 4 (AQP4) in cerebral ischemia/reperfusion model rats [46]. In terms of inhibiting inflammation, emodin can inhibit transforming growth factor (TGF)-*β*, tumor necrosis factor (TNF)-*α*, interleukin (IL)-1*β*, and intercellular adhesion molecule 1 (ICAM-1), so as to protect the brain [42]. Chrysophanol inhibits the inflammatory response by reducing the expression of IL-1*β*, caspase-1, and NALP3, thereby improving neurological deficits, infarct volume, cerebral edema, and blood-brain barrier permeability in mice with ischemia-reperfusion injury. Chrysophanol can also improve the survival rate, nervous system score, and motor function of mice with middle cerebral artery occlusion by reducing the expression of TNF-*α*, IL-1*β*, and NF-*κ*B p65 [47]. In terms of inhibiting apoptosis, emodin can inhibit neuronal apoptosis [43, 44, 48]. Its specific mechanism may be that emodin can increase Bcl-2 and inhibit caspase-3 and Bax expression to reduce glutamate-induced HT22 cell apoptosis [43]. Rhein increases the expression of mature brain-derived neurotrophic factor (BDNF) and phosphorylation of Akt and cAMP response element binding protein (CREB), which improves the behavior and function of CI mice [49]. Rhein also reduced the expression of BAX, caspase-9, caspase-3, and cleaved caspase-3 and increased the expression of Bcl-2, thereby reducing the infarcted area of cerebral ischemia-reperfusion injury mice [50]. In addition, chrysophanol can inhibit NO-related neuronal cell death by attenuating nitrite and nitrate (NOx-) and 3-nitrotyrosine (3-NT) levels and reducing lysed caspase-3 protein expression [51].

In terms of oxidative stress, emodin inhibits the apoptosis of primary rat cortical neurons induced by hydrogen peroxide (H_2_O_2_) [44]. It was also found that emodin can inhibit the apoptosis of neurons after oxyglucose deprivation and reduce the damage of PC12 nerve cells by increasing the expression of activin A [45]. Chrysophanol also increases total superoxide dismutase (SOD) and manganese-dependent SOD (MnSOD) activities in cerebral ischemia-reperfusion injury models and inhibits the production of reactive oxygen species (ROS) [51]. In addition, rhein can reduce malondialdehyde (MDA) and increase the activities of SOD, catalase (CAT), and glutathione peroxidase (GSH-Px) and improve neurological function scores [50]. Chrysophanol can improve endoplasmic reticulum (ER) stress by reducing ER stress-related factors (such as glucose-regulated protein 78 (GRP78), phosphorylated eukaryotic initiation factor 2*α* (p-eIF2*α*), CCAAT-enhancer-binding protein homologous protein (CHOP), caspase-12, and NF-*κ*B/*κ*B-*α*) [52].

In terms of various compound combinations and synergies, rhubarb aglycones (aloe-emodin, rhein, emodin, chrysophanol, and physcion) can improve disorders of amino acid, energy, and lipid metabolism caused by cerebral ischemia-reperfusion injury [15]. Further research shows that rhubarb aglycones can reduce IgG content and increase type IV collagen (CoLIV) and laminin (LN) levels, thereby reducing cerebral microvascular basement membrane damage caused by thrombolysis [53]. Pharmacokinetic studies have shown that in CI model rats, the maximum plasma concentration (C max), half-life (t 1/2), and area under the curve (AUC 0-t) increased significantly, but the overall clearance (CL) value decreased significantly, indicating that rhubarb anthraquinones are more easily absorbed after coadministration [54].

#### 3.2.2. Biological Processes of *Radix Rhei Et Rhizome*-CI PPI Network

**Figure 6:**
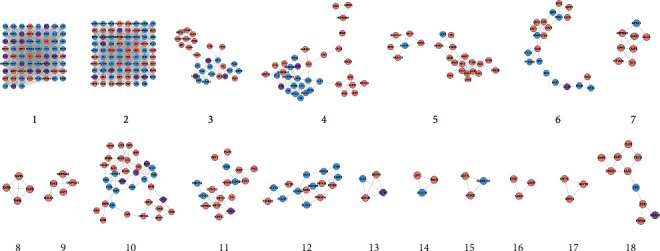
Clusters of *Radix Rhei Et Rhizome*-CI PPI network (Blue, pink, and purple circles stand for CI genes, *Radix Rhei Et Rhizome* targets, and *Radix Rhei Et Rhizome*-CI targets, respectively.).

Eighteen (18) clusters returned after analyzed by MCODE (Table 1 and Figure 6). The cluster score (complex score) is defined as the product of the complex subgraph, *C* = (*V*, *E*), density, and the number of vertices in the complex subgraph (DC × |V|). The higher the score, the denser the cluster.

**Figure 7:**
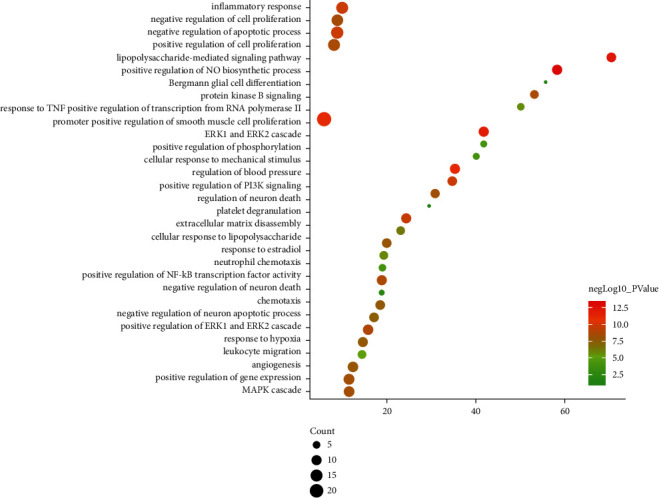
Bubble chart of biological processes (The *X*-axis is fold enrichment.).

The potential targets and CI genes in the cluster were introduced into DAVID for GO enrichment analysis. The biological process of the top 10 clusters is taken as an example (Table S4). For example, cluster 1 is related to GO:0045429, GO:0031663, GO:0048661, GO:0071260, GO:0045944, GO:0008217, GO:0006954, GO:0043066, GO:0002576, GO:0070374, and GO:0051092; cluster 2 is associated with GO:0002576, GO:0070374, GO:0043066, GO:0030335, GO:0001934, GO:0010628, GO:0042730, GO:0030168, GO:0007165, GO:0043406, and GO:0043065; and cluster 3 is involved in GO:0042632, GO:0019433, GO:0042157, GO:0017187, GO:0007584, GO:0034375, GO:0043691, GO:0019915, GO:0006465, and GO:0070328. The details of clusters and biological processes are shown in Table S4. Since the biological processes in cluster 1 are representative, the main biological processes of cluster 1 are shown as an example (Figure 7).

#### 3.2.3. Signaling Pathways of *Radix Rhei Et Rhizome*-CI PPI Network

**Figure 8:**
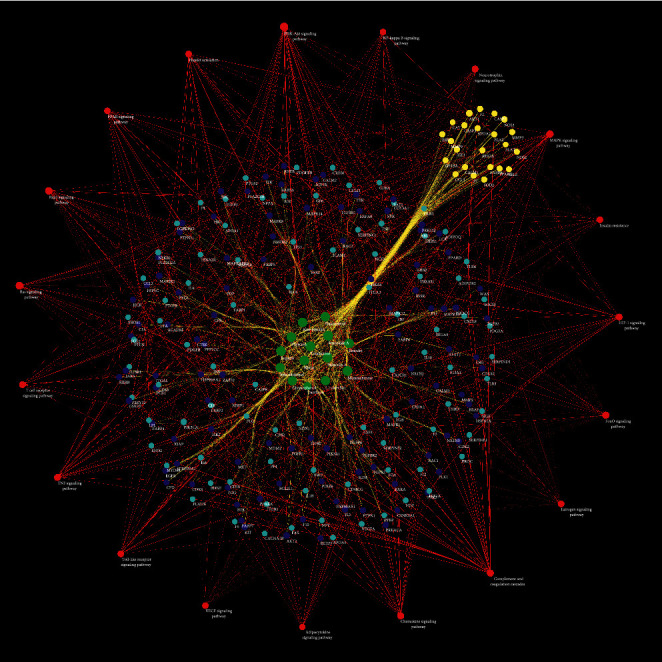
Signaling pathways of *Radix Rhei Et Rhizome*-CI PPI network (Red circles stand for signaling pathways. Dark blue circles stand for *Radix Rhei Et Rhizome* targets. Light blue circles stand for CI genes. Yellow circles stand for *Radix Rhei Et Rhizome*-CI targets. Green circles stand for *Radix Rhei Et Rhizome* compounds. The larger the node size, the higher the degree of the node. The thicker the line, the greater the edge betweenness of the node.).

**Figure 9:**
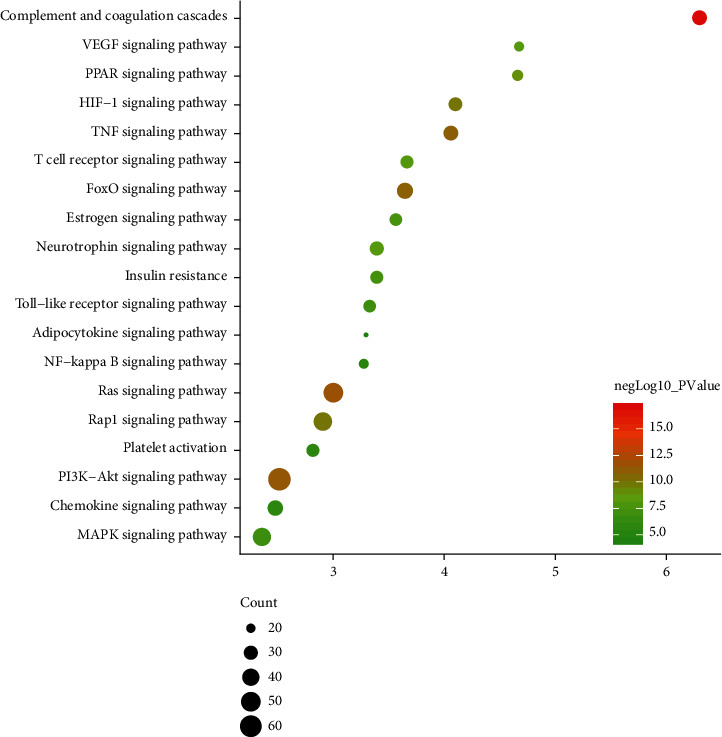
Signaling pathways of biological processes (The *X*-axis is fold enrichment.).

Nineteen (19) CI-related signal pathways were returned. The relationship among signaling pathways, targets, and components is shown in Figure 8. The details of signaling pathways are shown in Figure 9 and Table S5. The number of targets regulated by the components of *Radix Rhei Et Rhizome* is shown in Table 2.

#### 3.2.4. Reactome Pathways of *Radix Rhei Et Rhizome*-CI PPI Network

**Figure 10:**
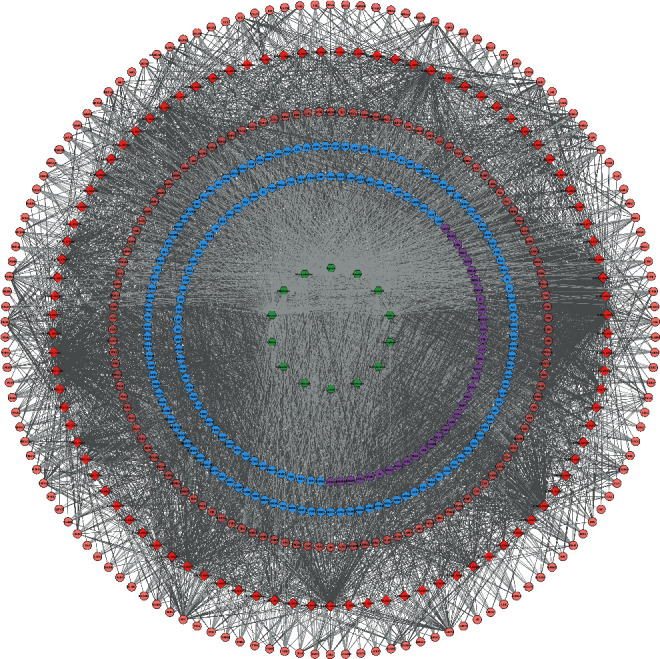
Reactome pathways of *Radix Rhei Et Rhizome*-CI PPI Network (Green hexagons stand for potential compounds. Blue, pink, and purple circles stand for CI genes, *Radix Rhei Et Rhizome* targets, and *Radix Rhei Et Rhizome*-CI targets, respectively. Red diamonds stand for reactome pathways. Dark lines stand for relationships among reactome pathways and targets. Light lines stand for relationships among herbs and targets.).

**Figure 11:**
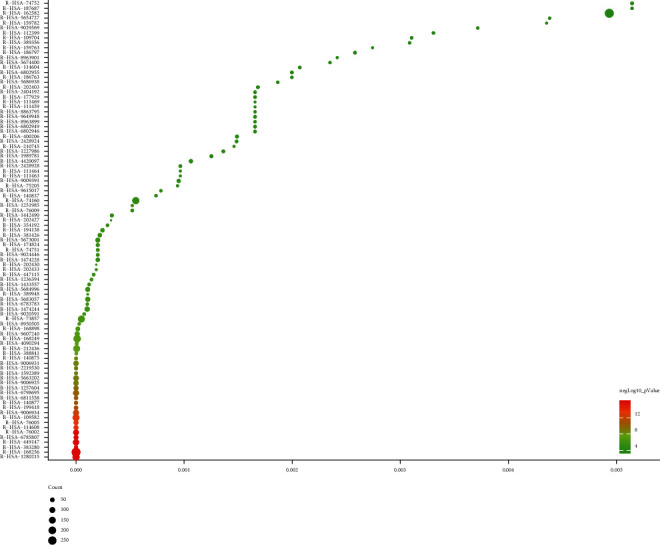
Reactome pathways of biological processes (The *X*-axis is FDR.).

Ninety-three (93) CI-related signal pathways were returned. The relationship among reactome pathways, targets, and components is shown in Figure 10. The details of reactome pathways are shown in Figure 11 (Table S6). The number of targets regulated by the components of *Radix Rhei Et Rhizome* is shown in Table 3.

The main biological processes, signaling pathways, and reactome pathways are shown in Figures 7, 9, and 10, respectively. Current studies have found that the occurrence and development of CI have slowed blood flow and vascular sclerosis in the cerebral vessels [55]. Astrocytes, endothelial cells, and pericytes constitute the neurovascular units required for neuronal metabolism [56, 57]. When CI occurs, the neurovascular unit is abnormal: hypoxia results in the dysfunction of the endothelial cell barrier of the blood-brain barrier, leading to a decrease in intracellular cAMP levels and an increase in vascular permeability [58, 59]. Ischemia and reperfusion cause various cells in the neurovascular unit to initiate cell death programs, including apoptosis, autophagy-related cell death, iron death, cell scoring, and necrosis [60, 61]. During CI, these cells contribute to postischemic inflammation at multiple stages of the ischemic cascade [62]. In the inflammatory response, microglia and astrocytes and infiltrating immune cells release a variety of inflammatory factors, including cytokines, chemokines, enzymes, and free radicals, which not only cause brain damage but also affect brain tissue repair [63, 64]. Recent studies have also shown that anti-inflammatory is an important treatment strategy for CI [65]. After thrombolysis, oxidative stress becomes the central link in cerebral ischemia-reperfusion. During reperfusion, oxygen is replenished, which is essential for maintaining the viability of neurons [66]. However, prooxidase and mitochondria also use oxygen as a substrate, and a large amount of oxygen free radicals (oxidants) are generated during reperfusion. Endogenous antioxidant enzymes, including SOD, can clear oxidants and reduce oxidant-induced brain damage [67, 68].

The network pharmacology strategy was used above to predict the mechanism of *Radix Rhei Et Rhizome* intervention in CI. In order to further explore it, the previous proteomics data were analyzed in depth. The proteomics data come from reference [69].

### 3.3. Bioinformatics Analysis of Proteomics Proteins

#### 3.3.1. Proteomics Proteins' PPI Network

**Figure 12:**
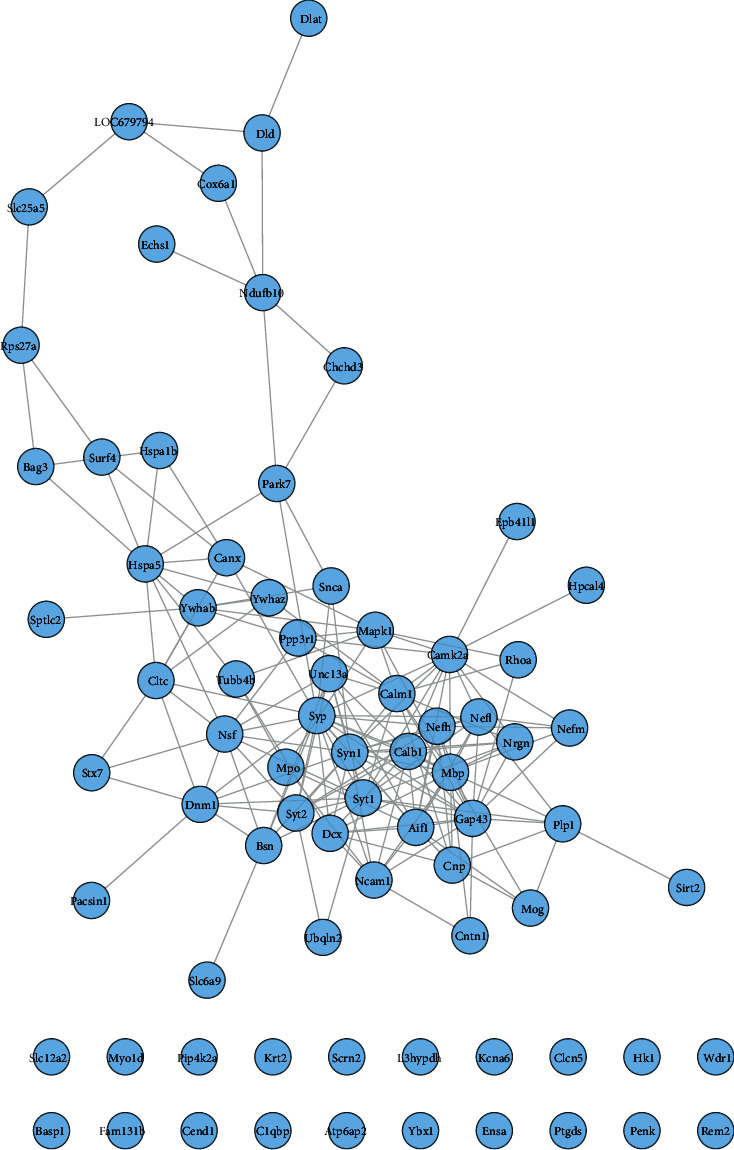
Proteomics proteins' PPI network.

The proteomics proteins of reference [69] are shown in Table S2. A total of 76 proteins were input into String for PPI data. The proteomics proteins' PPI data were composed of 76 proteomics protein nodes and 182 edges (Figure 12).

#### 3.3.2. Enrichment Analysis Results

**Figure 13:**
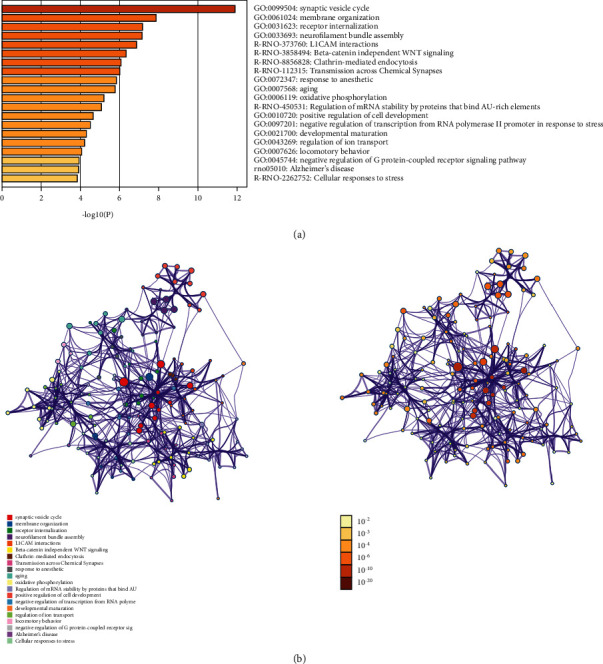
The Metascape results: (a) top biological processes, signaling pathways, and reactome pathways of the proteomics proteins' PPI network and (b): PPI networks colored by enrichment results or *P*-values.

**Figure 14:**
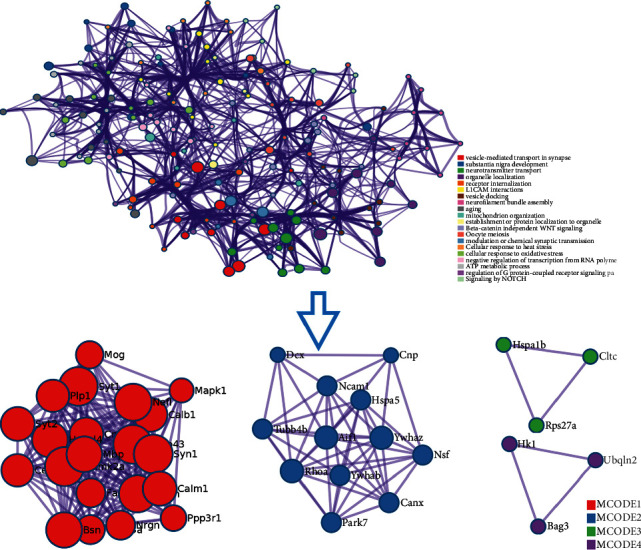
Clusters of proteomics proteins' PPI network.

DAVID and Metascape (http://metascape.org/gp/index.html#/main/step1) were utilized to analyze the proteins in the proteomics proteins' PPI network (Figure 13). The details of biological processes, signaling pathways, and reactome pathways are shown in Table S7. The clusters are shown in Figure 14.

The top 20 proteins in Figure 12 were Syp, Syn1, Mbp, Gap43, Aif1, Camk2a, Syt1, Calm1, Calb1, Nsf, Nefl, Hspa5, Nefh, Ncam1, Dcx, Unc13a, Mapk1, Syt2, Dnm1, and Cltc. These proteins may be the key targets of *Radix Rhei Et Rhizome* in the treatment of CI. As a specific marker protein of synaptic vesicles, synaptophysin (SYP) is a sign of synapse occurrence. Its density and distribution can indirectly reflect the number and distribution of synapses in the body [70, 71]. Studies have found that after CI in rats, the synaptophysin immune response was significantly enhanced compared with the sham operation group, and the expression reached a peak at 2 weeks after cerebral ischemia, and it significantly decreased at 3 weeks [72]. This experimental study found that after Radix Rhei Et Rhizome treatment, its expression was significantly increased compared with the model group, indicating that Radix Rhei Et Rhizome may resist synapse damage or promote synaptic regeneration after cerebral ischemia. Synapse protein I gene (SYN1) mainly mediates the delivery of synaptic vesicles and circulation [73] and plays an important role in neurodegenerative diseases [74], such as Alzheimer's disease, Parkinson's disease, and amyotrophic lateral sclerosis. This study suggests that SYN1 may be a potential drug target for neurodegenerative diseases caused by CI, among which *Radix Rhei Et Rhizome* can reduce CI by interfering with SYN1.

GAP-43 is a calmodulin-binding phosphoprotein that has been isolated and identified in recent years [75]. In the process of neuron development and regeneration, GAP-43 is synthesized in large quantities along with the growth of axons and is a marker of axon growth. The expression product is mainly located on the plasma membrane surface of the axon growth cone [76]. Studies have shown that the increase in GAP-43 content in the penumbra of the ischemic penumbra of one middle cerebral artery 3 to 14 days after the embolization of the middle cerebral artery is synchronized with the recovery of the function of the affected limb [77]. Another study found that the expression of GAP-43 in the brain increased significantly after cerebral infarction, peaked at 1 week after ischemia-reperfusion, and began to decrease after 2 weeks [78, 79]. This experiment found that *Radix Rhei Et Rhizome* can significantly enhance the expression of GAP-43 in the cortical ischemic penumbra, suggesting that *Radix Rhei Et Rhizome* can effectively promote the regeneration of axons in CI model rats and induce the recovery of nerve function.

MBP is mainly located on the serosal surface of the myelin sheath and is the main protein of the myelin sheath of the central nervous system. Its main function is to maintain the integrity and functional stability of the myelin sheath of the central nervous system, and its neural tissue specificity is high [80]. When acute cerebral infarction (ACI) occurs, when the human central nervous system is damaged, the blood-brain barrier function is unbalanced, which increases its permeability, and MBP can easily pass through the blood-brain barrier and be released into the blood. At present, the detection of serum NSE and MBP expression levels is of great value in assessing the prognosis of ACI disease [81]. The results of this study showed that the MBP level of the CI group increased rapidly and the MBP level decreased after Radix Rhei Et Rhizome treatment, suggesting that Radix Rhei Et Rhizome treatment of the ACI rat model can promote the disease outcome and reduce the MBP level.

Allogeneic inflammatory factor-1 (AIF-1) is a 17 kDa cytoplasmic calcium-binding inflammatory response scaffold protein, which is mainly expressed in immune cells [82]. AIF-1 affects the immune system at several key points, thereby regulating inflammatory diseases [83]. AIF-1 promotes the expression of inflammatory mediators such as cytokines, chemokines, and inducible nitric oxide synthase (iNOS) and promotes the proliferation and migration of inflammatory cells. Current research shows that it regulates central nervous system (CNS) damage [82, 83]. The results of this study showed that the level of cerebral infarction (AIF-1) increased rapidly, and the level of AIF-1 decreased after *Radix Rhei Et Rhizome* treatment. It is suggested that *Radix Rhei Et Rhizome* can promote the outcome of CI and reduce the level of inflammation.

The alpha (CAMK2A) of calcium/calmodulin-dependent protein kinase II (CaMKII) plays a key role in neuronal plasticity and brain learning and memory [84, 85]. After *Radix Rhei Et Rhizome* intervention, the expression of CAMK2A was upregulated. The main biological function of Syt1 is to trigger vesicle fusion [86], which is related to the molecular mechanism of neuronal endocytosis and exocytosis coupling [87]. This study found that Syt1 was upregulated after *Radix Rhei Et Rhizome* intervention. Calmodulin 1 (CALM1) is highly expressed in the human brain tissue, and its biological function is mainly related to axon transmission [88]. In addition, the Calm1 signaling pathway is crucial for the migration of precerebellar neurons in mice [89]. Calm1-L plays a functional role in the central and peripheral nervous system [90]. This study found that Calm1 was upregulated after *Radix Rhei Et Rhizome* intervention. A recent study also showed that CALM1 rs3179089 gene polymorphism is associated with CI in Chinese Han population [91].

Calbindin 1 (Calb1) acts as a buffer, sensor, and transporter of intracellular Ca^2+^. Different types of hippocampal neurons have different Calb1 concentrations. Since Calb1 can inhibit the increase of free Ca^2+^, it accelerates the collapse of the Ca^2+^ gradient after the influx of Ca^2+^ stops [92, 93]. Current research shows that it plays a role in neurotransmitter and hormone release, neuron differentiation, brain wiring, and neuron development [94, 95]. The results of this study showed that the level of Calb1 was downregulated in the CI group, and the level of Calb1 was upregulated after Radix Rhei Et Rhizome treatment. It is suggested that *Radix Rhei Et Rhizome* may promote disease outcome through neurotransmitter and other methods.

*N*-ethylmaleimide-sensitive fusion protein (NSF) is an ATPase that plays an important role in intracellular membrane vesicle transport [96]. It is highly conservative in evolution and participates in the secretion process of different species and different cell types [96, 97]. Current research shows that it plays an important role in the process of neurotransmitter release by synaptic vesicle exocytosis at presynaptic nerve terminals [97, 98]. The results of this study showed that the level of NSF in the cerebral infarction group was downregulated and the level of NSF was upregulated after treatment.

Neurofilament light chain (Nefl) belongs to one of the main subtypes of neurofilament protein. It is the main cytoskeleton structural protein of neurons, which is distributed in axons [99, 100]. Nefl is also an important indicator for judging acute axonal injury [99]. In clinical studies of relapsing multiple sclerosis, Nefl is used as an effective evaluation index for drug anti-inflammatory therapy [101]. Recent studies have shown that emodin activates mTOR and Notch pathways in hypoxic PC12 cells by inhibiting Nefl [102]. In this study, the expression level of Nefl in CI model rats was higher but decreased after intervention, indicating that there is acute axon damage in acute stroke and *Radix Rhei Et Rhizome* may be able to protect axons.

The endoplasmic reticulum stress chaperone protein HSPA5 is mainly related to endoplasmic reticulum stress. Current studies have shown that endoplasmic reticulum stress can induce autophagy activation [103]. Previous studies have confirmed that in the mouse brain I/R model, the expression of HSPA5 protein is increased, and it has a neuroprotective effect [104]. At present, by injecting HSPA5 siRNA into the anterior ventricle of CI mice, the expression of LC3-1/LC3-I is significantly reduced, and it will also lead to the loss of nerve cells in the cerebral ischemic cortex of mice and aggravation of neurobehavioral damage. This is similar to the effect of the autophagy inhibitor 3-MA, indicating that HSPA5-mediated autophagy may play a neuroprotective effect in mouse I/R [105, 106]. The results of this study showed that the level of HSPA5 in the CI group was downregulated, but after treatment, the level of HSPA5 was upregulated.

NCAM1 is a member of the cell adhesion molecule family and is a molecular cleavage of the immunoglobulin superfamily. NCAM1 is a membrane protein that includes three subtypes: NCAM-120, NCAM-140, and NCAM-180. It is mainly expressed in the nervous system and is involved in regulating the function of nerve cells and neuron migration [107]. NCAM1 is expressed in neural stem cells. In addition, astrocytes also express many adhesion molecules, such as VCAM1, NCAM1, and ICAM1, which represent many potential drug targets for inflammatory diseases of the central nervous system [108–110]. This study showed that the level of NCAM1 in the CI group was downregulated. After *Radix Rhei Et Rhizome* treatment, the level of NCAM1 was upregulated, suggesting that *Radix Rhei Et Rhizome* may inhibit adhesion molecules and is related to inflammatory factors.

Dcx is a microtubule-associated phosphoprotein, which is specifically expressed in newborn neuroblasts and immature neurons in DG [111, 112]. Therefore, Dcx has been widely used to label the cell bodies, processes, and growth cones of newborn neurons. Studies have reported that after CI, Dcx strengthens the differentiation of nerve cells in the DG area of the hippocampus and promotes the rehabilitation of nerve function [113]. This study showed that the level of Dcx in the CI group was downregulated, and after treatment, the level of Dcx was upregulated, suggesting that *Radix Rhei Et Rhizome* may regulate the generation of new neurons and promote the outcome of CI.

The limitation of this study is that although the pharmacokinetic parameters are used to predict the composition of *Radix Rhei Et Rhizome* and the composition was supplemented as much as possible by searching the literature, due to the limitations of the current detection technology, there are still active ingredients that may not be included. Since the intestinal flora may metabolize and secondary modify the active components of *Radix Rhei Et Rhizome*, these components may be traced in the blood. In the future, better technology is needed to detect these components. In addition, although this study analyzed the main active components of *Radix Rhei Et Rhizome* in the treatment of CI through chemoinformatics and explored its possible synergistic effects, there is still a lack of in vivo and in vitro experiments related to their intervention in CI. In the future, we will explore the synergistic compatibility of these components in the CI in vitro model and the CI in vivo model and look forward to further development of new drugs for the treatment of CI, laying the foundation for its clinical application.

Our previous research evaluated the therapeutic effect of *Radix Rhei Et Rhizome* on cerebral hemorrhage [114], while this study explored the mechanism of Radix Rhei Et Rhizome in the treatment of CI, and this study found that Radix Rhei Et Rhizome may regulate the synaptic remodeling and the regeneration of nerve cell axons after cerebral ischemia. Compared with previous research [114], this study explored the mechanism of *Radix Rhei Et Rhizome* intervention in CI. This study found that *Radix Rhei Et Rhizome* may treat CI through biological process (such as platelet degranulation, cell migration, fibrinolysis, platelet activation, hypoxia, angiogenesis, endothelial cell apoptosis, coagulation, and neuronal apoptosis), signaling pathways (such as Ras, PI3K-Akt, TNF, FoxO, HIF-1, and Rap1), and reactome pathways (such as inflammatory cytokines, platelet activation, response to elevated platelet cytoplasmic Ca^2+^, and hemostasis).

## 4. Conclusion

*Radix Rhei Et Rhizome* may play the therapeutic role for CI through regulating biological modules such as synaptic vesicles and neurotransmitter secretion and transport, energy metabolism, neuronal programmed death (apoptotic autophagy) module, calcium ion regulation of exocytosis and cytoplasmic calcium ion release, endoplasmic reticulum oxidative stress, and neuroplasticity (neuron and synaptic plasticity).

## Figures and Tables

**Table 1:** Clusters of *Radix Rhei Et Rhizome*-CI PPI network.

Cluster	Score	Nodes	Edges	Targets and genes
1	44	67	1452	IGF1, NOS3, SERPINE1, MMP2, CRP, CCL5, VWF, PTEN, CASP8, MYD88, CD40, TLR4, MMP1, PLG, NGF, CTGF, HRAS, HSPA4, HIF1A, MMP9, RHOA, ANXA5, CREB1, SOD2, REN, CYCS, GRB2, MAPK1, MMP3, BCL2L1, IL2, TGFB1, ADIPOQ, AGT, FGF2, SELE, CD40LG, PTGS2, ALB, IL10, IL1B, HGF, MAP2K1, CCL2, IL4, ACE, CAT, THBS1, CXCL8, PPARG, AR, JAK2, ESR1, HMOX1, MMP7, CSF3, EDN1, BDNF, EGFR, SELP, MAPK14, CDC42, ICAM1, MMP13, STAT1, APOE, APP
2	25.821	79	1007	AKT1, XIAP, PIK3CA, SRC, F13A1, ALDOA, F3, NFKB1, AKT2, PTPN11, FGA, FGG, AGTR1, NR3C1, AIF1, PPBP, HSP90AA1, SERPINF2, NQO1, NOS2, ABL1, FGB, SOCS3, CD34, MIF, BMP2, PDGFRB, PDGFB, MET, HMGB1, IL6, KIT, PGF, MDM2, HPGDS, TEK, MBP, LGALS3, ENG, SOD1, MAPK8, TNF, PLAU, GFAP, PTK2, INS, RAF1, IL1A, GSK3B, TP53, CASP3, FAS, PTPN1, PROS1, TGFB2, VEGFA, HRG, ELANE, IGFBP3, TLR3, MAPK10, PGR, NCF1, CCNA2, CASP1, FGF1, IGF1R, F8, PLAUR, RETN, ADAM17, LDLR, KDR, CFD, MMP14, F5, PARP1, S100B, EGF
3	6.846	27	89	PAH, LPL, ARSA, RNASE2, APCS, CTSK, IMPDH1, CTSL, PON1, FABP4, RBP4, LPA, BPI, CST3, PROZ, RNASE3, SCARB1, F7, LIPG, FABP5, GM2A, PROC, HMGCR, GC, HABP2, HEXB, APOA1
4	5.706	35	97	F11, SERPINC1, HP, APOM, AKR1B1, HK1, HSP90AB1, SERPINA1, APOB, ENO2, ABCA1, OLR1, ATIC, PIK3CG, PLA2G7, CDK6, F10, ANG, SERPIND1, ZAP70, RHEB, LCAT, BACE1, HCK, MMP12, BTK, TTR, COG2, LIPC, ITGAL, AURKA, SYK, BRAF, APOC2, F12
5	4.783	24	55	PNP, BHMT, HADH, NR1I3, NT5M, SHMT1, YARS, TPI1, RAN, STS, UCK2, UCP3, ACADM, TYMS, RXRA, AHCY, LDHB, IVD, CYP1A1, PDE3B, PSPH, ADK, SULT1A1, AKR1C3
6	4	21	40	G6PD, MAP2, RAC2, GLRX, PTGER3, GSTP1, RHOB, RHOD, ASAH1, DCX, PSAP, CHIT1, CALM1, CRYZ, CTSS, CDA, PRDX1, HTR1A, LTA4H, QPCT, NPY
7	4	8	14	GLO1, GMPR, APRT, APEX1, IMPDH2, UMPS, DTYMK, TK1
8	4	4	6	RXRB, THRB, RARG, RARB
9	4	5	8	GALE, GNPDA2, UAP1, GALK1, GNPDA1
10	3.818	34	63	APOH, FBN1, TIMP3, THBD, HSPA8, EPHA2, INSR, PF4, ADAM10, PLAT, F2, RAC1, APAF1, PIK3R1, ERBB4, GSR, CASP7, CDK2, CHEK1, NOS1, EIF4E, HSPA1A, ESR2, CCL11, CYBA, FGFR2, ARG1, JAK3, APOA2, CSK, LCK, TGFBR1, MMP8, LCN2
11	3.75	17	30	GSTO1, CYP2C8, AGXT, GSTA1, FCAR, PCK1, BST1, ADH1C, P2RY12, ITK, ITGB2, CYP3A5, ADH1B, PYGL, NR1H4, HLA-DRB1, CYP2C9
12	3.571	15	25	SLC9A1, FABP1, KAT2B, SAA1, APOA5, TBXA2R, PPARA, ACTA2, TGFBR2, PRKACA, CPB2, RARA, EDNRA, COL3A1, NR1H3
13	3.333	4	5	GSTM2, SOD3, GSTA3, GSTM1
14	3	3	3	GPI, PKLR, ALDOB
15	3	3	3	SMARCA1, DOT1L, HDAC8
16	3	3	3	MAOA, PNMT, MAOB
17	3	3	3	AMY1A, AMY1B, AMY1C
18	2.889	10	13	GART, DCK, HFE, FECH, DHFR, PDE4B, ALAD, CLPP, PDE4D, DHODH

**Table 2:** The number of targets regulated by the components of *Radix Rhei Et Rhizome.*

Components	Number of targets
Sennoside A	103
Palmidin A	99
Emodin	98
Toralactone	96
Mutatochrome	96
Rhein	95
Physcion	95
Eupatin	93
(-)-Catechin	91
Aloe-emodin	88
Chrysophanol	86
Beta-sitosterol	85
Daucosterol	85
Danthron	53

**Table 3:** The number of targets regulated by the components of *Radix Rhei Et Rhizome*.

Components	Number of targets
Palmidin A	222
Sennoside A	213
Toralactone	212
Emodin	209
Rhein	209
Eupatin	207
(-)-Catechin	205
Aloe-emodin	202
Physcion	202
Mutatochrome	193
Chrysophanol	178
Beta-sitosterol	171
Daucosterol	168
Danthron	116

## Data Availability

The data used to support the findings of this study are included within the article and the supplementary information files.

## Supplementary Materials

Supplementary Materials Table S1: potential targets for potential compounds; Table S2: proteomics data; Table S3: CI gene; Table S4: enrichment analysis of clusters based on gene ontology (GO) annotation of *Radix Rhei Et Rhizome*-CI PPI network; Table S5: pathway enrichment analysis of *Radix Rhei Et Rhizome*-CI PPI network; Table S6: reactome pathways of *Radix Rhei Et Rhizome*-CI PPI network; and Table S7: the biological processes, signaling pathways, and reactome of proteomics proteins' PPI network.
