# Supplementary material for: Mechanism of Radix Rhei Et Rhizome Intervention in Cerebral Infarction: A Research Based on Chemoinformatics and Systematic Pharmacology
Source: Evid Based Complement Alternat Med. 2021 Sep 6;2021:6789835. doi: 10.1155/2021/6789835 (PMC8440083; doi:10.1155/2021/6789835)
Supplement: Supplementary Materials — Table S1: potential targets for potential compounds; Table S2: proteomics data; Table S3: CI gene; Table S4: enrichment analysis of clusters based on gene ontology (GO) annotation of Radix Rhei Et Rhizome-CI PPI network; Table S5: pathway enrichment analysis of Radix Rhei Et Rhizome-CI PPI network; Table S6: reactome pathways of Radix Rhei Et Rhizome-CI PPI network; and Table S7: the biological processes, signaling pathways, and reactome of proteomics proteins' PPI network. [file 6789835.f1.zip › 6789835.f1/Table S1.pdf]

Table S1 Potential targets for potential compounds

Compounds	Compounds Targets
Toralactone	CA1
Toralactone	BACE1
Toralactone	BCHE
Toralactone	ALB
Toralactone	CMA1
Toralactone	AR
Toralactone	PIM1
Toralactone	PPIA
Toralactone	GSTP1
Toralactone	MAPK10
Toralactone	F2
Toralactone	BMP2
Toralactone	CYP19A1
Toralactone	STS
Toralactone	CA2
Toralactone	PDE4B
Toralactone	TREM1
Toralactone	CDK2
Toralactone	HSPA8
Toralactone	PDPK1
Toralactone	CCNA2
Toralactone	CFB
Toralactone	APOA2
Toralactone	MAOB
Toralactone	NR1H2
Toralactone	FKBP1A
Toralactone	CDK5R1
Toralactone	AKR1B1
Toralactone	MAPK8
Toralactone	ESR1
Toralactone	PAH
Toralactone	KIF11
Toralactone	ADAM17
Toralactone	HSP90AA1
Toralactone	TYMS
Toralactone	CHEK1
Toralactone	EGFR
Toralactone	TTR
Toralactone	ESRRG
Toralactone	NOS3
Toralactone	BCAT2
Toralactone	RXRA
Toralactone	MIF
Toralactone	PPP5C
Toralactone	CTSB
Toralactone	MMP13

Toralactone	FNTA
Toralactone	DUSP6
Toralactone	GSR
Toralactone	EPHB4
Toralactone	BRAF
Toralactone	PPARG
Toralactone	F10
Toralactone	MAPK14
Toralactone	CFD
Toralactone	PPARD
Toralactone	SRC
Toralactone	AKR1C2
Toralactone	SHBG
Toralactone	PDE3B
Toralactone	ANG
Toralactone	PDE5A
Toralactone	PLK1
Toralactone	SOD2
Toralactone	NQO1
Toralactone	PRKACA
Toralactone	PGR
Toralactone	MTAP
Toralactone	DHODH
Toralactone	MMP3
Toralactone	GC
Toralactone	TGFBR1
Toralactone	KDR
Toralactone	ADH1C
Toralactone	AKR1C1
Toralactone	ANXA5
Toralactone	LTA4H
Toralactone	FGFR1
Toralactone	CSNK1G2
Toralactone	MMP8
Toralactone	SULT2A1
Toralactone	NR3C2
Toralactone	PTPN1
Toralactone	AKT1
Toralactone	CASP7
Toralactone	LCK
Toralactone	METAP2
Toralactone	SEC14L2
Toralactone	SORD
Toralactone	CDK6
Toralactone	ADK
Toralactone	HSD11B1
Toralactone	PDE4D
Toralactone	CTNNA1

Toralactone	FGFR2
Toralactone	PAK7
Toralactone	PTPN11
Toralactone	PGF
Toralactone	ADH
Toralactone	NR1H4
Toralactone	CBR1
Toralactone	HSD17B1
Toralactone	CYP2C9
Toralactone	CES1
Toralactone	SYK
Toralactone	HPGDS
Toralactone	ESR2
Toralactone	HDAC8
Toralactone	CDA
Toralactone	EPHX2
Toralactone	IGF1R
Toralactone	IMPDH2
Toralactone	HMGCR
Toralactone	AMY2A
Toralactone	AMD1
Toralactone	DAPK1
Toralactone	DPP4
Toralactone	YARS
Toralactone	AKR1C3
Toralactone	ISG20
Toralactone	LSS
Toralactone	GSK3B
Toralactone	ITK
Toralactone	CCNT1
Toralactone	CTSK
Toralactone	CASP3
Toralactone	ERRA
Toralactone	RORA
Toralactone	JAK3
Toralactone	AURKA
Toralactone	TRAPPC3
Toralactone	NQO2
Toralactone	RHOA
Toralactone	DHFR
Toralactone	HCK
Toralactone	DTYMK
Toralactone	SHMT1
Toralactone	TPI1
Toralactone	MET
Toralactone	F7
Toralactone	PDK2
Toralactone	AHCY

Toralactone	HPN
Toralactone	PCK1
Toralactone	THRB
Toralactone	SERPINA1
Toralactone	TK1
Toralactone	ELANE
Toralactone	ARSA
Toralactone	RNASE3
Toralactone	MMP12
Toralactone	TPH1
Toralactone	FABP4
Toralactone	PPARA
Toralactone	GSTA1
Toralactone	ABO
Toralactone	NR1H3
Toralactone	MTHFD1
Toralactone	WAS
Toralactone	XIAP
Toralactone	BIRC7
Toralactone	AKT2
Toralactone	PYGL
Toralactone	ACPP
Toralactone	TNK2
Toralactone	RAB11A
Toralactone	IMPA1
Toralactone	IL2
Toralactone	RBP4
Toralactone	PADI4
Toralactone	PAK6
Toralactone	NOS2
Toralactone	MAPKAPK2
Toralactone	BST1
Toralactone	UMPS
Toralactone	CYP2C8
Toralactone	CSK
Toralactone	REN
Toralactone	UCK2
Toralactone	ZAP70
Toralactone	SELP
Toralactone	MMP2
Toralactone	ACADM
Toralactone	PNMT
Toralactone	MAPK1
Toralactone	FABP3
Toralactone	TTPA
Toralactone	NR1I2
Toralactone	NR3C1
Toralactone	ALAD

Toralactone	SETD7
Toralactone	JAK2
Toralactone	PLA2G2A
Toralactone	CTSS
Toralactone	GALK1
Toralactone	FABP6
Toralactone	DCK
Toralactone	ARHGAP1
Toralactone	THRA
Toralactone	IGF1
Toralactone	MMP9
Toralactone	ARG1
Toralactone	CTSF
Toralactone	SULT1E1
Toralactone	MME
Toralactone	PLEKHA4
Toralactone	GSTT2
Toralactone	EIF4E
Toralactone	MAN1B1
Toralactone	HNF4G
Toralactone	CTSG
Toralactone	PNP
Toralactone	CHIT1
Toralactone	ERBB4
Toralactone	NMNAT1
Toralactone	SULT2B1
Toralactone	APRT
Toralactone	RAB5A
Toralactone	PPCDC
Toralactone	CRABP2
Toralactone	TGM3
Toralactone	HNMT
Toralactone	CLK1
Toralactone	FECH
Toralactone	NT5M
Toralactone	KAT2B
Toralactone	SDS
Toralactone	PRKCQ
Toralactone	RARG
Toralactone	F11
Toralactone	OTC
Toralactone	FDPS
Toralactone	GPI
Toralactone	PKLR
Toralactone	CCL5
Toralactone	DPEP1
Toralactone	MAP2K1
Toralactone	GSTO1

Toralactone	ABL1
Toralactone	OAT
Toralactone	GCK
Toralactone	S100A9
Toralactone	STAT1
Toralactone	PCMT1
Toralactone	TEK
Toralactone	CDC42
Toralactone	VDR
Toralactone	B3GAT1
Toralactone	BHMT
Toralactone	LYZ
Toralactone	FABP7
Toralactone	CBS
Toralactone	LGALS3
Toralactone	HRAS
Toralactone	HINT1
Toralactone	RARA
Toralactone	GART
Toralactone	NME2
Toralactone	IMPDH1
Toralactone	KIT
Toralactone	LCN2
Toralactone	DUT
Toralactone	RARB
Toralactone	PIK3R1
Toralactone	GSTA3
Toralactone	PPP1CC
Toralactone	FOLH1
Toralactone	HEXB
Toralactone	GP1BA
Toralactone	BLVRB
Toralactone	TGFB2
Toralactone	MMP16
Toralactone	GLO1
Toralactone	GSTM1
Toralactone	HPRT1
Toralactone	RHEB
Toralactone	RXRB
Toralactone	SELE
Toralactone	RNASE2
Toralactone	RAF1
Toralactone	NDST1
Toralactone	GSTM2
Toralactone	CASP1
Toralactone	DOT1L
Toralactone	FKBP1B
Toralactone	HMOX1

Toralactone	SSE1
Toralactone	HAGH
Toralactone	ATIC
Toralactone	ARL5A
Toralactone	FKBP3
Toralactone	BCL2L1
Toralactone	WARS
Toralactone	AMY1A
Toralactone	AMY1B
Toralactone	AMY1C
Sennoside A	CA12
Sennoside A	EEA1
Sennoside A	CASP3
Sennoside A	BMP2
Sennoside A	TTR
Sennoside A	CDK2
Sennoside A	GSTP1
Sennoside A	MAPK10
Sennoside A	CMA1
Sennoside A	PDE4B
Sennoside A	FAP
Sennoside A	PPIA
Sennoside A	GBA
Sennoside A	HSD17B11
Sennoside A	PTPN1
Sennoside A	LCN2
Sennoside A	CTSD
Sennoside A	BCHE
Sennoside A	AURKA
Sennoside A	PNP
Sennoside A	KDR
Sennoside A	ICAM2
Sennoside A	TGFBR2
Sennoside A	RTN4R
Sennoside A	FCAR
Sennoside A	PIM1
Sennoside A	AKR1B1
Sennoside A	FKBP1A
Sennoside A	PGR
Sennoside A	METAP1
Sennoside A	GSR
Sennoside A	CHIT1
Sennoside A	CLPP
Sennoside A	HSPA8
Sennoside A	LGALS7
Sennoside A	CA2
Sennoside A	PKLR
Sennoside A	ALB

Sennoside A	APOA2
Sennoside A	AKR1C2
Sennoside A	BACE1
Sennoside A	CTSV
Sennoside A	EPHB4
Sennoside A	CA1
Sennoside A	ESR1
Sennoside A	CASP7
Sennoside A	NR1H2
Sennoside A	HCK
Sennoside A	IMPA1
Sennoside A	TREM1
Sennoside A	CSNK2A1
Sennoside A	RNASEL
Sennoside A	THRB
Sennoside A	PLAU
Sennoside A	IGF1R
Sennoside A	LDHB
Sennoside A	SRC
Sennoside A	MAPK1
Sennoside A	PSPH
Sennoside A	ANG
Sennoside A	DHFR
Sennoside A	CPB1
Sennoside A	CCNA2
Sennoside A	ME2
Sennoside A	SHBG
Sennoside A	MMP8
Sennoside A	CHEK1
Sennoside A	SULT2A1
Sennoside A	F2
Sennoside A	PNPO
Sennoside A	MMP3
Sennoside A	LSS
Sennoside A	GC
Sennoside A	BMP7
Sennoside A	MTAP
Sennoside A	APCS
Sennoside A	CRAT
Sennoside A	MMP13
Sennoside A	CFD
Sennoside A	EGFR
Sennoside A	MAPK14
Sennoside A	CTSB
Sennoside A	AMY2A
Sennoside A	BCAT2
Sennoside A	IGLV2-8
Sennoside A	FABP4

Sennoside A	AZGP1
Sennoside A	HSD17B1
Sennoside A	ADH1B
Sennoside A	F10
Sennoside A	NOS3
Sennoside A	MAOB
Sennoside A	PDE4D
Sennoside A	GALK1
Sennoside A	PPP5C
Sennoside A	CFB
Sennoside A	CSNK1G2
Sennoside A	PYGL
Sennoside A	SORD
Sennoside A	NMNAT1
Sennoside A	AHCY
Sennoside A	HSP90AA1
Sennoside A	REN
Sennoside A	HSD11B1
Sennoside A	AKR1C3
Sennoside A	KIF11
Sennoside A	PAH
Sennoside A	PDE3B
Sennoside A	NCS1
Sennoside A	PDPK1
Sennoside A	OTC
Sennoside A	BRAF
Sennoside A	DHODH
Sennoside A	ARF1
Sennoside A	C1S
Sennoside A	TGFBR1
Sennoside A	NR3C2
Sennoside A	IMPDH2
Sennoside A	AKT1
Sennoside A	MMP12
Sennoside A	GALE
Sennoside A	FGF1
Sennoside A	TGM3
Sennoside A	EPHA2
Sennoside A	PARP1
Sennoside A	TPI1
Sennoside A	ANXA5
Sennoside A	PLA2G2A
Sennoside A	HSPA1A
Sennoside A	TYMS
Sennoside A	PCK1
Sennoside A	DPP4
Sennoside A	MAPKAPK2
Sennoside A	TGM2

Sennoside A	CLIC1
Sennoside A	PDE5A
Sennoside A	AR
Sennoside A	RNASE3
Sennoside A	PLK1
Sennoside A	HMGCR
Sennoside A	LGALS2
Sennoside A	UCK2
Sennoside A	ABO
Sennoside A	SOD2
Sennoside A	CTSK
Sennoside A	RXRA
Sennoside A	PDHB
Sennoside A	RHEB
Sennoside A	LCK
Sennoside A	GSK3B
Sennoside A	BAG1
Sennoside A	B3GAT1
Sennoside A	PPARG
Sennoside A	ADH1C
Sennoside A	MIF
Sennoside A	INSR
Sennoside A	MTHFD1
Sennoside A	DAPK1
Sennoside A	ISG20
Sennoside A	TYMP
Sennoside A	AKR1C1
Sennoside A	F7
Sennoside A	MAPK8
Sennoside A	BST1
Sennoside A	SERPINA1
Sennoside A	ESRRG
Sennoside A	MMP7
Sennoside A	ACPP
Sennoside A	RAC2
Sennoside A	ITK
Sennoside A	YARS
Sennoside A	DCK
Sennoside A	CDA
Sennoside A	C1R
Sennoside A	XIAP
Sennoside A	FNTA
Sennoside A	PAK7
Sennoside A	HDAC8
Sennoside A	REG1A
Sennoside A	HK1
Sennoside A	ALDOA
Sennoside A	FGFR2

Sennoside A	NQO2
Sennoside A	ATOX1
Sennoside A	PAK6
Sennoside A	CAT
Sennoside A	IGF1
Sennoside A	CBR1
Sennoside A	DUSP6
Sennoside A	NQO1
Sennoside A	PRKACA
Sennoside A	SELP
Sennoside A	TNK2
Sennoside A	GPI
Sennoside A	AMD1
Sennoside A	CES1
Sennoside A	MET
Sennoside A	FGFR1
Sennoside A	ALDH2
Sennoside A	GSTA1
Sennoside A	SYK
Sennoside A	CDK7
Sennoside A	RAB11A
Sennoside A	GLRX
Sennoside A	PIK3CG
Sennoside A	KIT
Sennoside A	TTPA
Sennoside A	PLEKHA4
Sennoside A	PLA2G10
Sennoside A	METAP2
Sennoside A	DTYMK
Sennoside A	MAN1B1
Sennoside A	ARSA
Sennoside A	GSTT2
Sennoside A	IMPDH1
Sennoside A	CTSF
Sennoside A	CDK6
Sennoside A	CYP2C9
Sennoside A	ADH
Sennoside A	ADK
Sennoside A	PAPSS1
Sennoside A	JAK2
Sennoside A	PGF
Sennoside A	HAGH
Sennoside A	PTPN11
Sennoside A	ADAM17
Sennoside A	MMP9
Sennoside A	ESR2
Sennoside A	C8G
Sennoside A	PDK2

Sennoside A	ARF4
Sennoside A	JAK3
Sennoside A	NR3C1
Sennoside A	FABP5
Sennoside A	CTSS
Sennoside A	RHOA
Sennoside A	NOS2
Sennoside A	ZAP70
Sennoside A	THRA
Sennoside A	FABP3
Sennoside A	MMP2
Sennoside A	MAPK12
Sennoside A	MMP1
Sennoside A	FABP6
Sennoside A	LGALS3
Sennoside A	FECH
Sennoside A	FDPS
Sennoside A	PTK2
Sennoside A	ARHGAP1
Sennoside A	IL2
Sennoside A	RAF1
Sennoside A	SETD7
Sennoside A	CCL5
Sennoside A	CDC42
Sennoside A	HINT1
Sennoside A	TEK
Sennoside A	GSTM1
Sennoside A	MME
Sennoside A	NR1I3
Sennoside A	RAB5A
Sennoside A	PRKCQ
Sennoside A	LYZ
Sennoside A	UMPS
Sennoside A	PADI4
Sennoside A	ELANE
Sennoside A	SULT2B1
Sennoside A	GP1BA
Sennoside A	GART
Sennoside A	HEXB
Sennoside A	CTNNA1
Sennoside A	TPH1
Sennoside A	SSE1
Sennoside A	PPARA
Sennoside A	MDM2
Sennoside A	SELE
Sennoside A	SRM
Sennoside A	HPGDS
Sennoside A	EPHX2

Sennoside A	BIRC7
Sennoside A	NR1H4
Sennoside A	GSTA3
Sennoside A	S100A9
Sennoside A	ACAT1
Sennoside A	GCDH
Sennoside A	CSK
Sennoside A	BHMT
Sennoside A	AGXT
Sennoside A	KAT2B
Sennoside A	HRAS
Sennoside A	AMY1A
Sennoside A	AMY1B
Sennoside A	AMY1C
Sennoside A	CALM1
Sennoside A	CALM2
Sennoside A	CALM3
Sennoside A	GNPDA2
Sennoside A	GNPDA1
Rhein	CFB
Rhein	MAPK8
Rhein	MAPK10
Rhein	MAPK14
Rhein	MMP3
Rhein	PPIA
Rhein	CYP19A1
Rhein	AURKA
Rhein	CHEK1
Rhein	HSD17B11
Rhein	BCHE
Rhein	CES1
Rhein	CA2
Rhein	BACE1
Rhein	APOA2
Rhein	ALB
Rhein	SELP
Rhein	EGFR
Rhein	STS
Rhein	HSP90AA1
Rhein	FGFR1
Rhein	AKR1C3
Rhein	PDPK1
Rhein	F2
Rhein	FKBP1A
Rhein	CMA1
Rhein	AR
Rhein	MAOB
Rhein	PGR

Rhein	QPCT
Rhein	ESR1
Rhein	PDE4D
Rhein	ANG
Rhein	PIM1
Rhein	LCN2
Rhein	SHBG
Rhein	PPARD
Rhein	FNTA
Rhein	TYMP
Rhein	SULT2A1
Rhein	F10
Rhein	GSR
Rhein	ADAM17
Rhein	TTR
Rhein	EPHB4
Rhein	NOS3
Rhein	PDE4B
Rhein	SRC
Rhein	MIF
Rhein	CTNNA1
Rhein	CDK2
Rhein	ESRRG
Rhein	PNP
Rhein	IGF1R
Rhein	PTPN1
Rhein	B3GAT1
Rhein	PPARG
Rhein	MMP8
Rhein	PRKACA
Rhein	CTSB
Rhein	AKT1
Rhein	BRAF
Rhein	CFD
Rhein	CSNK1G2
Rhein	PPP5C
Rhein	DPP4
Rhein	PLK1
Rhein	CHIT1
Rhein	HSD11B1
Rhein	KIF11
Rhein	NR3C2
Rhein	YARS
Rhein	HSPA8
Rhein	HSD17B1
Rhein	DAPK1
Rhein	METAP2
Rhein	CCNA2

Rhein	AZGP1
Rhein	GC
Rhein	ISG20
Rhein	CBR1
Rhein	PDE3B
Rhein	MMP12
Rhein	PLA2G2A
Rhein	GSK3B
Rhein	ITK
Rhein	SOD2
Rhein	RHOA
Rhein	PTPN11
Rhein	RAC2
Rhein	PLAU
Rhein	F7
Rhein	PSPH
Rhein	LSS
Rhein	RXRA
Rhein	RHEB
Rhein	SORD
Rhein	CYP2C9
Rhein	AKR1B1
Rhein	AMY2A
Rhein	HPN
Rhein	ADH
Rhein	LCK
Rhein	KDR
Rhein	DHFR
Rhein	TYMS
Rhein	DCK
Rhein	IMPDH2
Rhein	NQO1
Rhein	ATOX1
Rhein	DTYMK
Rhein	MMP7
Rhein	HDAC8
Rhein	CASP7
Rhein	CDK6
Rhein	PAK7
Rhein	JAK3
Rhein	GBA
Rhein	PARP1
Rhein	MTAP
Rhein	PDE5A
Rhein	ARHGAP1
Rhein	ABO
Rhein	GSTA1
Rhein	ACE2

Rhein	ESR2
Rhein	TNK2
Rhein	THRB
Rhein	CTSK
Rhein	PAH
Rhein	AMD1
Rhein	TPI1
Rhein	JAK2
Rhein	NR1H2
Rhein	SEC14L2
Rhein	XIAP
Rhein	CDA
Rhein	BST1
Rhein	HEXB
Rhein	AHCY
Rhein	CAT
Rhein	DHODH
Rhein	HPGDS
Rhein	RAB11A
Rhein	RNASE3
Rhein	SYK
Rhein	CPB1
Rhein	TGFBR1
Rhein	PPARA
Rhein	CASP3
Rhein	ARSA
Rhein	NR1H3
Rhein	UMPS
Rhein	MMP13
Rhein	PCK1
Rhein	PGF
Rhein	NR3C1
Rhein	PDK2
Rhein	ALDOA
Rhein	LDHB
Rhein	NOS2
Rhein	IL2
Rhein	LGALS2
Rhein	BLVRB
Rhein	TGM3
Rhein	SSE1
Rhein	PAK6
Rhein	AKR1C1
Rhein	MET
Rhein	ZAP70
Rhein	GSTP1
Rhein	KAT2B
Rhein	PIK3CG

Rhein	MAPKAPK2
Rhein	SDS
Rhein	NR1I2
Rhein	IGF1
Rhein	SERPINA1
Rhein	PYGL
Rhein	CBS
Rhein	CD1A
Rhein	PLEKHA4
Rhein	MMP9
Rhein	GSTT2
Rhein	FABP3
Rhein	THRA
Rhein	EPHX2
Rhein	CTSF
Rhein	GCK
Rhein	AKR1C2
Rhein	PRKCQ
Rhein	RAB5A
Rhein	ACPP
Rhein	HCK
Rhein	UCK2
Rhein	ADK
Rhein	SULT2B1
Rhein	TEK
Rhein	AKT2
Rhein	ABL1
Rhein	ELANE
Rhein	CLK1
Rhein	APRT
Rhein	C1S
Rhein	GPI
Rhein	F11
Rhein	FDPS
Rhein	MMP2
Rhein	BHMT
Rhein	CTSS
Rhein	DUT
Rhein	PPCDC
Rhein	ACADM
Rhein	CSK
Rhein	S100A9
Rhein	CDK5R1
Rhein	HNF4G
Rhein	CCL5
Rhein	SULT1E1
Rhein	TK1
Rhein	MME

Rhein	HAGH
Rhein	TPH1
Rhein	GCDH
Rhein	CDC42
Rhein	BIRC7
Rhein	NNT
Rhein	ALAD
Rhein	IMPDH1
Rhein	CTSG
Rhein	REN
Rhein	HRAS
Rhein	HMGCR
Rhein	VDR
Rhein	MMP16
Rhein	NMNAT1
Rhein	DPEP1
Rhein	NR1H4
Rhein	LGALS3
Rhein	MAN1B1
Rhein	FECH
Rhein	OTC
Rhein	HADH
Rhein	CDK7
Rhein	GP1BA
Rhein	HINT1
Rhein	PAPSS1
Rhein	NT5M
Rhein	LYZ
Rhein	HPRT1
Rhein	GSTO1
Rhein	TRAPPC3
Rhein	RARG
Rhein	RBP4
Rhein	APAF1
Rhein	EIF4E
Rhein	PTK2
Rhein	ARF4
Rhein	SETD7
Rhein	MAP2K1
Rhein	GART
Rhein	BCAT2
Rhein	MMP1
Rhein	PCMT1
Rhein	GRB2
Rhein	PPP1CC
Rhein	SELE
Rhein	GSTA3
Rhein	OAT

Rhein	TGFB2
Rhein	GSTM2
Rhein	RARA
Rhein	ATIC
Rhein	RNASE2
Rhein	GALE
Rhein	FOLH1
Rhein	RARB
Rhein	RAF1
Rhein	STAT1
Rhein	ITPKA
Rhein	GSTM1
Rhein	FABP7
Rhein	LTA4H
Rhein	BTK
Rhein	AGXT
Rhein	ADAM33
Rhein	ARL5A
Rhein	RAN
Rhein	KIT
Rhein	AMY1A
Rhein	AMY1B
Rhein	AMY1C
Rhein	GNPDA2
Rhein	GNPDA1
Rhein	RAB9B
Rhein	RAB9A
Physcion	MMP3
Physcion	CDK5R1
Physcion	MAPK8
Physcion	MAPK14
Physcion	MAPK10
Physcion	CES1
Physcion	CFB
Physcion	ESR1
Physcion	BCHE
Physcion	CA2
Physcion	GSTP1
Physcion	MAOB
Physcion	PIM1
Physcion	FGFR1
Physcion	CMA1
Physcion	EGFR
Physcion	BACE1
Physcion	CHEK1
Physcion	F2
Physcion	CTSB
Physcion	ANXA5

Physcion	DUSP6
Physcion	AKR1B1
Physcion	KIF11
Physcion	PPARD
Physcion	TTR
Physcion	CCNA2
Physcion	MIF
Physcion	ANG
Physcion	RORA
Physcion	GSK3B
Physcion	F10
Physcion	CDK2
Physcion	HSPA8
Physcion	HSP90AA1
Physcion	BCAT2
Physcion	ADAM17
Physcion	METAP2
Physcion	NOS3
Physcion	PPARG
Physcion	TYMS
Physcion	ESR2
Physcion	CDK6
Physcion	AR
Physcion	PDPK1
Physcion	AKT1
Physcion	AURKA
Physcion	HPN
Physcion	PGR
Physcion	SHBG
Physcion	AKR1C3
Physcion	HMGCR
Physcion	CBR1
Physcion	HSD11B1
Physcion	EPHX2
Physcion	DAPK1
Physcion	PLAU
Physcion	ALB
Physcion	MTAP
Physcion	RNASEL
Physcion	AKR1C1
Physcion	PTPN11
Physcion	FGFR2
Physcion	SOD2
Physcion	PNMT
Physcion	DCK
Physcion	SEC14L2
Physcion	ISG20
Physcion	PAK7

Physcion	MET
Physcion	CDA
Physcion	HSD17B1
Physcion	MMP8
Physcion	RXRA
Physcion	TGFBR1
Physcion	PAH
Physcion	HDAC8
Physcion	PDE4B
Physcion	LSS
Physcion	PTPN1
Physcion	ATOX1
Physcion	CASP3
Physcion	PDE4D
Physcion	ITK
Physcion	ADH1C
Physcion	LTA4H
Physcion	EPHB4
Physcion	SYK
Physcion	SRC
Physcion	AMD1
Physcion	HEXB
Physcion	KDR
Physcion	CTSK
Physcion	LGALS2
Physcion	DPP4
Physcion	PCK1
Physcion	MMP13
Physcion	RHOA
Physcion	CYP2C9
Physcion	DHFR
Physcion	THRB
Physcion	SULT2A1
Physcion	AKR1C2
Physcion	DHODH
Physcion	SERPINA1
Physcion	LCK
Physcion	RNASE3
Physcion	NR3C2
Physcion	PRKACA
Physcion	REN
Physcion	PDE3B
Physcion	TPI1
Physcion	TRAPPC3
Physcion	WAS
Physcion	ELANE
Physcion	ERBB4
Physcion	ACPP

Physcion	CTNNA1
Physcion	BLVRB
Physcion	ADH
Physcion	ABO
Physcion	BIRC7
Physcion	PYGL
Physcion	UCK2
Physcion	BST1
Physcion	JAK3
Physcion	PDE5A
Physcion	BRAF
Physcion	MME
Physcion	HPGDS
Physcion	FABP4
Physcion	AHCY
Physcion	RHEB
Physcion	PPARA
Physcion	CCL5
Physcion	NR1H4
Physcion	YARS
Physcion	NR1H2
Physcion	FECH
Physcion	NQO1
Physcion	PAK6
Physcion	CD1A
Physcion	CSK
Physcion	FKBP1A
Physcion	IGF1
Physcion	FABP6
Physcion	RAB11A
Physcion	NOS2
Physcion	MAPKAPK2
Physcion	NR1I2
Physcion	HAGH
Physcion	MTHFD1
Physcion	MMP12
Physcion	IL2
Physcion	UMPS
Physcion	PITPNA
Physcion	MAP2K1
Physcion	CLK1
Physcion	LGALS3
Physcion	HCK
Physcion	SETD7
Physcion	JAK2
Physcion	HNF4G
Physcion	NR1I3
Physcion	ABL1

Physcion	ZAP70
Physcion	GSTT2
Physcion	CTSS
Physcion	MDM2
Physcion	CRABP2
Physcion	PNP
Physcion	EPHA2
Physcion	SULT1E1
Physcion	F11
Physcion	MMP2
Physcion	AMY2A
Physcion	PLEKHA4
Physcion	SULT2B1
Physcion	MAPK1
Physcion	ESRRG
Physcion	TGM3
Physcion	GPI
Physcion	XIAP
Physcion	NR3C1
Physcion	TK1
Physcion	SDS
Physcion	PRKCQ
Physcion	DTYMK
Physcion	MAN1B1
Physcion	RBP4
Physcion	DPEP1
Physcion	OAT
Physcion	MMP1
Physcion	STAT1
Physcion	THRA
Physcion	BHMT
Physcion	HNMT
Physcion	ARG2
Physcion	CBS
Physcion	VDR
Physcion	PADI4
Physcion	RARA
Physcion	FDPS
Physcion	GSTA1
Physcion	C1S
Physcion	KIT
Physcion	OTC
Physcion	FABP7
Physcion	RAB5A
Physcion	TGFB2
Physcion	GSR
Physcion	PLA2G2A
Physcion	CYP2C8

Physcion	ACADM
Physcion	PKLR
Physcion	NR1H3
Physcion	NT5M
Physcion	TPH1
Physcion	SHMT1
Physcion	EIF4E
Physcion	GCK
Physcion	PIK3R1
Physcion	ARHGAP1
Physcion	GP1BA
Physcion	ALAD
Physcion	RARG
Physcion	DUT
Physcion	RNASE2
Physcion	HMOX1
Physcion	GSTA3
Physcion	PPP1CC
Physcion	ADK
Physcion	GSTM1
Physcion	FGG
Physcion	TEK
Physcion	WARS
Physcion	TPSB2
Physcion	MMP9
Physcion	HINT1
Physcion	NMNAT1
Physcion	KAT2B
Physcion	IMPDH2
Physcion	ITPKA
Physcion	LYZ
Physcion	ATIC
Physcion	LCN2
Physcion	NDST1
Physcion	HPRT1
Physcion	BTK
Physcion	GSTM2
Physcion	APAF1
Physcion	CASP1
Physcion	ADAM33
Physcion	CHIT1
Physcion	HRAS
Physcion	FKBP3
Physcion	INSR
Physcion	HADH
Physcion	CDC42
Physcion	FKBP1B
Physcion	GSTZ1

Physcion	TAP1
Physcion	RXRB
Physcion	GMPR2
Physcion	EEA1
Physcion	FABP3
Physcion	DCXR
Physcion	ARL5B
Physcion	PCTP
Physcion	DDX39B
Physcion	DCPS
Physcion	TTPA
Physcion	GLO1
Physcion	RFK
Physcion	RARB
Physcion	RAC1
Physcion	F7
Physcion	RAN
Physcion	SULT1A1
Physcion	SELE
Physcion	GART
Physcion	UAP1
Physcion	RAP2A
Physcion	DOT1L
Physcion	ARL5A
Physcion	RND3
Physcion	GMPR
Physcion	AMY1A
Physcion	AMY1B
Physcion	AMY1C
Physcion	GNPDA2
Physcion	GNPDA1
Physcion	RAB9B
Physcion	RAB9A
Palmidin A	MMP3
Palmidin A	CES1
Palmidin A	TTR
Palmidin A	MAPK10
Palmidin A	HCK
Palmidin A	BACE1
Palmidin A	PPIA
Palmidin A	FKBP1A
Palmidin A	AKR1C2
Palmidin A	STS
Palmidin A	BMP2
Palmidin A	CASP3
Palmidin A	MAPK1
Palmidin A	MMP13
Palmidin A	GSTP1

Palmidin A	PIM1
Palmidin A	CA1
Palmidin A	GBA
Palmidin A	KIF11
Palmidin A	MAPK8
Palmidin A	SELP
Palmidin A	MAPK14
Palmidin A	TREM1
Palmidin A	NQO2
Palmidin A	BCHE
Palmidin A	CA2
Palmidin A	ALB
Palmidin A	KDR
Palmidin A	HSD17B11
Palmidin A	MAPKAPK2
Palmidin A	NR1H2
Palmidin A	CTSV
Palmidin A	APOA2
Palmidin A	CTSD
Palmidin A	CFB
Palmidin A	CHEK1
Palmidin A	MAOB
Palmidin A	CASP7
Palmidin A	AR
Palmidin A	MTAP
Palmidin A	AURKA
Palmidin A	FAP
Palmidin A	THRB
Palmidin A	HSP90AA1
Palmidin A	CMA1
Palmidin A	METAP1
Palmidin A	GC
Palmidin A	F2
Palmidin A	AKR1B1
Palmidin A	ESR1
Palmidin A	EPHB4
Palmidin A	ADAM17
Palmidin A	ESRRG
Palmidin A	HSD17B1
Palmidin A	CYP19A1
Palmidin A	SHBG
Palmidin A	CDK2
Palmidin A	CLPP
Palmidin A	PNP
Palmidin A	GSR
Palmidin A	SORD
Palmidin A	ICAM2
Palmidin A	PGR

Palmidin A	PPP5C
Palmidin A	ANXA5
Palmidin A	SULT2A1
Palmidin A	CCNA2
Palmidin A	PDPK1
Palmidin A	F10
Palmidin A	DHFR
Palmidin A	PDE4B
Palmidin A	MMP8
Palmidin A	METAP2
Palmidin A	HSPA8
Palmidin A	SRC
Palmidin A	CTSB
Palmidin A	PPARG
Palmidin A	FNTA
Palmidin A	RXRA
Palmidin A	NOS3
Palmidin A	DHODH
Palmidin A	ADH1B
Palmidin A	EGFR
Palmidin A	ADH
Palmidin A	TYMS
Palmidin A	TGFBR1
Palmidin A	BRAF
Palmidin A	TTPA
Palmidin A	PDE4D
Palmidin A	ANG
Palmidin A	AMD1
Palmidin A	ESR2
Palmidin A	CDK6
Palmidin A	CSNK1G2
Palmidin A	FABP4
Palmidin A	FGFR1
Palmidin A	CFD
Palmidin A	PLA2G10
Palmidin A	LCK
Palmidin A	DPP4
Palmidin A	NR3C2
Palmidin A	BMP7
Palmidin A	IGF1R
Palmidin A	GSK3B
Palmidin A	PPARD
Palmidin A	HSD11B1
Palmidin A	AKR1C1
Palmidin A	NQO1
Palmidin A	RORA
Palmidin A	PTPN1
Palmidin A	PNMT

Palmidin A	PRKACA
Palmidin A	REN
Palmidin A	TNNC1
Palmidin A	PLK1
Palmidin A	SOD2
Palmidin A	MIF
Palmidin A	ITK
Palmidin A	JAK3
Palmidin A	ADH1C
Palmidin A	GSTA1
Palmidin A	DCK
Palmidin A	ADK
Palmidin A	DUSP6
Palmidin A	MDM2
Palmidin A	SULT1E1
Palmidin A	AKR1C3
Palmidin A	HDAC8
Palmidin A	PIK3CG
Palmidin A	PLA2G2A
Palmidin A	MET
Palmidin A	PGF
Palmidin A	CTNNA1
Palmidin A	SYK
Palmidin A	PAH
Palmidin A	HPN
Palmidin A	PDHB
Palmidin A	CTSS
Palmidin A	CDA
Palmidin A	PDE5A
Palmidin A	SULT2B1
Palmidin A	CSNK2A1
Palmidin A	WAS
Palmidin A	MMP12
Palmidin A	EPHX2
Palmidin A	NR1H4
Palmidin A	ABO
Palmidin A	ATOX1
Palmidin A	AMY2A
Palmidin A	F7
Palmidin A	TNK2
Palmidin A	HMGCR
Palmidin A	YARS
Palmidin A	LSS
Palmidin A	AHCY
Palmidin A	ALDH2
Palmidin A	FABP5
Palmidin A	XIAP
Palmidin A	NOS2

Palmidin A	AKT1
Palmidin A	FGFR2
Palmidin A	FABP7
Palmidin A	ISG20
Palmidin A	IMPDH2
Palmidin A	CBR1
Palmidin A	PTPN11
Palmidin A	PAK7
Palmidin A	SEC14L2
Palmidin A	MTHFD1
Palmidin A	PARP1
Palmidin A	REG1A
Palmidin A	HK1
Palmidin A	NR1I3
Palmidin A	RNASEL
Palmidin A	NCS1
Palmidin A	PDE3B
Palmidin A	JAK2
Palmidin A	CTSK
Palmidin A	BCAT2
Palmidin A	PDK2
Palmidin A	BST1
Palmidin A	LGALS7
Palmidin A	PLAU
Palmidin A	NR1I2
Palmidin A	FABP3
Palmidin A	HADH
Palmidin A	THRA
Palmidin A	ZAP70
Palmidin A	FABP6
Palmidin A	PYGL
Palmidin A	UCK2
Palmidin A	ELANE
Palmidin A	NR3C1
Palmidin A	PRKCQ
Palmidin A	RAC2
Palmidin A	CYP2C9
Palmidin A	PNPO
Palmidin A	RHOA
Palmidin A	HPGDS
Palmidin A	ITGAL
Palmidin A	RBP4
Palmidin A	CRABP2
Palmidin A	LGALS2
Palmidin A	MAN1B1
Palmidin A	S100A9
Palmidin A	BIRC7
Palmidin A	PPARA

Palmidin A	PCK1
Palmidin A	IGF1
Palmidin A	SERPINA1
Palmidin A	DTYMK
Palmidin A	ACPP
Palmidin A	ALDOA
Palmidin A	TPI1
Palmidin A	ERRA
Palmidin A	HEXB
Palmidin A	BLVRB
Palmidin A	ARSA
Palmidin A	LCN2
Palmidin A	CSK
Palmidin A	CTSF
Palmidin A	NR1H3
Palmidin A	MAP2K1
Palmidin A	GALE
Palmidin A	RARG
Palmidin A	TGM3
Palmidin A	PAK6
Palmidin A	RNASE3
Palmidin A	B3GAT1
Palmidin A	ERBB4
Palmidin A	IMPA1
Palmidin A	LYZ
Palmidin A	GM2A
Palmidin A	KAT2B
Palmidin A	CCL5
Palmidin A	TRAPPC3
Palmidin A	ARHGAP1
Palmidin A	TPH1
Palmidin A	IL2
Palmidin A	CCNT1
Palmidin A	MMP9
Palmidin A	STAT1
Palmidin A	SHMT1
Palmidin A	MMP2
Palmidin A	GSTA3
Palmidin A	RHEB
Palmidin A	DPEP1
Palmidin A	UMPS
Palmidin A	FECH
Palmidin A	GPI
Palmidin A	PSAP
Palmidin A	TEK
Palmidin A	TGFB2
Palmidin A	EIF4E
Palmidin A	RAB11A

Palmidin A	CASP1
Palmidin A	CDK5R1
Palmidin A	SRM
Palmidin A	HNF4G
Palmidin A	SDS
Palmidin A	GSTT2
Palmidin A	MME
Palmidin A	VDR
Palmidin A	CTSG
Palmidin A	IMPDH1
Palmidin A	CLK1
Palmidin A	ACADM
Palmidin A	RARB
Palmidin A	APRT
Palmidin A	EPHA2
Palmidin A	NMNAT1
Palmidin A	ARG2
Palmidin A	ABL1
Palmidin A	AKT2
Palmidin A	PPP1CC
Palmidin A	ST14
Palmidin A	CHIT1
Palmidin A	LGALS3
Palmidin A	AGXT
Palmidin A	PLEKHA4
Palmidin A	CBS
Palmidin A	CDK7
Palmidin A	HAGH
Palmidin A	PPCDC
Palmidin A	KYAT1
Palmidin A	PAPSS1
Palmidin A	ACE
Palmidin A	MMP1
Palmidin A	C1S
Palmidin A	AMY1A
Palmidin A	AMY1B
Palmidin A	AMY1C
Palmidin A	CALM1
Palmidin A	CALM2
Palmidin A	CALM3
Palmidin A	GNPDA2
Palmidin A	GNPDA1
Mutatochrome	ALB
Mutatochrome	STS
Mutatochrome	MAPK1
Mutatochrome	MMP13
Mutatochrome	MAPKAPK2
Mutatochrome	FKBP1A

Mutatochrome	KIF11
Mutatochrome	PIM1
Mutatochrome	APOA2
Mutatochrome	BMP2
Mutatochrome	AKR1C2
Mutatochrome	TTR
Mutatochrome	THRB
Mutatochrome	CASP7
Mutatochrome	CA2
Mutatochrome	MAOB
Mutatochrome	GC
Mutatochrome	ITGAL
Mutatochrome	CES1
Mutatochrome	F10
Mutatochrome	PIK3CG
Mutatochrome	MAPK10
Mutatochrome	SEC14L2
Mutatochrome	CDK2
Mutatochrome	MAPK14
Mutatochrome	CFB
Mutatochrome	PPIA
Mutatochrome	F2
Mutatochrome	RORA
Mutatochrome	AR
Mutatochrome	MAPK8
Mutatochrome	NR1H4
Mutatochrome	CDK5R1
Mutatochrome	PPARG
Mutatochrome	ADAM17
Mutatochrome	CASP3
Mutatochrome	MMP3
Mutatochrome	PGR
Mutatochrome	RBP4
Mutatochrome	AKR1B1
Mutatochrome	EGFR
Mutatochrome	DPP4
Mutatochrome	ERRA
Mutatochrome	PTPN11
Mutatochrome	HSD17B1
Mutatochrome	ANXA5
Mutatochrome	METAP2
Mutatochrome	PRKACA
Mutatochrome	WAS
Mutatochrome	SULT2A1
Mutatochrome	NR3C2
Mutatochrome	EPHB4
Mutatochrome	AKR1C3
Mutatochrome	GSTA1

Mutatochrome	CYP19A1
Mutatochrome	TRAPPC3
Mutatochrome	KDR
Mutatochrome	GSTP1
Mutatochrome	HSD11B1
Mutatochrome	SRC
Mutatochrome	PDE4B
Mutatochrome	PNMT
Mutatochrome	HPGDS
Mutatochrome	TGFBR1
Mutatochrome	ESR1
Mutatochrome	BCHE
Mutatochrome	DHODH
Mutatochrome	BRAF
Mutatochrome	PDPK1
Mutatochrome	SHBG
Mutatochrome	DUSP6
Mutatochrome	TNNC1
Mutatochrome	RXRA
Mutatochrome	PPP5C
Mutatochrome	ESRRG
Mutatochrome	FABP4
Mutatochrome	PTPN1
Mutatochrome	BACE1
Mutatochrome	NQO1
Mutatochrome	PDE4D
Mutatochrome	LCK
Mutatochrome	TTPA
Mutatochrome	IGF1R
Mutatochrome	PDK2
Mutatochrome	PPARD
Mutatochrome	CFD
Mutatochrome	CHEK1
Mutatochrome	MMP8
Mutatochrome	MIF
Mutatochrome	HSP90AA1
Mutatochrome	PLA2G2A
Mutatochrome	AKR1C1
Mutatochrome	TYMS
Mutatochrome	BLVRB
Mutatochrome	CCNA2
Mutatochrome	HDAC8
Mutatochrome	PLK1
Mutatochrome	PLA2G10
Mutatochrome	CTNNA1
Mutatochrome	MET
Mutatochrome	SYK
Mutatochrome	PAK7

Mutatochrome	NOS3
Mutatochrome	FABP7
Mutatochrome	ADH
Mutatochrome	MDM2
Mutatochrome	GSR
Mutatochrome	FABP3
Mutatochrome	NR1H3
Mutatochrome	FNTA
Mutatochrome	FABP6
Mutatochrome	HSPA8
Mutatochrome	ADK
Mutatochrome	CYP2C8
Mutatochrome	SULT2B1
Mutatochrome	NR1H2
Mutatochrome	ERBB4
Mutatochrome	RARG
Mutatochrome	REN
Mutatochrome	PPARA
Mutatochrome	MMP2
Mutatochrome	ELANE
Mutatochrome	CRABP2
Mutatochrome	NR1I3
Mutatochrome	GM2A
Mutatochrome	FGFR1
Mutatochrome	SULT1E1
Mutatochrome	PARP1
Mutatochrome	SERPINA1
Mutatochrome	LSS
Mutatochrome	ESR2
Mutatochrome	JAK3
Mutatochrome	ITK
Mutatochrome	ADH1C
Mutatochrome	ABL1
Mutatochrome	EPHX2
Mutatochrome	IGF1
Mutatochrome	GSK3B
Mutatochrome	HNMT
Mutatochrome	LCN2
Mutatochrome	FGFR2
Mutatochrome	PDE5A
Mutatochrome	PPP1CC
Mutatochrome	HNF4G
Mutatochrome	RARB
Mutatochrome	FABP5
Mutatochrome	PGF
Mutatochrome	SORD
Mutatochrome	SOD2
Mutatochrome	HMGCR

Mutatochrome	MMP12
Mutatochrome	F7
Mutatochrome	ALDH2
Mutatochrome	MTAP
Mutatochrome	CDK6
Mutatochrome	PDE3B
Mutatochrome	NQO2
Mutatochrome	MAP2K1
Mutatochrome	AURKA
Mutatochrome	IL2
Mutatochrome	CYP2C9
Mutatochrome	CBR1
Mutatochrome	S100A9
Mutatochrome	YARS
Mutatochrome	RARA
Mutatochrome	PSAP
Mutatochrome	NR3C1
Mutatochrome	CTSK
Mutatochrome	RXRB
Mutatochrome	FECH
Mutatochrome	ZAP70
Mutatochrome	DCK
Mutatochrome	PRKCQ
Mutatochrome	THRA
Mutatochrome	GCK
Mutatochrome	NR1I2
Mutatochrome	PLAT
Mutatochrome	CCNT1
Mutatochrome	BPI
Mutatochrome	DPEP1
Mutatochrome	ABO
Mutatochrome	TEK
Mutatochrome	PADI4
Mutatochrome	ACPP
Mutatochrome	HPN
Mutatochrome	GRB2
Mutatochrome	VDR
Mutatochrome	AKT2
Mutatochrome	CSK
Mutatochrome	SHMT1
Mutatochrome	MTHFD1
Mutatochrome	PYGL
Mutatochrome	GLO1
Mutatochrome	BIRC7
Mutatochrome	PCTP
Mutatochrome	CTSB
Mutatochrome	HSP90AB1
Mutatochrome	PIK3R1

Mutatochrome	HMOX1
Mutatochrome	TGM3
Mutatochrome	KIT
Mutatochrome	CD1A
Mutatochrome	BCL2L1
Mutatochrome	HCK
Mutatochrome	JAK2
Mutatochrome	CTSS
Mutatochrome	CTSG
Mutatochrome	CTSF
Mutatochrome	XIAP
Mutatochrome	DHFR
Mutatochrome	LTA4H
Mutatochrome	ACADM
Mutatochrome	AGXT
Mutatochrome	SETD7
Mutatochrome	ARG1
Mutatochrome	PCK1
Mutatochrome	TGFB2
Mutatochrome	SDS
Mutatochrome	CBS
Mutatochrome	CASP1
Mutatochrome	MME
Mutatochrome	PROCR
Mutatochrome	CMA1
Mutatochrome	BHMT
Mutatochrome	EIF4E
Mutatochrome	CHIT1
Mutatochrome	F11
Mutatochrome	AMD1
Mutatochrome	BCAT2
Mutatochrome	ADAMTS4
Mutatochrome	LGALS3
Mutatochrome	MMP9
Mutatochrome	PITPNA
Mutatochrome	INSR
Mutatochrome	FKBP1B
Mutatochrome	FOLH1
Mutatochrome	OAT
Mutatochrome	ADAM33
Mutatochrome	FKBP3
Mutatochrome	TPSB2
Mutatochrome	GART
Mutatochrome	GSTT2
Mutatochrome	MAOA
Mutatochrome	IVD
Mutatochrome	RFK
Mutatochrome	AMY1A

Mutatochrome	AMY1B
Mutatochrome	AMY1C
Mutatochrome	CALM1
Mutatochrome	CALM2
Mutatochrome	CALM3
Eupatin	BCHE
Eupatin	F2
Eupatin	GBA
Eupatin	ESR1
Eupatin	PGR
Eupatin	EGFR
Eupatin	MAPK10
Eupatin	FAP
Eupatin	HSPA8
Eupatin	CCNA2
Eupatin	CTSV
Eupatin	CA2
Eupatin	TTR
Eupatin	PDE4B
Eupatin	BACE1
Eupatin	CA12
Eupatin	MAPK8
Eupatin	PIM1
Eupatin	CDK2
Eupatin	DDX6
Eupatin	PNP
Eupatin	GSTP1
Eupatin	PPIA
Eupatin	CA1
Eupatin	ESR2
Eupatin	CFB
Eupatin	TREM1
Eupatin	MMP3
Eupatin	NR1H2
Eupatin	ANG
Eupatin	AURKA
Eupatin	CTSD
Eupatin	AR
Eupatin	GSR
Eupatin	CYP19A1
Eupatin	CDK6
Eupatin	CHEK1
Eupatin	CDK5R1
Eupatin	PDE4D
Eupatin	PAH
Eupatin	BCAT2
Eupatin	SRC
Eupatin	HSP90AA1

Eupatin	SHBG
Eupatin	LSS
Eupatin	AKR1B1
Eupatin	ISG20
Eupatin	CFD
Eupatin	CSNK1G2
Eupatin	METAP1
Eupatin	IMPDH2
Eupatin	MAOB
Eupatin	SORD
Eupatin	CBR1
Eupatin	MTAP
Eupatin	TYMP
Eupatin	MAPK14
Eupatin	PLA2G10
Eupatin	C1R
Eupatin	PDPK1
Eupatin	EPHB4
Eupatin	PLAU
Eupatin	HSD17B1
Eupatin	NPR3
Eupatin	CTSB
Eupatin	MMP13
Eupatin	PYGL
Eupatin	PLK1
Eupatin	ESRRG
Eupatin	CASP7
Eupatin	ADAM17
Eupatin	DHFR
Eupatin	RXRA
Eupatin	RAC2
Eupatin	HK1
Eupatin	HCK
Eupatin	F10
Eupatin	APCS
Eupatin	ABO
Eupatin	FNTA
Eupatin	NQO1
Eupatin	KDR
Eupatin	MAN1B1
Eupatin	AKR1C1
Eupatin	GSK3B
Eupatin	NR3C2
Eupatin	NOS3
Eupatin	HSD11B1
Eupatin	ADK
Eupatin	AHCY
Eupatin	KIF11

Eupatin	REG1A
Eupatin	SOD2
Eupatin	MMP12
Eupatin	PCK1
Eupatin	BMP7
Eupatin	ALB
Eupatin	DPP4
Eupatin	SULT2A1
Eupatin	FABP4
Eupatin	MIF
Eupatin	ATOX1
Eupatin	EPHX2
Eupatin	PDE3B
Eupatin	AMD1
Eupatin	CCNT1
Eupatin	BST1
Eupatin	NQO2
Eupatin	FGFR1
Eupatin	PRKACA
Eupatin	TNK2
Eupatin	PPARD
Eupatin	LGALS7
Eupatin	LCK
Eupatin	AZGP1
Eupatin	ELANE
Eupatin	PTPN1
Eupatin	F7
Eupatin	ADH
Eupatin	RHEB
Eupatin	DAPK1
Eupatin	RHOA
Eupatin	NMNAT1
Eupatin	CES1
Eupatin	ADH1C
Eupatin	MMP8
Eupatin	CTSK
Eupatin	PDHB
Eupatin	IGF1R
Eupatin	PDE5A
Eupatin	CSNK2A1
Eupatin	DHODH
Eupatin	AKR1C3
Eupatin	ME2
Eupatin	IMPA1
Eupatin	SSE1
Eupatin	RAB11A
Eupatin	AKR1C2
Eupatin	METAP2

Eupatin	LDHB
Eupatin	CLK1
Eupatin	GSTT2
Eupatin	CDA
Eupatin	HDAC8
Eupatin	MET
Eupatin	JAK3
Eupatin	RNASE3
Eupatin	B3GAT1
Eupatin	KYAT1
Eupatin	GALE
Eupatin	CCL5
Eupatin	NCS1
Eupatin	BIRC7
Eupatin	REN
Eupatin	ITK
Eupatin	DCK
Eupatin	AMY2A
Eupatin	FGFR2
Eupatin	DTYMK
Eupatin	EPHA2
Eupatin	PDK2
Eupatin	UMPS
Eupatin	APRT
Eupatin	CASP3
Eupatin	TGFBR1
Eupatin	ALDOA
Eupatin	LGALS2
Eupatin	PPARG
Eupatin	RNASEL
Eupatin	AKT1
Eupatin	C1S
Eupatin	NR1I2
Eupatin	CD1A
Eupatin	HEXB
Eupatin	SHMT1
Eupatin	TPI1
Eupatin	HRAS
Eupatin	INSR
Eupatin	BHMT
Eupatin	ARHGAP1
Eupatin	PAK6
Eupatin	TGM3
Eupatin	MAPKAPK2
Eupatin	SERPINA1
Eupatin	ERBB4
Eupatin	SYK
Eupatin	CBS

Eupatin	CYP2C9
Eupatin	PPARA
Eupatin	XIAP
Eupatin	SELP
Eupatin	UCK2
Eupatin	LYZ
Eupatin	ACPP
Eupatin	TYMS
Eupatin	OTC
Eupatin	IL2
Eupatin	NT5M
Eupatin	GSTA3
Eupatin	GPI
Eupatin	CDK7
Eupatin	ACE
Eupatin	GSTA1
Eupatin	NR1H3
Eupatin	NOS2
Eupatin	THRB
Eupatin	GSTZ1
Eupatin	RAB5A
Eupatin	HPGDS
Eupatin	RARA
Eupatin	IMPDH1
Eupatin	FKBP1A
Eupatin	ZAP70
Eupatin	AGXT
Eupatin	FABP6
Eupatin	IGF1
Eupatin	F11
Eupatin	CTSS
Eupatin	PTK2
Eupatin	RBP4
Eupatin	HAGH
Eupatin	SELE
Eupatin	ALDH2
Eupatin	MMP9
Eupatin	SULT1E1
Eupatin	TK1
Eupatin	PAPSS1
Eupatin	ATIC
Eupatin	BRAF
Eupatin	JAK2
Eupatin	CHIT1
Eupatin	HINT1
Eupatin	S100A9
Eupatin	TPH1
Eupatin	ARSA

Eupatin	SDS
Eupatin	SULT2B1
Eupatin	CAT
Eupatin	ARG2
Eupatin	THRA
Eupatin	LGALS3
Eupatin	HPRT1
Eupatin	PLA2G2A
Eupatin	PITPNA
Eupatin	NR3C1
Eupatin	PPCDC
Eupatin	CTSG
Eupatin	FECH
Eupatin	ARG1
Eupatin	AKT2
Eupatin	CTSF
Eupatin	GCK
Eupatin	PIK3R1
Eupatin	MME
Eupatin	EIF4E
Eupatin	GSTM2
Eupatin	CDC42
Eupatin	ARL5A
Eupatin	KIT
Eupatin	SETD7
Eupatin	DPEP1
Eupatin	CD209
Eupatin	FABP3
Eupatin	ACAT1
Eupatin	FDPS
Eupatin	GMPR
Eupatin	NR1H4
Eupatin	PKLR
Eupatin	GSTO1
Eupatin	MAPK1
Eupatin	HMGCR
Eupatin	STAT1
Eupatin	DUT
Eupatin	OAT
Eupatin	MMP1
Eupatin	BTK
Eupatin	CMA1
Eupatin	GART
Eupatin	BLVRB
Eupatin	CLEC4M
Eupatin	TAP1
Eupatin	TEK
Eupatin	GP1BA

Eupatin	VDR
Eupatin	PRKCQ
Eupatin	GMPR2
Eupatin	AMY1A
Eupatin	AMY1B
Eupatin	AMY1C
Eupatin	GNPDA2
Eupatin	GNPDA1
Eupatin	RAB9B
Eupatin	RAB9A
Emodin	MMP3
Emodin	CDK5R1
Emodin	ESR1
Emodin	MAPK8
Emodin	MAPK14
Emodin	MAPK10
Emodin	CES1
Emodin	CFB
Emodin	SELP
Emodin	BCHE
Emodin	CA2
Emodin	GSTP1
Emodin	MAOB
Emodin	PIM1
Emodin	FGFR1
Emodin	EGFR
Emodin	BACE1
Emodin	F2
Emodin	DHFR
Emodin	CTSB
Emodin	DUSP6
Emodin	ANXA5
Emodin	AKR1B1
Emodin	CHEK1
Emodin	KIF11
Emodin	PPARD
Emodin	TTR
Emodin	CCNA2
Emodin	MIF
Emodin	CDK2
Emodin	GSK3B
Emodin	RORA
Emodin	HSPA8
Emodin	ANG
Emodin	F10
Emodin	HSP90AA1
Emodin	ADAM17
Emodin	BCAT2

Emodin	MTAP
Emodin	ESR2
Emodin	METAP2
Emodin	DCK
Emodin	NOS3
Emodin	PPARG
Emodin	PNP
Emodin	TYMS
Emodin	AR
Emodin	PDPK1
Emodin	PGR
Emodin	AKT1
Emodin	AURKA
Emodin	PLK1
Emodin	SOD2
Emodin	HPN
Emodin	SHBG
Emodin	HMGCR
Emodin	CSNK2A1
Emodin	CBR1
Emodin	CTNNA1
Emodin	AKR1C3
Emodin	EPHX2
Emodin	CDK6
Emodin	HSD11B1
Emodin	ALB
Emodin	AKR1C1
Emodin	FGFR2
Emodin	PTPN11
Emodin	ADH
Emodin	PNMT
Emodin	HSD17B1
Emodin	SEC14L2
Emodin	ISG20
Emodin	PAK7
Emodin	MET
Emodin	CDA
Emodin	MMP8
Emodin	PLAU
Emodin	TGFBR1
Emodin	ALDH2
Emodin	PAH
Emodin	HDAC8
Emodin	PDE4B
Emodin	LTA4H
Emodin	PTPN1
Emodin	PDE4D
Emodin	ITK

Emodin	ADH1C
Emodin	CTSK
Emodin	NR1H4
Emodin	PRKACA
Emodin	EPHB4
Emodin	AMD1
Emodin	NQO1
Emodin	LSS
Emodin	HEXB
Emodin	KDR
Emodin	ATOX1
Emodin	LGALS2
Emodin	DPP4
Emodin	TNK2
Emodin	SYK
Emodin	MMP13
Emodin	PCK1
Emodin	ABO
Emodin	THRB
Emodin	SERPINA1
Emodin	CYP2C9
Emodin	RXRA
Emodin	SULT2A1
Emodin	LCK
Emodin	SRC
Emodin	AHCY
Emodin	HPGDS
Emodin	TRAPPC3
Emodin	ELANE
Emodin	BST1
Emodin	ERBB4
Emodin	REN
Emodin	DHODH
Emodin	WAS
Emodin	BIRC7
Emodin	UCK2
Emodin	IL2
Emodin	NR1H2
Emodin	BLVRB
Emodin	FABP4
Emodin	PDE5A
Emodin	PYGL
Emodin	BRAF
Emodin	MME
Emodin	ACPP
Emodin	ALDOA
Emodin	CD1A
Emodin	JAK3

Emodin	PPARA
Emodin	GBA
Emodin	FECH
Emodin	RAB11A
Emodin	IGF1
Emodin	TPH1
Emodin	CSK
Emodin	FKBP1A
Emodin	FABP6
Emodin	NR1I2
Emodin	TPI1
Emodin	KIT
Emodin	MMP12
Emodin	MTHFD1
Emodin	GSTT2
Emodin	MAPKAPK2
Emodin	NOS2
Emodin	NR3C2
Emodin	MAP2K1
Emodin	MAN1B1
Emodin	CLK1
Emodin	NMNAT1
Emodin	HAGH
Emodin	SULT1E1
Emodin	UMPS
Emodin	MMP9
Emodin	PITPNA
Emodin	SETD7
Emodin	HCK
Emodin	RNASE3
Emodin	NR1I3
Emodin	HNF4G
Emodin	CTSS
Emodin	JAK2
Emodin	ACADM
Emodin	F11
Emodin	PAK6
Emodin	MMP2
Emodin	IMPDH2
Emodin	ZAP70
Emodin	MDM2
Emodin	AMY2A
Emodin	GPI
Emodin	CASP3
Emodin	CTSF
Emodin	CCL5
Emodin	ABL1
Emodin	TGM3

Emodin	MAPK1
Emodin	PLEKHA4
Emodin	XIAP
Emodin	OAT
Emodin	VDR
Emodin	PRKCQ
Emodin	DTYMK
Emodin	DPEP1
Emodin	GSR
Emodin	RBP4
Emodin	APRT
Emodin	STAT1
Emodin	SULT2B1
Emodin	CBS
Emodin	NR1H3
Emodin	ESRRG
Emodin	PADI4
Emodin	ARG2
Emodin	HNMT
Emodin	SDS
Emodin	CHIT1
Emodin	THRA
Emodin	FDPS
Emodin	FABP7
Emodin	TGFB2
Emodin	BHMT
Emodin	OTC
Emodin	RARA
Emodin	AKR1C2
Emodin	PTK2
Emodin	NT5M
Emodin	NR3C1
Emodin	C1S
Emodin	GSTA1
Emodin	CYP2C8
Emodin	MMP1
Emodin	EPHA2
Emodin	S100A9
Emodin	PLA2G2A
Emodin	TK1
Emodin	AKT2
Emodin	EIF4E
Emodin	SHMT1
Emodin	PCMT1
Emodin	GCK
Emodin	CRABP2
Emodin	PIK3R1
Emodin	ALAD

Emodin	RARG
Emodin	DUT
Emodin	GSTM2
Emodin	FGG
Emodin	PPP1CC
Emodin	RNASE2
Emodin	TEK
Emodin	GALE
Emodin	GSTM1
Emodin	RHEB
Emodin	HMOX1
Emodin	INSR
Emodin	WARS
Emodin	ADK
Emodin	RAB5A
Emodin	GSTA3
Emodin	KAT2B
Emodin	HINT1
Emodin	HPRT1
Emodin	ITPKA
Emodin	LYZ
Emodin	RAF1
Emodin	LCN2
Emodin	GRB2
Emodin	BTK
Emodin	IMPDH1
Emodin	ATIC
Emodin	EEA1
Emodin	GART
Emodin	APAF1
Emodin	CASP1
Emodin	ADAM33
Emodin	NME2
Emodin	NDST1
Emodin	HADH
Emodin	FKBP3
Emodin	FKBP1B
Emodin	FABP3
Emodin	ARL5A
Emodin	RAP2A
Emodin	DAPK1
Emodin	HRAS
Emodin	SELE
Emodin	RXRB
Emodin	GMPR2
Emodin	RAN
Emodin	MMP16
Emodin	PFKFB1

Emodin	HSP90AB1
Emodin	ARL5B
Emodin	DDX39B
Emodin	PCTP
Emodin	TAP1
Emodin	UAP1
Emodin	TTPA
Emodin	RAC1
Emodin	AMY1A
Emodin	AMY1B
Emodin	AMY1C
Emodin	GNPDA2
Emodin	GNPDA1
Emodin	RAB9B
Emodin	RAB9A
Daucosterol	BMP2
Daucosterol	MAOB
Daucosterol	PIM1
Daucosterol	KIF11
Daucosterol	AKR1C2
Daucosterol	STS
Daucosterol	ALB
Daucosterol	APOA2
Daucosterol	CA2
Daucosterol	CASP7
Daucosterol	MAPK1
Daucosterol	AR
Daucosterol	MAPKAPK2
Daucosterol	THRB
Daucosterol	PPIA
Daucosterol	CYP19A1
Daucosterol	TTR
Daucosterol	CCNA2
Daucosterol	MAPK14
Daucosterol	MMP13
Daucosterol	PPARG
Daucosterol	GC
Daucosterol	RORA
Daucosterol	PGR
Daucosterol	BACE1
Daucosterol	HSD11B1
Daucosterol	ESR1
Daucosterol	PTPN11
Daucosterol	ADAM17
Daucosterol	ANXA5
Daucosterol	SRC
Daucosterol	SEC14L2
Daucosterol	CDK2

Daucosterol	BRAF
Daucosterol	EGFR
Daucosterol	PDPK1
Daucosterol	MAPK8
Daucosterol	PDE4B
Daucosterol	NOS3
Daucosterol	FNTA
Daucosterol	PNMT
Daucosterol	AKR1B1
Daucosterol	PPP5C
Daucosterol	NR1H4
Daucosterol	MAPK10
Daucosterol	ERRA
Daucosterol	TNNC1
Daucosterol	TRAPPC3
Daucosterol	HSD17B1
Daucosterol	ESRRG
Daucosterol	F10
Daucosterol	F2
Daucosterol	SULT2A1
Daucosterol	GSR
Daucosterol	PRKACA
Daucosterol	NR3C2
Daucosterol	GSTP1
Daucosterol	EPHB4
Daucosterol	DPP4
Daucosterol	DHODH
Daucosterol	CA1
Daucosterol	DUSP6
Daucosterol	TGFBR1
Daucosterol	HPGDS
Daucosterol	ADH1B
Daucosterol	WAS
Daucosterol	TTPA
Daucosterol	SHBG
Daucosterol	IGF1R
Daucosterol	ADH
Daucosterol	CES1
Daucosterol	PPARA
Daucosterol	AKR1C1
Daucosterol	PPARD
Daucosterol	ADK
Daucosterol	MDM2
Daucosterol	KDR
Daucosterol	GSTA1
Daucosterol	AKR1C3
Daucosterol	NR1H2
Daucosterol	PLK1

Daucosterol	PTPN1
Daucosterol	HSP90AA1
Daucosterol	ABO
Daucosterol	CHEK1
Daucosterol	REN
Daucosterol	RXRA
Daucosterol	RBP4
Daucosterol	HDAC8
Daucosterol	PDK2
Daucosterol	ADH1C
Daucosterol	GSK3B
Daucosterol	ALDH2
Daucosterol	PARP1
Daucosterol	PDE4D
Daucosterol	METAP2
Daucosterol	FGFR2
Daucosterol	NQO1
Daucosterol	HSPA8
Daucosterol	PGF
Daucosterol	NR1H3
Daucosterol	SULT2B1
Daucosterol	NR1I3
Daucosterol	SORD
Daucosterol	F7
Daucosterol	LSS
Daucosterol	EPHX2
Daucosterol	HNF4G
Daucosterol	FABP6
Daucosterol	CTNNA1
Daucosterol	IL2
Daucosterol	FABP3
Daucosterol	BLVRB
Daucosterol	SYK
Daucosterol	FABP7
Daucosterol	MMP2
Daucosterol	MMP8
Daucosterol	HPN
Daucosterol	BCHE
Daucosterol	CYP2C8
Daucosterol	MMP12
Daucosterol	ITGAL
Daucosterol	PDE3B
Daucosterol	ERBB4
Daucosterol	MET
Daucosterol	YARS
Daucosterol	NQO2
Daucosterol	ESR2
Daucosterol	MAP2K1

Daucosterol	IGF1
Daucosterol	TYMS
Daucosterol	SULT1E1
Daucosterol	CRABP2
Daucosterol	RARG
Daucosterol	HNMT
Daucosterol	SERPINA1
Daucosterol	JAK3
Daucosterol	GM2A
Daucosterol	ITK
Daucosterol	LCK
Daucosterol	PSAP
Daucosterol	NR3C1
Daucosterol	FABP5
Daucosterol	PDE5A
Daucosterol	MMP3
Daucosterol	PLA2G2A
Daucosterol	CASP3
Daucosterol	PIK3CG
Daucosterol	FGFR1
Daucosterol	NR1I2
Daucosterol	ELANE
Daucosterol	CYP2C9
Daucosterol	MTHFD1
Daucosterol	RARB
Daucosterol	FECH
Daucosterol	AKT2
Daucosterol	LCN2
Daucosterol	HSP90AB1
Daucosterol	MTAP
Daucosterol	DPEP1
Daucosterol	S100A9
Daucosterol	RARA
Daucosterol	VDR
Daucosterol	THRA
Daucosterol	ZAP70
Daucosterol	AURKA
Daucosterol	PRKCQ
Daucosterol	HMGCR
Daucosterol	PAK7
Daucosterol	FKBP1A
Daucosterol	ABL1
Daucosterol	TEK
Daucosterol	CTSB
Daucosterol	PPP1CC
Daucosterol	JAK2
Daucosterol	SOD2
Daucosterol	RXRB

Daucosterol	TGM3
Daucosterol	CTSS
Daucosterol	HCK
Daucosterol	ACADM
Daucosterol	GRB2
Daucosterol	HMOX1
Daucosterol	PCTP
Daucosterol	KIT
Daucosterol	DCK
Daucosterol	CMA1
Daucosterol	BIRC7
Daucosterol	MME
Daucosterol	PIK3R1
Daucosterol	GART
Daucosterol	GCK
Daucosterol	AMD1
Daucosterol	CTSK
Daucosterol	EIF4E
Daucosterol	LTA4H
Daucosterol	TGFB2
Daucosterol	BCL2L1
Daucosterol	PROCR
Daucosterol	OAT
Daucosterol	GLO1
Daucosterol	CASP1
Daucosterol	FOLH1
Daucosterol	INSR
Daucosterol	BHMT
Daucosterol	F11
Daucosterol	FKBP1B
Daucosterol	BCAT2
Daucosterol	GSTM1
Daucosterol	IVD
Daucosterol	GSTT2
Daucosterol	MAOA
Daucosterol	RFK
Daucosterol	CALM1
Daucosterol	CALM2
Daucosterol	CALM3
Danthron	MMP3
Danthron	CES1
Danthron	MAPK8
Danthron	MAPK14
Danthron	MAPK10
Danthron	CFB
Danthron	BCHE
Danthron	CA2
Danthron	MAOB

Danthron	FGFR1
Danthron	CTSB
Danthron	PIM1
Danthron	PPARD
Danthron	EGFR
Danthron	ANG
Danthron	F10
Danthron	HSP90AA1
Danthron	F2
Danthron	BCAT2
Danthron	CDK2
Danthron	CHEK1
Danthron	NOS3
Danthron	CBR1
Danthron	EPHX2
Danthron	DCK
Danthron	METAP2
Danthron	PAK7
Danthron	MMP8
Danthron	ISG20
Danthron	PLAU
Danthron	HSD11B1
Danthron	CCNA2
Danthron	ITK
Danthron	EPHB4
Danthron	PRKACA
Danthron	PDE4D
Danthron	HEXB
Danthron	TYMS
Danthron	GSK3B
Danthron	KDR
Danthron	CYP2C9
Danthron	PTPN1
Danthron	MMP13
Danthron	SRC
Danthron	PCK1
Danthron	LCK
Danthron	DPP4
Danthron	ADAM17
Danthron	PDE4B
Danthron	SYK
Danthron	RXRA
Danthron	PDPK1
Danthron	AR
Danthron	PYGL
Danthron	BST1
Danthron	AURKA
Danthron	ATOX1

Danthron	CSK
Danthron	FKBP1A
Danthron	NOS2
Danthron	GSTP1
Danthron	TTR
Danthron	NR1I2
Danthron	HCK
Danthron	NQO1
Danthron	RNASE3
Danthron	ALB
Danthron	JAK2
Danthron	BRAF
Danthron	DHFR
Danthron	ABL1
Danthron	PAK6
Danthron	PDE5A
Danthron	CDK5R1
Danthron	CTSK
Danthron	CTSS
Danthron	ZAP70
Danthron	XIAP
Danthron	GPI
Danthron	NR3C1
Danthron	LGALS2
Danthron	PRKCQ
Danthron	BIRC7
Danthron	F11
Danthron	MME
Danthron	PLEKHA4
Danthron	STAT1
Danthron	DTYMK
Danthron	DPEP1
Danthron	CCL5
Danthron	MAN1B1
Danthron	OTC
Danthron	BHMT
Danthron	NR1H4
Danthron	SULT1E1
Danthron	TGM3
Danthron	UCK2
Danthron	GCK
Danthron	GSTA1
Danthron	TGFB2
Danthron	VDR
Danthron	DHODH
Danthron	GSTT2
Danthron	ALAD
Danthron	GSTA3

Danthron	AMD1
Danthron	MET
Danthron	NT5M
Danthron	HADH
Danthron	RNASE2
Danthron	GSR
Danthron	GSTM1
Danthron	REN
Danthron	EIF4E
Danthron	ELANE
Danthron	AKR1C3
Danthron	FECH
Danthron	HMGCR
Danthron	ITPKA
Danthron	CHIT1
Danthron	PLA2G2A
Danthron	GSTM2
Danthron	AMY2A
Danthron	ADAM33
Danthron	CASP1
Danthron	NMNAT1
Danthron	HPGDS
Danthron	HINT1
Danthron	OAT
Danthron	BACE1
Danthron	HPRT1
Danthron	MMP9
Danthron	DUT
Danthron	RARA
Danthron	PIK3R1
Danthron	FGG
Danthron	EEA1
Danthron	IMPDH2
Danthron	RAB5A
Danthron	MMP1
Danthron	RARG
Danthron	DAPK1
Danthron	INSR
Danthron	ATIC
Danthron	FKBP3
Danthron	PPP1CC
Danthron	PNMT
Danthron	TAP1
Danthron	RFK
Danthron	BTK
Danthron	APAF1
Danthron	AKR1B1
Danthron	RAC1

Danthron	ARL5B
Danthron	SULT2A1
Danthron	SULT1A1
Danthron	SEC14L2
Danthron	RND3
Danthron	DCXR
Danthron	F7
Danthron	DCPS
Danthron	GSTZ1
Danthron	HRAS
Danthron	RAP2A
Danthron	RAN
Danthron	SIRT5
Danthron	HSD17B1
Danthron	AMY1A
Danthron	AMY1B
Danthron	AMY1C
Danthron	GNPDA2
Danthron	GNPDA1
Chrysophanol	MMP3
Chrysophanol	MAPK8
Chrysophanol	CES1
Chrysophanol	MAPK10
Chrysophanol	MAPK14
Chrysophanol	BCHE
Chrysophanol	CDK5R1
Chrysophanol	CFB
Chrysophanol	CA2
Chrysophanol	GSTP1
Chrysophanol	PIM1
Chrysophanol	MAOB
Chrysophanol	FGFR1
Chrysophanol	CHEK1
Chrysophanol	CTSB
Chrysophanol	ANXA5
Chrysophanol	DUSP6
Chrysophanol	AKR1B1
Chrysophanol	KIF11
Chrysophanol	PPARD
Chrysophanol	EGFR
Chrysophanol	MIF
Chrysophanol	HSPA8
Chrysophanol	RORA
Chrysophanol	ANG
Chrysophanol	F10
Chrysophanol	CDK2
Chrysophanol	HSP90AA1
Chrysophanol	F2

Chrysophanol	BCAT2
Chrysophanol	HSD11B1
Chrysophanol	METAP2
Chrysophanol	GSK3B
Chrysophanol	NOS3
Chrysophanol	TYMS
Chrysophanol	PPARG
Chrysophanol	PCK1
Chrysophanol	PDPK1
Chrysophanol	PGR
Chrysophanol	AKT1
Chrysophanol	ALB
Chrysophanol	HPN
Chrysophanol	HMGCR
Chrysophanol	CBR1
Chrysophanol	EPHX2
Chrysophanol	PTPN11
Chrysophanol	MMP12
Chrysophanol	FGFR2
Chrysophanol	AKR1C3
Chrysophanol	SEC14L2
Chrysophanol	DCK
Chrysophanol	PNMT
Chrysophanol	PAK7
Chrysophanol	ISG20
Chrysophanol	CCNA2
Chrysophanol	MET
Chrysophanol	ADK
Chrysophanol	CDA
Chrysophanol	HSD17B1
Chrysophanol	MMP8
Chrysophanol	PLAU
Chrysophanol	PAH
Chrysophanol	PDE4B
Chrysophanol	SRC
Chrysophanol	HDAC8
Chrysophanol	SOD2
Chrysophanol	PTPN1
Chrysophanol	ITK
Chrysophanol	ADH1C
Chrysophanol	AR
Chrysophanol	PRKACA
Chrysophanol	EPHB4
Chrysophanol	PDE4D
Chrysophanol	TTR
Chrysophanol	AMD1
Chrysophanol	HEXB
Chrysophanol	REN

Chrysophanol	KDR
Chrysophanol	DHFR
Chrysophanol	MMP13
Chrysophanol	SHBG
Chrysophanol	SERPINA1
Chrysophanol	CYP2C9
Chrysophanol	SYK
Chrysophanol	WAS
Chrysophanol	LCK
Chrysophanol	DPP4
Chrysophanol	PDE5A
Chrysophanol	ADAM17
Chrysophanol	PIK3CG
Chrysophanol	TGFBR1
Chrysophanol	TRAPPC3
Chrysophanol	ERBB4
Chrysophanol	TPI1
Chrysophanol	BLVRB
Chrysophanol	CTNNA1
Chrysophanol	ADH
Chrysophanol	FABP4
Chrysophanol	ABO
Chrysophanol	BST1
Chrysophanol	ACPP
Chrysophanol	RXRA
Chrysophanol	NR1H2
Chrysophanol	PYGL
Chrysophanol	AURKA
Chrysophanol	CTSK
Chrysophanol	PPARA
Chrysophanol	ATOX1
Chrysophanol	BIRC7
Chrysophanol	NR1H4
Chrysophanol	BRAF
Chrysophanol	XIAP
Chrysophanol	CSK
Chrysophanol	FKBP1A
Chrysophanol	HCK
Chrysophanol	IGF1
Chrysophanol	SULT2A1
Chrysophanol	MTHFD1
Chrysophanol	BACE1
Chrysophanol	NR1I2
Chrysophanol	HNF4G
Chrysophanol	NQO1
Chrysophanol	MAP2K1
Chrysophanol	IL2
Chrysophanol	CTSS

Chrysophanol	MTAP
Chrysophanol	SETD7
Chrysophanol	CCNT1
Chrysophanol	NR1I3
Chrysophanol	MDM2
Chrysophanol	THRB
Chrysophanol	ABL1
Chrysophanol	JAK2
Chrysophanol	JAK3
Chrysophanol	ESR1
Chrysophanol	PAK6
Chrysophanol	MAPK1
Chrysophanol	ZAP70
Chrysophanol	SULT1E1
Chrysophanol	AMY2A
Chrysophanol	PRKCQ
Chrysophanol	CCL5
Chrysophanol	GPI
Chrysophanol	NR3C1
Chrysophanol	PLA2G2A
Chrysophanol	LGALS2
Chrysophanol	F11
Chrysophanol	RBP4
Chrysophanol	MAPKAPK2
Chrysophanol	CASP3
Chrysophanol	DTYMK
Chrysophanol	DHODH
Chrysophanol	RARA
Chrysophanol	STAT1
Chrysophanol	MME
Chrysophanol	BHMT
Chrysophanol	SDS
Chrysophanol	HNMT
Chrysophanol	FABP6
Chrysophanol	DPEP1
Chrysophanol	FDPS
Chrysophanol	VDR
Chrysophanol	OTC
Chrysophanol	FABP7
Chrysophanol	MAN1B1
Chrysophanol	TGM3
Chrysophanol	HMOX1
Chrysophanol	PLEKHA4
Chrysophanol	UCK2
Chrysophanol	NT5M
Chrysophanol	CYP2C8
Chrysophanol	ACADM
Chrysophanol	TGFB2

Chrysophanol	TPH1
Chrysophanol	GSTA1
Chrysophanol	GCK
Chrysophanol	CRABP2
Chrysophanol	AKR1C2
Chrysophanol	ALAD
Chrysophanol	GSTT2
Chrysophanol	RARG
Chrysophanol	GSR
Chrysophanol	RNASE2
Chrysophanol	TEK
Chrysophanol	GSTM1
Chrysophanol	PPP1CC
Chrysophanol	ESR2
Chrysophanol	EIF4E
Chrysophanol	ELANE
Chrysophanol	KAT2B
Chrysophanol	WARS
Chrysophanol	IMPDH2
Chrysophanol	FECH
Chrysophanol	GSTA3
Chrysophanol	ITPKA
Chrysophanol	LCN2
Chrysophanol	GSTM2
Chrysophanol	LTA4H
Chrysophanol	ADAM33
Chrysophanol	RAB5A
Chrysophanol	CASP1
Chrysophanol	CHIT1
Chrysophanol	MMP9
Chrysophanol	FKBP1B
Chrysophanol	INSR
Chrysophanol	HPGDS
Chrysophanol	OAT
Chrysophanol	HINT1
Chrysophanol	HADH
Chrysophanol	NOS2
Chrysophanol	DUT
Chrysophanol	PIK3R1
Chrysophanol	HPRT1
Chrysophanol	KIT
Chrysophanol	RXRB
Chrysophanol	FGG
Chrysophanol	EEA1
Chrysophanol	MMP1
Chrysophanol	HSP90AB1
Chrysophanol	PCTP
Chrysophanol	NMNAT1

Chrysophanol	FKBP3
Chrysophanol	DAPK1
Chrysophanol	ATIC
Chrysophanol	TTPA
Chrysophanol	RARB
Chrysophanol	RFK
Chrysophanol	TAP1
Chrysophanol	GLO1
Chrysophanol	BTK
Chrysophanol	ARL5B
Chrysophanol	RAC1
Chrysophanol	GART
Chrysophanol	APAF1
Chrysophanol	RND3
Chrysophanol	HRAS
Chrysophanol	F7
Chrysophanol	IVD
Chrysophanol	PROCR
Chrysophanol	DCPS
Chrysophanol	RAP2A
Chrysophanol	RAN
Chrysophanol	AMY1A
Chrysophanol	AMY1B
Chrysophanol	AMY1C
Chrysophanol	GNPDA2
Chrysophanol	GNPDA1
Chrysophanol	RAB9B
Chrysophanol	RAB9A
beta-sitosterol	AKR1C2
beta-sitosterol	MAPKAPK2
beta-sitosterol	BMP2
beta-sitosterol	MAOB
beta-sitosterol	PIM1
beta-sitosterol	RORA
beta-sitosterol	THRB
beta-sitosterol	ALB
beta-sitosterol	STS
beta-sitosterol	PGR
beta-sitosterol	APOA2
beta-sitosterol	NR3C2
beta-sitosterol	CA2
beta-sitosterol	KIF11
beta-sitosterol	HSD17B1
beta-sitosterol	CASP7
beta-sitosterol	GC
beta-sitosterol	AR
beta-sitosterol	SEC14L2
beta-sitosterol	SULT2A1

beta-sitosterol	MAPK1
beta-sitosterol	ESR1
beta-sitosterol	CYP19A1
beta-sitosterol	MMP13
beta-sitosterol	F2
beta-sitosterol	KDR
beta-sitosterol	HDAC8
beta-sitosterol	HSD11B1
beta-sitosterol	PPARG
beta-sitosterol	PDE4B
beta-sitosterol	CDK2
beta-sitosterol	WAS
beta-sitosterol	MAPK14
beta-sitosterol	PNMT
beta-sitosterol	MAPK8
beta-sitosterol	NOS3
beta-sitosterol	EGFR
beta-sitosterol	ADAM17
beta-sitosterol	ANXA5
beta-sitosterol	NR1H4
beta-sitosterol	SRC
beta-sitosterol	PDPK1
beta-sitosterol	PTPN11
beta-sitosterol	TTR
beta-sitosterol	CES1
beta-sitosterol	BACE1
beta-sitosterol	CHEK1
beta-sitosterol	DHODH
beta-sitosterol	AKR1B1
beta-sitosterol	TRAPPC3
beta-sitosterol	TNNC1
beta-sitosterol	PRKACA
beta-sitosterol	TGFBR1
beta-sitosterol	PPP5C
beta-sitosterol	ITGAL
beta-sitosterol	CA1
beta-sitosterol	FABP6
beta-sitosterol	MDM2
beta-sitosterol	AKR1C1
beta-sitosterol	ESRRG
beta-sitosterol	SULT2B1
beta-sitosterol	F10
beta-sitosterol	GSTP1
beta-sitosterol	TTPA
beta-sitosterol	IGF1R
beta-sitosterol	SHBG
beta-sitosterol	EPHB4
beta-sitosterol	RBP4

beta-sitosterol	ADH
beta-sitosterol	SYK
beta-sitosterol	METAP2
beta-sitosterol	LCK
beta-sitosterol	BCHE
beta-sitosterol	TYMS
beta-sitosterol	BRAF
beta-sitosterol	GSR
beta-sitosterol	GSTA1
beta-sitosterol	DUSP6
beta-sitosterol	ADK
beta-sitosterol	NQO1
beta-sitosterol	ERBB4
beta-sitosterol	PDE4D
beta-sitosterol	NR1I3
beta-sitosterol	NR1H3
beta-sitosterol	FGFR2
beta-sitosterol	MMP2
beta-sitosterol	PLA2G2A
beta-sitosterol	CYP2C8
beta-sitosterol	FECH
beta-sitosterol	REN
beta-sitosterol	GSK3B
beta-sitosterol	DPP4
beta-sitosterol	PAK7
beta-sitosterol	LTA4H
beta-sitosterol	MTAP
beta-sitosterol	BLVRB
beta-sitosterol	HSP90AA1
beta-sitosterol	HPGDS
beta-sitosterol	AKR1C3
beta-sitosterol	ADH1C
beta-sitosterol	EPHX2
beta-sitosterol	MMP12
beta-sitosterol	NR1H2
beta-sitosterol	MMP8
beta-sitosterol	SERPINA1
beta-sitosterol	HNF4G
beta-sitosterol	ERRA
beta-sitosterol	MAPK10
beta-sitosterol	ESR2
beta-sitosterol	PDK2
beta-sitosterol	PGF
beta-sitosterol	CCNA2
beta-sitosterol	FABP3
beta-sitosterol	FABP7
beta-sitosterol	CTNNA1
beta-sitosterol	MET

beta-sitosterol	CASP3
beta-sitosterol	GLO1
beta-sitosterol	PDE5A
beta-sitosterol	RXRA
beta-sitosterol	PTPN1
beta-sitosterol	SOD2
beta-sitosterol	FGFR1
beta-sitosterol	PPARA
beta-sitosterol	PARP1
beta-sitosterol	FNTA
beta-sitosterol	LSS
beta-sitosterol	CDK6
beta-sitosterol	MAP2K1
beta-sitosterol	PPARD
beta-sitosterol	FABP5
beta-sitosterol	PDE3B
beta-sitosterol	DHFR
beta-sitosterol	CRABP2
beta-sitosterol	IGF1
beta-sitosterol	IL2
beta-sitosterol	RARG
beta-sitosterol	SULT1E1
beta-sitosterol	ABL1
beta-sitosterol	ITK
beta-sitosterol	ABO
beta-sitosterol	F7
beta-sitosterol	RARB
beta-sitosterol	SORD
beta-sitosterol	GM2A
beta-sitosterol	PIK3CG
beta-sitosterol	JAK3
beta-sitosterol	ELANE
beta-sitosterol	HNMT
beta-sitosterol	NR3C1
beta-sitosterol	MMP3
beta-sitosterol	MTHFD1
beta-sitosterol	NR1I2
beta-sitosterol	ALDH2
beta-sitosterol	LCN2
beta-sitosterol	VDR
beta-sitosterol	HSP90AB1
beta-sitosterol	RARA
beta-sitosterol	CYP2C9
beta-sitosterol	ZAP70
beta-sitosterol	GRB2
beta-sitosterol	CBR1
beta-sitosterol	AKT2
beta-sitosterol	PSAP

beta-sitosterol	TEK
beta-sitosterol	THRA
beta-sitosterol	HMGCR
beta-sitosterol	PRKCQ
beta-sitosterol	S100A9
beta-sitosterol	CTSS
beta-sitosterol	AURKA
beta-sitosterol	FKBP1A
beta-sitosterol	SETD7
beta-sitosterol	RXRB
beta-sitosterol	DPEP1
beta-sitosterol	NQO2
beta-sitosterol	JAK2
beta-sitosterol	HCK
beta-sitosterol	PIK3R1
beta-sitosterol	DCK
beta-sitosterol	CSK
beta-sitosterol	CTSB
beta-sitosterol	ACADM
beta-sitosterol	KIT
beta-sitosterol	PCTP
beta-sitosterol	BIRC7
beta-sitosterol	CMA1
beta-sitosterol	PPP1CC
beta-sitosterol	HMOX1
beta-sitosterol	GCK
beta-sitosterol	TGM3
beta-sitosterol	OAT
beta-sitosterol	TGFB2
beta-sitosterol	CD1A
beta-sitosterol	BCL2L1
beta-sitosterol	GART
beta-sitosterol	SDS
beta-sitosterol	CTSK
beta-sitosterol	BHMT
beta-sitosterol	EIF4E
beta-sitosterol	AMD1
beta-sitosterol	CASP1
beta-sitosterol	XIAP
beta-sitosterol	MME
beta-sitosterol	CTSF
beta-sitosterol	CHIT1
beta-sitosterol	PROCR
beta-sitosterol	GSTT2
beta-sitosterol	BCAT2
beta-sitosterol	INSR
beta-sitosterol	FKBP1B
beta-sitosterol	GSTM1

beta-sitosterol	MAOA
beta-sitosterol	FKBP3
beta-sitosterol	CALM1
beta-sitosterol	CALM2
beta-sitosterol	CALM3
Aloe-emodin	MMP3
Aloe-emodin	APOA2
Aloe-emodin	STS
Aloe-emodin	BCHE
Aloe-emodin	ALB
Aloe-emodin	ESR1
Aloe-emodin	MAPK8
Aloe-emodin	MAPK10
Aloe-emodin	CA2
Aloe-emodin	CA1
Aloe-emodin	PIM1
Aloe-emodin	PGR
Aloe-emodin	CFB
Aloe-emodin	BACE1
Aloe-emodin	MAOB
Aloe-emodin	F2
Aloe-emodin	CDK2
Aloe-emodin	TTR
Aloe-emodin	ESR2
Aloe-emodin	CFD
Aloe-emodin	BCAT2
Aloe-emodin	PDE4B
Aloe-emodin	AKR1B1
Aloe-emodin	ADH
Aloe-emodin	DHODH
Aloe-emodin	NQO1
Aloe-emodin	PDPK1
Aloe-emodin	ADH1B
Aloe-emodin	CCNA2
Aloe-emodin	KIF11
Aloe-emodin	PPARD
Aloe-emodin	AKR1C3
Aloe-emodin	F10
Aloe-emodin	TYMS
Aloe-emodin	ANG
Aloe-emodin	EGFR
Aloe-emodin	MTAP
Aloe-emodin	GC
Aloe-emodin	AMD1
Aloe-emodin	CHEK1
Aloe-emodin	ESRRG
Aloe-emodin	NOS3
Aloe-emodin	ADAM17

Aloe-emodin	MMP13
Aloe-emodin	MIF
Aloe-emodin	HSP90AA1
Aloe-emodin	HSD17B1
Aloe-emodin	HSD11B1
Aloe-emodin	TGFBR1
Aloe-emodin	AR
Aloe-emodin	PDE4D
Aloe-emodin	DHFR
Aloe-emodin	FGFR1
Aloe-emodin	PDE5A
Aloe-emodin	PRKACA
Aloe-emodin	CSNK1G2
Aloe-emodin	GBA
Aloe-emodin	AKR1C1
Aloe-emodin	SERPINA1
Aloe-emodin	SHBG
Aloe-emodin	PLK1
Aloe-emodin	METAP2
Aloe-emodin	AMY2A
Aloe-emodin	MAPK14
Aloe-emodin	SEC14L2
Aloe-emodin	SRC
Aloe-emodin	EPHX2
Aloe-emodin	KDR
Aloe-emodin	CBR1
Aloe-emodin	PTPN11
Aloe-emodin	EPHB4
Aloe-emodin	YARS
Aloe-emodin	PAH
Aloe-emodin	NR3C2
Aloe-emodin	ISG20
Aloe-emodin	F7
Aloe-emodin	ARHGAP1
Aloe-emodin	PLAU
Aloe-emodin	DCK
Aloe-emodin	DAPK1
Aloe-emodin	CDK6
Aloe-emodin	MMP8
Aloe-emodin	RXRA
Aloe-emodin	SOD2
Aloe-emodin	PNP
Aloe-emodin	HPN
Aloe-emodin	NQO2
Aloe-emodin	GSK3B
Aloe-emodin	LSS
Aloe-emodin	PNMT
Aloe-emodin	RHOA

Aloe-emodin	SORD
Aloe-emodin	NR1I2
Aloe-emodin	LCK
Aloe-emodin	ADH1C
Aloe-emodin	MMP12
Aloe-emodin	CSNK2A1
Aloe-emodin	PCK1
Aloe-emodin	TNK2
Aloe-emodin	PYGL
Aloe-emodin	SULT2A1
Aloe-emodin	HDAC8
Aloe-emodin	BST1
Aloe-emodin	GSTP1
Aloe-emodin	UCK2
Aloe-emodin	THRB
Aloe-emodin	CDA
Aloe-emodin	SYK
Aloe-emodin	ALDH2
Aloe-emodin	HEXB
Aloe-emodin	ABO
Aloe-emodin	UMPS
Aloe-emodin	PDE3B
Aloe-emodin	PARP1
Aloe-emodin	CYP2C9
Aloe-emodin	ATOX1
Aloe-emodin	IGF1R
Aloe-emodin	DPP4
Aloe-emodin	CASP3
Aloe-emodin	NR1H4
Aloe-emodin	HMGCR
Aloe-emodin	AHCY
Aloe-emodin	TPI1
Aloe-emodin	MTHFD1
Aloe-emodin	CCL5
Aloe-emodin	AKT1
Aloe-emodin	PTPN1
Aloe-emodin	AURKA
Aloe-emodin	GSTA1
Aloe-emodin	CTNNA1
Aloe-emodin	JAK2
Aloe-emodin	RNASE3
Aloe-emodin	GCK
Aloe-emodin	SULT2B1
Aloe-emodin	PADI4
Aloe-emodin	TRAPPC3
Aloe-emodin	ALDOA
Aloe-emodin	LGALS2
Aloe-emodin	B3GAT1

Aloe-emodin	ELANE
Aloe-emodin	ERBB4
Aloe-emodin	FKBP1A
Aloe-emodin	CTSK
Aloe-emodin	HSPA8
Aloe-emodin	JAK3
Aloe-emodin	MET
Aloe-emodin	AKR1C2
Aloe-emodin	RAB11A
Aloe-emodin	IMPDH2
Aloe-emodin	FECH
Aloe-emodin	SULT1E1
Aloe-emodin	GSTT2
Aloe-emodin	HCK
Aloe-emodin	SETD7
Aloe-emodin	CTSS
Aloe-emodin	NR3C1
Aloe-emodin	IGF1
Aloe-emodin	CDK5R1
Aloe-emodin	SSE1
Aloe-emodin	ACADM
Aloe-emodin	ARG1
Aloe-emodin	CTSF
Aloe-emodin	GSR
Aloe-emodin	APRT
Aloe-emodin	CD1A
Aloe-emodin	ADK
Aloe-emodin	CLK1
Aloe-emodin	RAB5A
Aloe-emodin	PPCDC
Aloe-emodin	BRAF
Aloe-emodin	NOS2
Aloe-emodin	CBS
Aloe-emodin	THRA
Aloe-emodin	SHMT1
Aloe-emodin	GSTA3
Aloe-emodin	FABP6
Aloe-emodin	F11
Aloe-emodin	NR1H2
Aloe-emodin	MAPK1
Aloe-emodin	CYP2C8
Aloe-emodin	GPI
Aloe-emodin	KAT2B
Aloe-emodin	CHIT1
Aloe-emodin	DTYMK
Aloe-emodin	XIAP
Aloe-emodin	FABP3
Aloe-emodin	TPH1

Aloe-emodin	CRABP2
Aloe-emodin	HNF4G
Aloe-emodin	DPEP1
Aloe-emodin	FDPS
Aloe-emodin	TGM3
Aloe-emodin	FABP7
Aloe-emodin	ZAP70
Aloe-emodin	CASP1
Aloe-emodin	NMNAT1
Aloe-emodin	IL2
Aloe-emodin	HRAS
Aloe-emodin	RBP4
Aloe-emodin	VDR
Aloe-emodin	CDK7
Aloe-emodin	MMP2
Aloe-emodin	OTC
Aloe-emodin	LYZ
Aloe-emodin	RARG
Aloe-emodin	ABL1
Aloe-emodin	MAN1B1
Aloe-emodin	AGXT
Aloe-emodin	BHMT
Aloe-emodin	WARS
Aloe-emodin	BIRC7
Aloe-emodin	TK1
Aloe-emodin	CTSG
Aloe-emodin	STAT1
Aloe-emodin	PLA2G2A
Aloe-emodin	S100A9
Aloe-emodin	MME
Aloe-emodin	MAP2K1
Aloe-emodin	PLEKHA4
Aloe-emodin	EPHA2
Aloe-emodin	MAPKAPK2
Aloe-emodin	ALAD
Aloe-emodin	FOLH1
Aloe-emodin	RARA
Aloe-emodin	AKT2
Aloe-emodin	HPGDS
Aloe-emodin	IMPDH1
Aloe-emodin	MMP9
Aloe-emodin	KIT
Aloe-emodin	TEK
Aloe-emodin	PPARA
Aloe-emodin	PIK3R1
Aloe-emodin	EIF4E
Aloe-emodin	HADH
Aloe-emodin	NT5M

Aloe-emodin	GP1BA
Aloe-emodin	RARB
Aloe-emodin	REN
Aloe-emodin	RNASE2
Aloe-emodin	TGFB2
Aloe-emodin	CTSB
Aloe-emodin	HINT1
Aloe-emodin	GSTM1
Aloe-emodin	ITPKA
Aloe-emodin	LGALS3
Aloe-emodin	DUT
Aloe-emodin	FKBP1B
Aloe-emodin	INSR
Aloe-emodin	HPRT1
Aloe-emodin	ATIC
Aloe-emodin	GSTM2
Aloe-emodin	ARL5A
Aloe-emodin	LTA4H
Aloe-emodin	PPP1CC
Aloe-emodin	GSTO1
Aloe-emodin	NDST1
Aloe-emodin	HNMT
Aloe-emodin	OAT
Aloe-emodin	PKLR
Aloe-emodin	FGG
Aloe-emodin	RXRB
Aloe-emodin	CMA1
Aloe-emodin	ADAM33
Aloe-emodin	GMPR
Aloe-emodin	EEA1
Aloe-emodin	MMP1
Aloe-emodin	GLO1
Aloe-emodin	TTPA
Aloe-emodin	PCMT1
Aloe-emodin	CRYZ
Aloe-emodin	DDX39B
Aloe-emodin	GMPR2
Aloe-emodin	TAP1
Aloe-emodin	CDC42
Aloe-emodin	GART
Aloe-emodin	PFKFB1
Aloe-emodin	RAN
Aloe-emodin	DCXR
Aloe-emodin	FKBP3
Aloe-emodin	ARL5B
Aloe-emodin	RAC1
Aloe-emodin	GSTZ1
Aloe-emodin	APAF1

Aloe-emodin	RORA
Aloe-emodin	RFK
Aloe-emodin	NME2
Aloe-emodin	BTK
Aloe-emodin	AMY1A
Aloe-emodin	AMY1B
Aloe-emodin	AMY1C
Aloe-emodin	GNPDA2
Aloe-emodin	GNPDA1
Aloe-emodin	RAB9B
Aloe-emodin	RAB9A
(-)-catechin	AR
(-)-catechin	CYP19A1
(-)-catechin	ESRRG
(-)-catechin	ESR1
(-)-catechin	CA2
(-)-catechin	BCHE
(-)-catechin	BACE1
(-)-catechin	HCK
(-)-catechin	GSR
(-)-catechin	PGR
(-)-catechin	TTR
(-)-catechin	CCNA2
(-)-catechin	CDK2
(-)-catechin	CHEK1
(-)-catechin	PIM1
(-)-catechin	MAPK14
(-)-catechin	ESR2
(-)-catechin	CA12
(-)-catechin	HSD17B1
(-)-catechin	SORD
(-)-catechin	MMP13
(-)-catechin	ADAM17
(-)-catechin	EPHB4
(-)-catechin	KDR
(-)-catechin	CFD
(-)-catechin	CES1
(-)-catechin	GBA
(-)-catechin	CA1
(-)-catechin	RXRA
(-)-catechin	BRAF
(-)-catechin	PDE4B
(-)-catechin	MMP8
(-)-catechin	CFB
(-)-catechin	PDE4D
(-)-catechin	LCN2
(-)-catechin	DPP4
(-)-catechin	AKR1C3

(-)-catechin	PAH
(-)-catechin	AKT1
(-)-catechin	NOS3
(-)-catechin	SRC
(-)-catechin	PNP
(-)-catechin	FNTA
(-)-catechin	SHBG
(-)-catechin	BCAT2
(-)-catechin	PLA2G10
(-)-catechin	PNPO
(-)-catechin	KIF11
(-)-catechin	CSNK1G2
(-)-catechin	SULT2A1
(-)-catechin	AZGP1
(-)-catechin	NR3C2
(-)-catechin	CTSK
(-)-catechin	PIK3CG
(-)-catechin	AKR1C1
(-)-catechin	CTNNA1
(-)-catechin	CDK6
(-)-catechin	TGFBR2
(-)-catechin	PRKACA
(-)-catechin	DHFR
(-)-catechin	F10
(-)-catechin	ANG
(-)-catechin	HSP90AA1
(-)-catechin	F2
(-)-catechin	IMPA1
(-)-catechin	LTA4H
(-)-catechin	HSD11B1
(-)-catechin	CRAT
(-)-catechin	NQO1
(-)-catechin	CBR1
(-)-catechin	HDAC8
(-)-catechin	F7
(-)-catechin	PPP5C
(-)-catechin	MMP12
(-)-catechin	MIF
(-)-catechin	LSS
(-)-catechin	RHOA
(-)-catechin	MET
(-)-catechin	PCK1
(-)-catechin	MTAP
(-)-catechin	PGF
(-)-catechin	METAP2
(-)-catechin	SOD2
(-)-catechin	REN
(-)-catechin	HPGDS

(-)-catechin	PDPK1
(-)-catechin	DCK
(-)-catechin	YARS
(-)-catechin	JAK3
(-)-catechin	MMP3
(-)-catechin	LCK
(-)-catechin	ABO
(-)-catechin	TYMS
(-)-catechin	NR1H4
(-)-catechin	ADH
(-)-catechin	AMD1
(-)-catechin	NR1H2
(-)-catechin	MAPK8
(-)-catechin	CYP2C9
(-)-catechin	PLAU
(-)-catechin	ADK
(-)-catechin	NR1H3
(-)-catechin	RAB11A
(-)-catechin	AMY2A
(-)-catechin	FGFR1
(-)-catechin	ISG20
(-)-catechin	AKR1B1
(-)-catechin	HK1
(-)-catechin	GSK3B
(-)-catechin	ALB
(-)-catechin	SERPINA1
(-)-catechin	HSPA8
(-)-catechin	PTPN1
(-)-catechin	PDK2
(-)-catechin	ADH1C
(-)-catechin	GSTP1
(-)-catechin	IMPDH2
(-)-catechin	PDHB
(-)-catechin	SHMT1
(-)-catechin	ELANE
(-)-catechin	BAG1
(-)-catechin	THRB
(-)-catechin	MAN1B1
(-)-catechin	PDE5A
(-)-catechin	RBP4
(-)-catechin	AKR1C2
(-)-catechin	CD1A
(-)-catechin	CDA
(-)-catechin	MAPK10
(-)-catechin	AHCY
(-)-catechin	ZAP70
(-)-catechin	JAK2
(-)-catechin	RHEB

(-)-catechin	ALDH2
(-)-catechin	RAB5A
(-)-catechin	DUSP6
(-)-catechin	ERBB4
(-)-catechin	EGFR
(-)-catechin	HPN
(-)-catechin	GSTA1
(-)-catechin	OTC
(-)-catechin	RAC2
(-)-catechin	ATOX1
(-)-catechin	MTHFD1
(-)-catechin	CBS
(-)-catechin	IGF1R
(-)-catechin	NMNAT1
(-)-catechin	RNASEL
(-)-catechin	CSNK2A1
(-)-catechin	AURKA
(-)-catechin	MAPKAPK2
(-)-catechin	ITK
(-)-catechin	MAOB
(-)-catechin	TPI1
(-)-catechin	SYK
(-)-catechin	FECH
(-)-catechin	TNK2
(-)-catechin	UCK2
(-)-catechin	PYGL
(-)-catechin	MME
(-)-catechin	FABP6
(-)-catechin	THRA
(-)-catechin	REG1A
(-)-catechin	HEXB
(-)-catechin	ARHGAP1
(-)-catechin	GPI
(-)-catechin	TK1
(-)-catechin	IGF1
(-)-catechin	CLK1
(-)-catechin	XIAP
(-)-catechin	ARSA
(-)-catechin	GALE
(-)-catechin	ALDOA
(-)-catechin	TGM3
(-)-catechin	B3GAT1
(-)-catechin	CCL5
(-)-catechin	FKBP1A
(-)-catechin	BHMT
(-)-catechin	INSR
(-)-catechin	BST1
(-)-catechin	NR1I2

(-)-catechin	PTPN11
(-)-catechin	UMPS
(-)-catechin	EPHX2
(-)-catechin	LGALS2
(-)-catechin	LYZ
(-)-catechin	S100A9
(-)-catechin	NR3C1
(-)-catechin	SDS
(-)-catechin	FABP4
(-)-catechin	SULT2B1
(-)-catechin	SSE1
(-)-catechin	TGM2
(-)-catechin	CTSS
(-)-catechin	BIRC7
(-)-catechin	HRAS
(-)-catechin	CDK5R1
(-)-catechin	DHODH
(-)-catechin	SULT1E1
(-)-catechin	RNASE3
(-)-catechin	NOS2
(-)-catechin	ABL1
(-)-catechin	ST14
(-)-catechin	FABP3
(-)-catechin	ACPP
(-)-catechin	PLA2G2A
(-)-catechin	MMP9
(-)-catechin	GCK
(-)-catechin	PRKCQ
(-)-catechin	EIF4E
(-)-catechin	KIT
(-)-catechin	PAK6
(-)-catechin	VDR
(-)-catechin	GSTT2
(-)-catechin	DPEP1
(-)-catechin	PPCDC
(-)-catechin	NT5M
(-)-catechin	TREM1
(-)-catechin	GLTP
(-)-catechin	CRABP2
(-)-catechin	TEK
(-)-catechin	MAPK1
(-)-catechin	LGALS3
(-)-catechin	APRT
(-)-catechin	HMGCR
(-)-catechin	IL2
(-)-catechin	RARA
(-)-catechin	SRM
(-)-catechin	GSTA3

(-)-catechin	KYAT1
(-)-catechin	GSTM1
(-)-catechin	HAGH
(-)-catechin	CMA1
(-)-catechin	OAT
(-)-catechin	PAPSS1
(-)-catechin	GART
(-)-catechin	TRAPPC3
(-)-catechin	PITPNA
(-)-catechin	PKLR
(-)-catechin	AGXT
(-)-catechin	TPH1
(-)-catechin	ARG2
(-)-catechin	HSP90AB1
(-)-catechin	CASP3
(-)-catechin	TPSB2
(-)-catechin	HINT1
(-)-catechin	EPHA2
(-)-catechin	TGFB2
(-)-catechin	MAP2K1
(-)-catechin	MMP16
(-)-catechin	HPRT1
(-)-catechin	DTYMK
(-)-catechin	IMPDH1
(-)-catechin	CTSL
(-)-catechin	ACAT1
(-)-catechin	RARG
(-)-catechin	GMPR
(-)-catechin	CHIT1
(-)-catechin	ADAM33
(-)-catechin	ACADM
(-)-catechin	PLEKHA4
(-)-catechin	CTSB
(-)-catechin	PPARG
(-)-catechin	LDHB
(-)-catechin	PPP1CC
(-)-catechin	EEA1
(-)-catechin	CD209
(-)-catechin	HMOX1
(-)-catechin	PIK3R1
(-)-catechin	SEC14L2
(-)-catechin	GSTO1
(-)-catechin	FKBP1B
(-)-catechin	HNMT
(-)-catechin	SELP
(-)-catechin	RARB
(-)-catechin	CASP1
(-)-catechin	GSTM2

(-)-catechin	CTSF
(-)-catechin	FDPS
(-)-catechin	CDC42
(-)-catechin	HADH
(-)-catechin	PPARA
(-)-catechin	ARG1
(-)-catechin	STAT1
(-)-catechin	PNMT
(-)-catechin	NDST1
(-)-catechin	GP1BA
(-)-catechin	RAN
(-)-catechin	AMY1A
(-)-catechin	AMY1B
(-)-catechin	AMY1C
(-)-catechin	GNPDA2
(-)-catechin	GNPDA1
(-)-catechin	RAB9B
(-)-catechin	RAB9A
