# Supplementary material for: Mechanism of Radix Rhei Et Rhizome Intervention in Cerebral Infarction: A Research Based on Chemoinformatics and Systematic Pharmacology
Source: Evid Based Complement Alternat Med. 2021 Sep 6;2021:6789835. doi: 10.1155/2021/6789835 (PMC8440083; doi:10.1155/2021/6789835)
Supplement: Supplementary Materials — Table S1: potential targets for potential compounds; Table S2: proteomics data; Table S3: CI gene; Table S4: enrichment analysis of clusters based on gene ontology (GO) annotation of Radix Rhei Et Rhizome-CI PPI network; Table S5: pathway enrichment analysis of Radix Rhei Et Rhizome-CI PPI network; Table S6: reactome pathways of Radix Rhei Et Rhizome-CI PPI network; and Table S7: the biological processes, signaling pathways, and reactome of proteomics proteins' PPI network. [file 6789835.f1.zip › 6789835.f1/Table S2.pdf]

Table S2 Proteomics Data

Uniprot Accessi	Gene Name	Protein identity	Peptides (!	AVG.	%Cov (95
G3V7C6	Tubb4b	Tubulin beta-4B chain	274	2.43	76.64
Q9WTY2	Rem2	GTP-binding protein REM 2	2	1.88	4.98
Q04940	Nrgn	Neurogranin	7	3.81	46.15
P10818	Cox6a1	Cytochrome c oxidase subunit 6A1, mit	3	2.21	27.93
P04094	Penk	Proenkephalin-A	3	1.7	11.15
P22057	Ptgds	Prostaglandin-H2 D-isomerase	6	1.76	29.63
P35332	Hpcal4	Hippocalcin-like protein 4	12	2.15	49.21
P60841	Ensa	Alpha-endosulfine	7	2.99	47.11
Q3ZAV2	Ybx1	Nuclease-sensitive element-binding pro	4	1.6	27.3
G3V997	Dcx	Neuronal migration protein doublecorti	9	1.8	20.82
Q6AXS4	Atp6ap2	Renin receptor	8	1.73	16
P37377	Snca	Alpha-synuclein	22	2.37	80.71
O35796	C1qbp	Complement component 1 Q subcompo	8	1.63	28.32
B7X6I3	Cend1	C38 protein	19	2	56.38
F8WFH6	Fam131b	Protein FAM131B	7	1.89	20.28
Q5U2U8	Bag3	Bcl2-associated athanogene 3	9	1.6	17.42
P35213	Ywhab	14-3-3 protein beta/alpha	65	1.92	78.46
O70257	Stx7	Syntaxin-7	13	3.47	39.85
A0A0G2K0M8	Ncam1	Neural cell adhesion molecule 1	77	2.44	50.3
P07825	Syp	Synaptophysin	19	1.55	40.07
P07171	Calb1	Calbindin	16	1.51	41.38
D3ZUX5	Chchd3	MICOS complex subunit	13	3.34	35.68
P62982	Rps27a	Ubiquitin-40S ribosomal protein S27a	23	1.62	54.49
D4A0T0	Ndufb10	Protein Ndufb10	15	2.43	61.93
Q09073	Slc25a5	ADP/ATP translocase 2	46	2.03	61.07
A0A0H2UHV6	Ppp3r1	Calcineurin subunit B type 1	17	1.55	61.25
D4A5L9	LOC679794	Protein LOC679794	24	3.31	71.43
P61589	Rhoa	Transforming protein RhoA	24	2.52	64.77
F1M378	Unc13a	Protein unc-13 homolog A	11	4.26	6.01
O88767	Park7	Protein deglycase DJ-1	22	1.76	91.53
D4AA63	Ubqln2	Protein Ubqln2	22	1.53	27.12
P14604	Echs1	Enoyl-CoA hydratase, mitochondrial	20	2.09	53.45
P08461	Dlat	Dihydrolipoyllysine-residue acetyltrans	25	3.38	35.6
P63086	Mapk1	Mitogen-activated protein kinase 1	26	1.63	49.16
Q6P6R2	Dld	Dihydrolipoyl dehydrogenase, mitochon	31	1.98	54.03
Q05175	Basp1	Brain acid soluble protein 1	82	4.18	86.82
P62161	Calm1	Calmodulin	80	2.64	89.93
P11275	Camk2a	Calcium/calmodulin-dependent protein	76	1.73	64.44
P35565	Canx	Calnexin	31	1.5	37.39
P07936	Gap43	Neuromodulin	48	3.07	84.51
Q9Z0W5	Pacsin1	Protein kinase C and casein kinase subs	50	1.61	56.46
P21707	Syt1	Synaptotagmin-1	37	1.79	45.61
Q5RKI0	Wdr1	WD repeat-containing protein 1	33	1.53	46.37
P63102	Ywhaz	14-3-3 protein zeta/delta	77	1.9	85.31
P06761	Hspa5	78 kDa glucose-regulated protein	57	1.55	55.2
P05708	Hk1	Hexokinase-1	80	1.5	47.17

Q63198	Cntn1	Contactin-1	77	1.74	48.29
D3ZMI4	Epb41l1	Band 4.1-like protein 1	52	1.77	34.24
P09951	Syn1	Synapsin-1	152	2.34	72.3
F1LQ81	Nsf	N-ethylmaleimide sensitive fusion prote	86	1.84	69.22
D3ZQQ5	Dnm1	Dynamin-1	107	1.52	68.06
G3V984	Bsn	Protein bassoon	111	1.53	32.42
F1M779	Cltc	Clathrin heavy chain	213	1.76	58.69
A0A0G2K839	Clcn5	Chloride channel protein	3	0.02	4.41
G3V8L6	Kcna6	Potassium voltage-gated channel subfar	2	0.65	6.04
D3ZV91	L3hypdh	Protein L3hypdh	2	0.48	7.34
F1LSV4	Sptlc2	Protein Sptlc2	3	0.52	5.18
A0A0G2K1A2	Mpo	Protein Mpo	3	0.65	3.76
P28572	Slc6a9	Sodium- and chloride-dependent glycin	5	0.6	8.62
P55009	Aif1	Allograft inflammatory factor 1	5	0.6	23.13
Q6AYR8	Scrn2	Secernin-2	6	0.43	13
A0A0G2JWX4	Krt2	Keratin, type II cytoskeletal 2 epiderma	4	0.45	9.36
G3V6M3	Syt2	Synaptotagmin II	17	0.48	29.38
Q9R0I8	Pip4k2a	Phosphatidylinositol 5-phosphate 4-kin	14	0.46	22.66
Q7TP91	Surf4	Ab1-205	9	0.55	9.55
P0DMW1	Hspa1b	Heat shock 70 kDa protein 1B	73	0.51	41.34
P60203	Plp1	Myelin proteolipid protein	50	0.2	31.41
Q63345	Mog	Myelin-oligodendrocyte glycoprotein	14	0.39	36.73
A0A0G2JWM2	Sirt2	NAD-dependent protein deacetylase sir	34	0.61	52.32
Q63357	Myo1d	Unconventional myosin-Id	27	0.48	21.27
E9PTX9	Slc12a2	Protein Slc12a2	26	0.44	21.28
P02688	Mbp	Myelin basic protein	88	0.23	66.67
F1LRZ7	Nefh	Neurofilament heavy polypeptide	55	0.47	41.26
P19527	Nefl	Neurofilament light polypeptide	87	0.32	58.49
P13233	Cnp	2′,3′-cyclic-nucleotide 3′-phospho	83	0.47	74.52
P12839	Nefm	Neurofilament medium polypeptide	86	0.49	49.88

)
