# Supplementary material for: Mechanism of Radix Rhei Et Rhizome Intervention in Cerebral Infarction: A Research Based on Chemoinformatics and Systematic Pharmacology
Source: Evid Based Complement Alternat Med. 2021 Sep 6;2021:6789835. doi: 10.1155/2021/6789835 (PMC8440083; doi:10.1155/2021/6789835)
Supplement: Supplementary Materials — Table S1: potential targets for potential compounds; Table S2: proteomics data; Table S3: CI gene; Table S4: enrichment analysis of clusters based on gene ontology (GO) annotation of Radix Rhei Et Rhizome-CI PPI network; Table S5: pathway enrichment analysis of Radix Rhei Et Rhizome-CI PPI network; Table S6: reactome pathways of Radix Rhei Et Rhizome-CI PPI network; and Table S7: the biological processes, signaling pathways, and reactome of proteomics proteins' PPI network. [file 6789835.f1.zip › 6789835.f1/Table S3.pdf]

Table S3 CI Gene

Gene Symbol	Description	Relevance score
PRKCH	Protein Kinase C Eta	28.05
F2	Coagulation Factor II, Thrombin	23.33
ALOX5AP	Arachidonate 5-Lipoxygenase A	22.8
NOTCH3	Notch 3	20.6
F5	Coagulation Factor V	20.47
NOS3	Nitric Oxide Synthase 3	19.32
RNF213	Ring Finger Protein 213	17.17
APOE	Apolipoprotein E	17.05
APP	Amyloid Beta Precursor Protein	16.73
ACE	Angiotensin I Converting Enzym	16.03
APOH	Apolipoprotein H	15.55
SERPINC1	Serpin Family C Member 1	15.38
PIK3CA	Phosphatidylinositol-4,5-Bispho	15.28
MTHFR	Methylenetetrahydrofolate Redu	15.18
FBN1	Fibrillin 1	14.98
MT-TL1	Mitochondrially Encoded TRNA	14.98
F3	Coagulation Factor III, Tissue Fa	14.94
SERPINE1	Serpin Family E Member 1	14.68
CRP	C-Reactive Protein	14.53
THBD	Thrombomodulin	14.35
VWF	Von Willebrand Factor	14.05
SELP	Selectin P	13.08
TBXA2R	Thromboxane A2 Receptor	12.65
APOB	Apolipoprotein B	12.59
ENO2	Enolase 2	12.3
ACTA2	Actin, Alpha 2, Smooth Muscle,	12.2
GUCY1A1	Guanylate Cyclase 1 Soluble Sul	12.2
PLAT	Plasminogen Activator, Tissue T	12.11
TLR4	Toll Like Receptor 4	12.03
PF4	Platelet Factor 4	12.02
FGA	Fibrinogen Alpha Chain	11.85
F7	Coagulation Factor VII	11.66
PDE4D	Phosphodiesterase 4D	11.61
PLA2G7	Phospholipase A2 Group VII	11.56
AGTR1	Angiotensin II Receptor Type 1	11.45
SULT1A3	Sulfotransferase Family 1A Mer	11.31
PLG	Plasminogen	11.28
PON1	Paraoxonase 1	11.24
LPA	Lipoprotein(A)	11.18
AIF1	Allograft Inflammatory Factor 1	11.1
PPBP	Pro-Platelet Basic Protein	10.92
NES	Nestin	10.7
PTGIS	Prostaglandin I2 Synthase	10.56
HSPA4	Heat Shock Protein Family A (H	10.41
SERPIND1	Serpin Family D Member 1	10.14
PROZ	Protein Z, Vitamin K Dependent	9.89

SERPINF2	Serpin Family F Member 2	9.72
CASP3	Caspase 3	9.14
GDNF	Glial Cell Derived Neurotrophic	9.02
AQP4	Aquaporin 4	8.94
FGB	Fibrinogen Beta Chain	8.9
BMP7	Bone Morphogenetic Protein 7	8.67
SLC1A2	Solute Carrier Family 1 Member	8.44
MAP2	Microtubule Associated Protein	8.41
ENTPD1	Ectonucleoside Triphosphate Di	8.16
AKT1	AKT Serine/Threonine Kinase 1	8.14
PDGFRB	Platelet Derived Growth Factor	8.14
PDGFB	Platelet Derived Growth Factor	8.14
COG2	Component Of Oligomeric Golg	8.01
HMGCR	3-Hydroxy-3-Methylglutaryl-Co	7.92
HMGB1	High Mobility Group Box 1	7.92
IL6	Interleukin 6	7.87
CREB1	CAMP Responsive Element Bin	7.79
GP6	Glycoprotein VI Platelet	7.79
RTN4	Reticulon 4	7.79
HBA1	Hemoglobin Subunit Alpha 1	7.79
MROS	Melkersson-Rosenthal Syndrom	7.79
CYCS	Cytochrome C, Somatic	7.49
P2RY12	Purinergic Receptor P2Y12	7.49
REN	Renin	7.49
ASAH1	N-Acylsphingosine Amidohydro	7.49
DCX	Doublecortin	7.49
SLC20A2	Solute Carrier Family 20 Memb	7.49
MBP	Myelin Basic Protein	7.49
AGTR2	Angiotensin II Receptor Type 2	7.49
SMARCAL1	SWI/SNF Related, Matrix Assoc	7.49
XPR1	Xenotropic And Polytropic Retr	7.49
BRCC3	BRCA1/BRCA2-Containing Co	7.49
NGB	Neuroglobin	7.49
TSPAN33	Tetraspanin 33	7.49
MIAT	Myocardial Infarction Associate	7.49
MALAT1	Metastasis Associated Lung Ade	7.49
CDKN2B-AS1	CDKN2B Antisense RNA 1	7.49
IBGC2	Basal Ganglia Calcification, Idic	7.49
MYMY4	Moyamoya Disease 4	7.49
SOD1	Superoxide Dismutase 1	7.18
F12	Coagulation Factor XII	6.72
MMP9	Matrix Metallopeptidase 9	6.41
TNF	Tumor Necrosis Factor	6.15
TGFB1	Transforming Growth Factor Be	5.75
APOA1	Apolipoprotein A1	5.32
LOC100294362	Uncharacterized LOC10029436	5.3
ADIPOQ	Adiponectin, C1Q And Collager	5.25
AGT	Angiotensinogen	5.14

LPL	Lipoprotein Lipase	5.08
CD59	CD59 Molecule (CD59 Blood G	4.4
FGF2	Fibroblast Growth Factor 2	4.29
IL1A	Interleukin 1 Alpha	4.29
TNFSF4	TNF Superfamily Member 4	4.29
ESR1	Estrogen Receptor 1	4.25
CCL5	C-C Motif Chemokine Ligand 5	4.21
RBP4	Retinol Binding Protein 4	4.07
PTGS2	Prostaglandin-Endoperoxide Syn	4.01
ALDH2	Aldehyde Dehydrogenase 2 Fam	4.01
PPARG	Peroxisome Proliferator Activate	3.96
IL10	Interleukin 10	3.91
IL1B	Interleukin 1 Beta	3.89
APOA5	Apolipoprotein A5	3.89
HGF	Hepatocyte Growth Factor	3.86
VEGFA	Vascular Endothelial Growth Fa	3.86
ABCA1	ATP Binding Cassette Subfamily	3.86
CCL2	C-C Motif Chemokine Ligand 2	3.86
IL4	Interleukin 4	3.8
OLR1	Oxidized Low Density Lipoprote	3.8
SMARCA4	SWI/SNF Related, Matrix Assoc	3.75
SMARCA2	SWI/SNF Related, Matrix Assoc	3.75
GIPC1	GIPC PDZ Domain Containing F	3.75
BAZ1A	Bromodomain Adjacent To Zinc	3.75
SMARCA1	SWI/SNF Related, Matrix Assoc	3.75
TAX1BP3	Tax1 Binding Protein 3	3.75
ASTN1	Astrotactin 1	3.75
ACTA2-AS1	ACTA2 Antisense RNA 1	3.75
CPB2	Carboxypeptidase B2	3.74
CXCL8	C-X-C Motif Chemokine Ligand	3.68
MIR223	MicroRNA 223	3.68
CST3	Cystatin C	3.6
IL1RN	Interleukin 1 Receptor Antagoni	3.53
NPPB	Natriuretic Peptide B	3.52
ALB	Albumin	3.46
ABCC8	ATP Binding Cassette Subfamily	3.43
BSG	Basigin (Ok Blood Group)	3.43
TNFRSF4	TNF Receptor Superfamily Mem	3.43
NPY	Neuropeptide Y	3.38
CBS	Cystathionine-Beta-Synthase	3.28
TSPO	Translocator Protein	3.28
EDN1	Endothelin 1	3.21
BDNF	Brain Derived Neurotrophic Fac	3.13
FABP1	Fatty Acid Binding Protein 1	3.13
NCF1	Neutrophil Cytosolic Factor 1	3.08
CX3CR1	C-X3-C Motif Chemokine Recep	3.07
IGF1	Insulin Like Growth Factor 1	3.03
NOS2	Nitric Oxide Synthase 2	3.03

ADAMTS13	ADAM Metallopeptidase With T	3.03
LIPC	Lipase C, Hepatic Type	3.03
ROS1	ROS Proto-Oncogene 1, Recepto	3.03
NOS1	Nitric Oxide Synthase 1	3
F8	Coagulation Factor VIII	3
CYBA	Cytochrome B-245 Alpha Chain	3
ADM	Adrenomedullin	3
PLAU	Plasminogen Activator, Urokina	2.98
PLAUR	Plasminogen Activator, Urokina	2.98
ICAM1	Intercellular Adhesion Molecule	2.92
CAT	Catalase	2.92
RETN	Resistin	2.92
SOD3	Superoxide Dismutase 3	2.92
LDLR	Low Density Lipoprotein Recep	2.86
TBXAS1	Thromboxane A Synthase 1	2.86
FABP2	Fatty Acid Binding Protein 2	2.86
IGHE	Immunoglobulin Heavy Constan	2.86
MMP14	Matrix Metallopeptidase 14	2.82
MMP3	Matrix Metallopeptidase 3	2.82
CYP3A5	Cytochrome P450 Family 3 Subf	2.8
ANG	Angiogenin	2.8
PDX1	Pancreatic And Duodenal Home	2.8
PAFAH1B1	Platelet Activating Factor Acety	2.8
APOC2	Apolipoprotein C2	2.8
HABP2	Hyaluronan Binding Protein 2	2.8
APOA2	Apolipoprotein A2	2.8
LGALS2	Galectin 2	2.8
PRDX1	Peroxiredoxin 1	2.73
HTR1A	5-Hydroxytryptamine Receptor 1	2.73
LBR	Lamin B Receptor	2.73
PROCR	Protein C Receptor	2.73
LTA	Lymphotoxin Alpha	2.73
OSBPL9	Oxysterol Binding Protein Like 9	2.73
S100B	S100 Calcium Binding Protein B	2.7
EGF	Epidermal Growth Factor	2.7
G6PD	Glucose-6-Phosphate Dehydroge	2.7
SLC9A1	Solute Carrier Family 9 Member	2.65
NPR2	Natriuretic Peptide Receptor 2	2.65
F13A1	Coagulation Factor XIII A Chain	2.65
TIMP3	TIMP Metallopeptidase Inhibito	2.65
ITGA9	Integrin Subunit Alpha 9	2.65
SAA1	Serum Amyloid A1	2.65
CHKA	Choline Kinase Alpha	2.65
BRINP3	BMP/Retinoic Acid Inducible N	2.65
PTEN	Phosphatase And Tensin Homol	2.55
CASP8	Caspase 8	2.55
NFKB1	Nuclear Factor Kappa B Subunit	2.55
MYD88	Myeloid Differentiation Primary	2.55

CD40	CD40 Molecule	2.55
CD8A	CD8a Molecule	2.55
ITGA2	Integrin Subunit Alpha 2	2.55
AVP	Arginine Vasopressin	2.55
GP1BA	Glycoprotein Ib Platelet Subunit	2.55
ADH1B	Alcohol Dehydrogenase 1B (Cla	2.55
MB	Myoglobin	2.55
PIK3C2A	Phosphatidylinositol-4-Phosphat	2.55
HFE	Homeostatic Iron Regulator	2.55
MIR16-1	MicroRNA 16-1	2.55
ADAM10	ADAM Metallopeptidase Domai	2.43
ITGB2	Integrin Subunit Beta 2	2.43
COMT	Catechol-O-Methyltransferase	2.43
NR3C1	Nuclear Receptor Subfamily 3 G	2.43
NGF	Nerve Growth Factor	2.43
CASP1	Caspase 1	2.43
CACNA1B	Calcium Voltage-Gated Channel	2.43
EDNRA	Endothelin Receptor Type A	2.43
CTGF	Connective Tissue Growth Facto	2.43
HIF1A	Hypoxia Inducible Factor 1 Subu	2.43
PTGIR	Prostaglandin I2 Receptor	2.43
CYP1A1	Cytochrome P450 Family 1 Subf	2.43
RHOA	Ras Homolog Family Member A	2.43
LCAT	Lecithin-Cholesterol Acyltransfe	2.43
SCARB1	Scavenger Receptor Class B Me	2.43
P2RY2	Purinergic Receptor P2Y2	2.43
FABP3	Fatty Acid Binding Protein 3	2.43
LBP	Lipopolysaccharide Binding Pro	2.43
CYP4F2	Cytochrome P450 Family 4 Subf	2.43
SOCS3	Suppressor Of Cytokine Signalin	2.43
CD34	CD34 Molecule	2.43
RAMP1	Receptor Activity Modifying Pro	2.43
CALCRL	Calcitonin Receptor Like Recept	2.43
LIPG	Lipase G, Endothelial Type	2.43
NTN1	Netrin 1	2.43
ADIPOR2	Adiponectin Receptor 2	2.43
IL16	Interleukin 16	2.43
CCL11	C-C Motif Chemokine Ligand 1	2.43
CYP4A11	Cytochrome P450 Family 4 Subf	2.43
RHOB	Ras Homolog Family Member B	2.43
SERPINE2	Serpin Family E Member 2	2.43
GSTM1	Glutathione S-Transferase Mu 1	2.43
ZFHX3	Zinc Finger Homeobox 3	2.43
RHOD	Ras Homolog Family Member D	2.43
DEFA1	Defensin Alpha 1	2.43
SMTN	Smoothelin	2.43
IL1RAPL2	Interleukin 1 Receptor Accessory	2.43
GSTT1	Glutathione S-Transferase Theta	2.43

MIR146A	MicroRNA 146a	2.43
MIR491	MicroRNA 491	2.43
SNHG14	Small Nucleolar RNA Host Gen	2.43
PROC	Protein C, Inactivator Of Coagul	2.4
DGKZ	Diacylglycerol Kinase Zeta	2.13
TRPM4	Transient Receptor Potential Cat	2.13
CALM1	Calmodulin 1	2.13
DGKB	Diacylglycerol Kinase Beta	2.13
DGKQ	Diacylglycerol Kinase Theta	2.13
MIR26B	MicroRNA 26b	2.13
ENG	Endoglin	2.02
HTRA1	HtrA Serine Peptidase 1	1.85
CASP7	Caspase 7	1.78
HLA-DRB1	Major Histocompatibility Comp	1.78
APEX1	Apurinic/Apyrimidinic Endodeo	1.78
LOC107372315	OSGEP/APEX1 Bi-Directional I	1.78
GFAP	Glial Fibrillary Acidic Protein	1.73
COL3A1	Collagen Type III Alpha 1 Chair	1.72
TGFBR3	Transforming Growth Factor Be	1.72
ANGPTL6	Angiopoietin Like 6	1.72
INS	Insulin	1.56
HP	Haptoglobin	1.5
APOM	Apolipoprotein M	1.47
CD40LG	CD40 Ligand	1.45
NPPA	Natriuretic Peptide A	1.44
FTMT	Ferritin Mitochondrial	1.44
PTGER2	Prostaglandin E Receptor 2	1.43
TP53	Tumor Protein P53	1.35
FAS	Fas Cell Surface Death Receptor	1.35
PROS1	Protein S	1.35
S100A8	S100 Calcium Binding Protein A	1.33
HRG	Histidine Rich Glycoprotein	1.25
PTGER3	Prostaglandin E Receptor 3	1.22
PRNP	Prion Protein	1.22
KCNN4	Potassium Calcium-Activated Cl	1.22
PTGER4	Prostaglandin E Receptor 4	1.22
IGFBP3	Insulin Like Growth Factor Binc	1.22
PDGFA	Platelet Derived Growth Factor S	1.22
PKD1	Polycystin 1, Transient Receptor	1.17
THBS1	Thrombospondin 1	1.17
BMP6	Bone Morphogenetic Protein 6	1.17
S100A9	S100 Calcium Binding Protein A	1.17
MPZ	Myelin Protein Zero	1.17
UCP3	Uncoupling Protein 3	1.17
KIAA0319L	KIAA0319 Like	1.17
TLR3	Toll Like Receptor 3	1.08
TTR	Transthyretin	1.08
SOD2	Superoxide Dismutase 2	1.08

BIRC5	Baculoviral IAP Repeat Contain	1.08
ALDOA	Aldolase, Fructose-Bisphosphate	1.08
ANXA5	Annexin A5	1.08
SELE	Selectin E	1.08
ALDOB	Aldolase, Fructose-Bisphosphate	1.08
MDK	Midkine	1.08
PON2	Paraoxonase 2	1.08
IL18R1	Interleukin 18 Receptor 1	1.08
CSF3	Colony Stimulating Factor 3	1.08
MIRLET7C	MicroRNA Let-7c	1.08
MIR196A2	MicroRNA 196a-2	1.08
