# Supplementary material for: Mechanism of Radix Rhei Et Rhizome Intervention in Cerebral Infarction: A Research Based on Chemoinformatics and Systematic Pharmacology
Source: Evid Based Complement Alternat Med. 2021 Sep 6;2021:6789835. doi: 10.1155/2021/6789835 (PMC8440083; doi:10.1155/2021/6789835)
Supplement: Supplementary Materials — Table S1: potential targets for potential compounds; Table S2: proteomics data; Table S3: CI gene; Table S4: enrichment analysis of clusters based on gene ontology (GO) annotation of Radix Rhei Et Rhizome-CI PPI network; Table S5: pathway enrichment analysis of Radix Rhei Et Rhizome-CI PPI network; Table S6: reactome pathways of Radix Rhei Et Rhizome-CI PPI network; and Table S7: the biological processes, signaling pathways, and reactome of proteomics proteins' PPI network. [file 6789835.f1.zip › 6789835.f1/Table S4.pdf]

Table S4 Enrichment analysis of clusters based on Gene Ontology (GO) annotation

Cluster	Term	Pathway	Count	%	Pvalue
	GO:0045429	positive regulation of nitric oxide bios	10	0.095356	6.45E-14
	GO:0031663	lipopolysaccharide-mediated signaling	9	0.085821	3.59E-13
	GO:0048661	positive regulation of smooth muscle	10	0.095356	1.60E-12
	GO:0071260	cellular response to mechanical stimul	10	0.095356	7.81E-12
	GO:0045944	positive regulation of transcription fro	23	0.219319	9.00E-12
	GO:0008217	regulation of blood pressure	9	0.085821	1.55E-10
	GO:0006954	inflammatory response	15	0.143034	1.87E-10
	GO:0043066	negative regulation of apoptotic proce	16	0.15257	1.88E-10
	GO:0002576	platelet degranulation	10	0.095356	2.39E-10
	GO:0070374	positive regulation of ERK1 and ERK	11	0.104892	1.49E-09
	GO:0051092	positive regulation of NF-kappaB tran	10	0.095356	2.36E-09
	GO:0008284	positive regulation of cell proliferatio	15	0.143034	2.74E-09
	GO:0043491	protein kinase B signaling	7	0.066749	2.98E-09
	GO:0008285	negative regulation of cell proliferatio	14	0.133499	3.78E-09
	GO:0010628	positive regulation of gene expression	12	0.114427	5.44E-09
	GO:0000165	MAPK cascade	12	0.114427	5.44E-09
	GO:0014068	positive regulation of phosphatidylino	8	0.076285	6.08E-09
	GO:0071222	cellular response to lipopolysaccharid	9	0.085821	1.36E-08
	GO:0050729	positive regulation of inflammatory re	8	0.076285	1.39E-08
	GO:0032496	response to lipopolysaccharide	10	0.095356	1.49E-08
	GO:0001525	angiogenesis	11	0.104892	1.54E-08
	GO:0045766	positive regulation of angiogenesis	9	0.085821	1.56E-08
	GO:0001666	response to hypoxia	10	0.095356	2.26E-08
	GO:0006935	chemotaxis	9	0.085821	2.49E-08
	GO:0043524	negative regulation of neuron apoptoti	9	0.085821	4.61E-08
	GO:0022617	extracellular matrix disassembly	7	0.066749	5.15E-07
1	GO:0032355	response to estradiol	7	0.066749	1.50E-06
	GO:0007159	leukocyte cell-cell adhesion	5	0.047678	2.59E-06
	GO:0034612	response to tumor necrosis factor	5	0.047678	2.59E-06
	GO:0043406	positive regulation of MAP kinase act	6	0.057214	3.42E-06
	GO:0048015	phosphatidylinositol-mediated signali	7	0.066749	3.67E-06
	GO:0032930	positive regulation of superoxide anio	4	0.038142	4.79E-06
	GO:0045080	positive regulation of chemokine bios	4	0.038142	6.82E-06
	GO:0035690	cellular response to drug	6	0.057214	7.44E-06
	GO:0050900	leukocyte migration	7	0.066749	8.27E-06
	GO:0071347	cellular response to interleukin-1	6	0.057214	8.57E-06
	GO:0043537	negative regulation of blood vessel en	4	0.038142	2.05E-05
	GO:0006928	movement of cell or subcellular comp	6	0.057214	2.19E-05
	GO:0071456	cellular response to hypoxia	6	0.057214	3.73E-05
	GO:0071356	cellular response to tumor necrosis fac	6	0.057214	7.16E-05
	GO:0042346	positive regulation of NF-kappaB imp	4	0.038142	7.33E-05
	GO:0050679	positive regulation of epithelial cell p	5	0.047678	9.00E-05
	GO:0032733	positive regulation of interleukin-10 p	4	0.038142	9.71E-05
	GO:0007166	cell surface receptor signaling pathwa	8	0.076285	9.77E-05

GO:0044267	cellular protein metabolic process	6	0.057214	9.99E-05
GO:0050727	regulation of inflammatory response	5	0.047678	1.09E-04
GO:0070371	ERK1 and ERK2 cascade	4	0.038142	1.11E-04
GO:0042327	positive regulation of phosphorylation	4	0.038142	1.25E-04
GO:0030593	neutrophil chemotaxis	5	0.047678	1.31E-04
GO:1901215	negative regulation of neuron death	3	0.028607	0.010779
GO:0060020	Bergmann glial cell differentiation	2	0.019071	0.034831
GO:1901214	regulation of neuron death	2	0.019071	0.064787
GO:0051968	positive regulation of synaptic transm	2	0.019071	0.072132
GO:0002576	platelet degranulation	17	0.132543	1.47E-20
GO:0070374	positive regulation of ERK1 and ERK	18	0.14034	3.21E-18
GO:0043066	negative regulation of apoptotic proce	23	0.179323	6.61E-17
GO:0030335	positive regulation of cell migration	16	0.124747	5.31E-15
GO:0001934	positive regulation of protein phospho	13	0.101357	5.88E-13
GO:0010628	positive regulation of gene expression	16	0.124747	9.62E-13
GO:0042730	fibrinolysis	8	0.062373	3.91E-12
GO:0030168	platelet activation	12	0.09356	5.05E-12
GO:0007165	signal transduction	27	0.21051	5.50E-12
GO:0043406	positive regulation of MAP kinase act	10	0.077967	6.52E-12
GO:0043065	positive regulation of apoptotic proces	16	0.124747	6.76E-12
GO:0001666	response to hypoxia	13	0.101357	2.19E-11
GO:0048661	positive regulation of smooth muscle	9	0.07017	3.16E-10
GO:0042127	regulation of cell proliferation	12	0.09356	8.82E-10
GO:0008284	positive regulation of cell proliferatio	16	0.124747	3.13E-09
GO:0043410	positive regulation of MAPK cascade	9	0.07017	3.67E-09
GO:0014068	positive regulation of phosphatidylino	8	0.062373	1.99E-08
GO:0001938	positive regulation of endothelial cell	8	0.062373	3.04E-08
GO:0048015	phosphatidylinositol-mediated signali	9	0.07017	3.14E-08
GO:0033138	positive regulation of peptidyl-serine	8	0.062373	3.36E-08
GO:0048010	vascular endothelial growth factor rec	8	0.062373	4.10E-08
GO:0045766	positive regulation of angiogenesis	9	0.07017	5.95E-08
GO:0014066	regulation of phosphatidylinositol 3-k	8	0.062373	7.19E-08
GO:0051781	positive regulation of cell division	7	0.054577	7.62E-08
GO:0001525	angiogenesis	11	0.085763	7.98E-08
GO:0006954	inflammatory response	13	0.101357	1.67E-07
GO:2000352	negative regulation of endothelial cell	6	0.04678	1.72E-07
GO:0007596	blood coagulation	10	0.077967	1.78E-07
GO:0007173	epidermal growth factor receptor sign	7	0.054577	2.23E-07
GO:0043552	positive regulation of phosphatidylino	6	0.04678	2.94E-07
GO:0000187	activation of MAPK activity	8	0.062373	6.37E-07
GO:0043154	negative regulation of cysteine-type e	7	0.054577	7.85E-07
GO:0045429	positive regulation of nitric oxide bios	6	0.04678	1.59E-06
GO:0042060	wound healing	7	0.054577	1.89E-06
GO:0051897	positive regulation of protein kinase B	7	0.054577	2.52E-06
GO:0050715	positive regulation of cytokine secreti	5	0.038983	5.06E-06

	GO:0090277	positive regulation of peptide hormon	4	0.031187	5.31E-06
	GO:0071456	cellular response to hypoxia	7	0.054577	5.50E-06
	GO:0043525	positive regulation of neuron apoptoti	5	0.038983	4.63E-05
	GO:0048011	neurotrophin TRK receptor signaling	4	0.031187	5.17E-05
	GO:0007179	transforming growth factor beta recep	6	0.04678	6.81E-05
	GO:0001935	endothelial cell proliferation	4	0.031187	7.48E-05
	GO:0014911	positive regulation of smooth muscle	4	0.031187	1.04E-04
	GO:0048146	positive regulation of fibroblast prolif	5	0.038983	1.14E-04
	GO:0072378	blood coagulation, fibrin clot formatio	3	0.02339	1.27E-04
	GO:0035791	platelet-derived growth factor recepto	3	0.02339	2.11E-04
	GO:0048008	platelet-derived growth factor recepto	4	0.031187	3.23E-04
	GO:2000379	positive regulation of reactive oxygen	4	0.031187	3.58E-04
	GO:0032287	peripheral nervous system myelin mai	3	0.02339	4.41E-04
	GO:0008286	insulin receptor signaling pathway	5	0.038983	4.74E-04
	GO:0097192	extrinsic apoptotic signaling pathway	4	0.031187	5.20E-04
	GO:0006919	activation of cysteine-type endopeptid	5	0.038983	5.99E-04
	GO:0060020	Bergmann glial cell differentiation	3	0.02339	7.51E-04
	GO:0031639	plasminogen activation	3	0.02339	7.51E-04
	GO:1901216	positive regulation of neuron death	3	0.02339	0.002769
	GO:1901215	negative regulation of neuron death	3	0.02339	0.014823
	GO:0099565	chemical synaptic transmission, posts	2	0.015593	0.023013
	GO:0042632	cholesterol homeostasis	5	0.136277	2.69E-06
	GO:0019433	triglyceride catabolic process	4	0.109022	7.41E-06
	GO:0042157	lipoprotein metabolic process	4	0.109022	2.68E-05
	GO:0017187	peptidyl-glutamic acid carboxylation	3	0.081766	1.26E-04
	GO:0007584	response to nutrient	4	0.109022	1.99E-04
	GO:0034375	high-density lipoprotein particle remo	3	0.081766	2.39E-04
	GO:0043691	reverse cholesterol transport	3	0.081766	3.47E-04
	GO:0019915	lipid storage	3	0.081766	6.23E-04
	GO:0006465	signal peptide processing	3	0.081766	6.77E-04
	GO:0070328	triglyceride homeostasis	3	0.081766	7.32E-04
	GO:0044267	cellular protein metabolic process	4	0.109022	7.82E-04
	GO:0006629	lipid metabolic process	4	0.109022	0.00178
	GO:0006687	glycosphingolipid metabolic process	3	0.081766	0.002191
	GO:0001523	retinoid metabolic process	3	0.081766	0.003988
	GO:0043627	response to estrogen	3	0.081766	0.004516
**3**	GO:0006869	lipid transport	3	0.081766	0.006124
	GO:0051345	positive regulation of hydrolase activi	2	0.054511	0.00618
	GO:0006508	proteolysis	5	0.136277	0.006901
	GO:0006689	ganglioside catabolic process	2	0.054511	0.009256
	GO:0070508	cholesterol import	2	0.054511	0.009256
	GO:0034384	high-density lipoprotein particle clear	2	0.054511	0.009256
	GO:0010886	positive regulation of cholesterol stor	2	0.054511	0.01079
	GO:0043534	blood vessel endothelial cell migratio	2	0.054511	0.01538
	GO:0061045	negative regulation of wound healing	2	0.054511	0.016906

	GO:0009313	oligosaccharide catabolic process	2	0.054511	0.016906
	GO:0006888	ER to Golgi vesicle-mediated transpo	3	0.081766	0.025246
	GO:0001935	endothelial cell proliferation	2	0.054511	0.02752
	GO:0006401	RNA catabolic process	2	0.054511	0.032036
	GO:0007596	blood coagulation	3	0.081766	0.032669
	GO:0006656	phosphatidylcholine biosynthetic proc	2	0.054511	0.038025
	GO:0043691	reverse cholesterol transport	5	0.095166	4.20E-08
	GO:0042632	cholesterol homeostasis	6	0.114199	1.75E-07
	GO:0042157	lipoprotein metabolic process	5	0.095166	9.85E-07
	GO:0033344	cholesterol efflux	4	0.076132	1.69E-05
	GO:0006468	protein phosphorylation	8	0.152265	2.96E-05
	GO:0038083	peptidyl-tyrosine autophosphorylation	4	0.076132	7.12E-05
	GO:0034372	very-low-density lipoprotein particle r	3	0.057099	8.30E-05
**4**	GO:0034374	low-density lipoprotein particle remod	3	0.057099	2.16E-04
	GO:0001523	retinoid metabolic process	4	0.076132	2.52E-04
	GO:0008203	cholesterol metabolic process	4	0.076132	3.47E-04
	GO:0045579	positive regulation of B cell differenti	3	0.057099	3.57E-04
	GO:0034375	high-density lipoprotein particle remo	3	0.057099	4.11E-04
	GO:0007596	blood coagulation	5	0.095166	5.00E-04
	GO:0007597	blood coagulation, intrinsic pathway	3	0.057099	5.97E-04
	GO:0006954	inflammatory response	6	0.114199	9.26E-04
	GO:0007169	transmembrane receptor protein tyrosi	4	0.076132	9.53E-04
	GO:0008202	steroid metabolic process	3	0.078288	0.001566
	GO:0006231	dTMP biosynthetic process	2	0.052192	0.004104
	GO:0007584	response to nutrient	3	0.078288	0.004565
	GO:0045329	carnitine biosynthetic process	2	0.052192	0.006831
	GO:0042493	response to drug	4	0.104384	0.007956
**5**	GO:0006563	L-serine metabolic process	2	0.052192	0.008191
	GO:0009308	amine metabolic process	2	0.052192	0.010907
	GO:0009812	flavonoid metabolic process	2	0.052192	0.010907
	GO:0046653	tetrahydrofolate metabolic process	2	0.052192	0.013617
	GO:0035999	tetrahydrofolate interconversion	2	0.052192	0.013617
	GO:0032872	regulation of stress-activated MAPK c	2	0.060551	0.009491
	GO:0051056	regulation of small GTPase mediated	3	0.090827	0.010931
	GO:0021954	central nervous system neuron develo	2	0.060551	0.014204
**6**	GO:0055114	oxidation-reduction process	4	0.121102	0.031803
	GO:0048813	dendrite morphogenesis	2	0.060551	0.043182
	GO:0000302	response to reactive oxygen species	2	0.060551	0.045465
	GO:0006687	glycosphingolipid metabolic process	2	0.060551	0.052283
**8**	GO:0043401	steroid hormone mediated signaling p	4	0.601504	3.71E-08
	GO:0006048	UDP-N-acetylglucosamine biosynthet	3	0.307062	2.34E-06

9	GO:0006046	N-acetylglucosamine catabolic proces	2	0.204708	7.14E-04
	GO:0019262	N-acetylneuraminate catabolic process	2	0.204708	0.001429
	GO:0019388	galactose catabolic process	2	0.204708	0.001667
10	GO:0038083	peptidyl-tyrosine autophosphorylation	5	0.09984	1.07E-06
	GO:0030168	platelet activation	5	0.09984	7.33E-05
	GO:0071560	cellular response to transforming grov	4	0.079872	1.20E-04
	GO:0051918	negative regulation of fibrinolysis	3	0.059904	1.67E-04
	GO:0018108	peptidyl-tyrosine phosphorylation	5	0.09984	2.21E-04
	GO:0006468	protein phosphorylation	7	0.139776	2.30E-04
	GO:0008284	positive regulation of cell proliferation	7	0.139776	2.59E-04
	GO:0016032	viral process	6	0.119808	2.73E-04
	GO:0001938	positive regulation of endothelial cell	4	0.079872	3.32E-04
	GO:0030335	positive regulation of cell migration	5	0.09984	4.45E-04
	GO:0031295	T cell costimulation	4	0.079872	4.76E-04

l

Genes	Fold Enrichment	Bonferroni
EGFR, ICAM1, PTGS2, AGT, EDN1, ESR1, IL1B, TLR4, JA	58.2853176	1.09E-10
MAPK1, CCL2, MYD88, MAPK14, IL1B, TLR4, NOS3, CC	70.48880597	6.03E-10
EGFR, MYD88, PTGS2, HMOX1, EDN1, IGF1, THBS1, CC	41.77114428	2.70E-09
EGFR, MYD88, PTGS2, AGT, CASP8, MMP7, IL1B, TLR4,	35.29955855	1.31E-08
EGFR, CSF3, IL4, AR, HRAS, CREB1, PPARG, EDN1, ESR	5.87606311	1.52E-08
ACE, PTGS2, REN, AGT, HMOX1, EDN1, PPARG, NOS3,	34.7021814	2.62E-07
SELP, CCL2, PTGS2, CRP, CXCL8, TLR4, CD40, CCL5, IL	9.91926909	3.15E-07
IL4, EGFR, MMP9, IGF1, BCL2L1, ANXA5, PTEN, IL10, S	8.813252419	3.16E-07
SELP, VWF, APP, ALB, SERPINE1, IGF1, HGF, THBS1, T	24.33270541	4.02E-07
EGFR, ICAM1, HRAS, CCL2, MAP2K1, CTGF, TLR4, CCL	15.7536887	2.51E-06
ICAM1, AR, MYD88, CD40LG, AGT, IL1B, TLR4, CAT, C	18.84412524	3.97E-06
CSF3, EGFR, AR, HRAS, EDN1, IGF1, HGF, BCL2L1, PTE	8.06738838	4.61E-06
CCL2, IGF1, IL1B, CD40, CCL5, PTEN, TGFB1	53.16327454	5.01E-06
HRAS, AR, PTGS2, MAP2K1, CXCL8, PTEN, IL10, TGFB1	8.860545756	6.36E-06
CDC42, AR, HRAS, HIF1A, MAP2K1, CTGF, MAPK14, CR	11.47909308	9.16E-06
EGFR, MAPK1, HRAS, CCL2, MAP2K1, GRB2, IL1B, JAK	11.47909308	9.16E-06
CSF3, SELP, AGT, IGF1, JAK2, CAT, HGF, CCL5	30.84638347	1.02E-05
CSF3, ICAM1, CCL2, MAPK14, SERPINE1, CXCL8, TLR4,	19.96143178	2.29E-05
EGFR, CCL2, AGT, SERPINE1, TLR4, JAK2, CCL5, IL2	27.46595788	2.34E-05
SELP, PTGS2, REN, CASP8, EDN1, TLR4, JAK2, CD40, SE	15.28212596	2.51E-05
HIF1A, CCL2, PTGS2, CTGF, MAPK14, HMOX1, SERPINI	12.36276019	2.59E-05
HIF1A, HMOX1, SERPINE1, CXCL8, IL1B, NOS3, HGF, T	19.61427644	2.63E-05
HIF1A, CCL2, HMOX1, CREB1, CAT, THBS1, ADIPOQ, M	14.5713294	3.80E-05
IL4, MAPK1, HRAS, CCL2, MAP2K1, MAPK14, CXCL8, C	18.48886714	4.19E-05
HRAS, BDNF, CCL2, APOE, HMOX1, JAK2, BCL2L1, NG	17.08819539	7.76E-05
MMP9, MMP7, MMP3, MMP13, MMP2, PLG, MMP1	23.08405342	8.67E-04
PTGS2, CTGF, CASP8, ESR1, CAT, PTEN, TGFB1	19.27898967	0.002521397
ICAM1, SELP, CD40LG, CCL5, SELE	50.12537313	0.004344983
PTGS2, CASP8, JAK2, ADIPOQ, SELE	50.12537313	0.004344983
EGFR, HRAS, EDN1, CD40, FGF2, TGFB1	25.48747786	0.005733552
EGFR, GRB2, RHOA, IGF1, PTEN, FGF2, NGF	16.55083075	0.006151773
EGFR, AGT, CRP, TGFB1	111.3897181	0.008029368
IL4, MYD88, HMOX1, IL1B	100.2507463	0.011418756
EGFR, CCL2, REN, EDN1, IL1B, ADIPOQ	21.79364049	0.012445111
ICAM1, SELP, HRAS, GRB2, MMP9, SELE, MMP1	14.38023	0.013825678
ICAM1, HIF1A, CCL2, EDN1, CXCL8, CCL5	21.17973513	0.014317239
APOE, THBS1, FGF2, TGFB1	71.60767591	0.033859834
PTGS2, MAP2K1, MAPK14, CXCL8, IGF1, JAK2	17.48559528	0.036191627
ICAM1, HIF1A, PTGS2, HMOX1, EDN1, PTEN	15.6641791	0.060842856
ICAM1, CCL2, EDN1, CXCL8, THBS1, CCL5	13.67055631	0.113513067
PTGS2, RHOA, IL1B, TLR4	47.7384506	0.116099777
EGFR, HRAS, IGF1, CCL5, TGFB1	20.88557214	0.140491699
IL4, CD40LG, TLR4, HGF	43.58728099	0.150759032
EGFR, HRAS, CCL2, MYD88, AGT, MAPK14, CASP8, ED	7.31757272	0.151572707

APP, IGF1, MMP13, MMP2, PLG, MMP1	12.74373893	0.154760158
MYD88, PTGS2, ESR1, JAK2, SELE	19.89102109	0.16748925
MAPK1, MAP2K1, AGT, IGF1	41.77114428	0.169920733
EGFR, AR, THBS1, CCL5	40.10029851	0.190256195
CCL2, EDN1, CXCL8, IL1B, CCL5	18.98688376	0.197356608
CSF3, APOE, CREB1	18.79701493	0.999999988
MAPK1, MAP2K1	55.69485904	1
APOE, CCL5	29.48551361	1
EGFR, PTGS2	26.38177533	1

ALDOA, PDGFB, F13A1, F8, SOD1, TGFB2, FGG, F5, FGA	35.08221703	2.38E-17
HMGB1, BMP2, IL6, TNF, PDGFB, SRC, KDR, PTPN11, M	21.86300181	5.18E-15
IL6, XIAP, SOCS3, AIF1, TP53, RAF1, NFKB1, SRC, KDR,	10.74463764	1.79E-13
BMP2, PDGFB, AIF1, KIT, MMP14, KDR, IGF1R, PTK2, IN	18.48321409	8.60E-12
AKT1, PTK2, BMP2, TNF, AIF1, VEGFA, TEK, ADAM17,	21.75779926	9.48E-10
BMP2, IL6, TNF, LDLR, PDGFB, TP53, TLR3, KIT, TGFB2	12.98057783	1.55E-09
FGG, FGA, FGB, SERPINF2, HRG, PROS1, PLAU, PLAUR	80.97408077	6.31E-09
AKT1, FGG, IL6, F5, FGA, FGB, F8, RAF1, PIK3CA, HRG,	22.17985691	8.16E-09
PGF, TLR3, NFKB1, NR3C1, KIT, SRC, PGR, AKT1, IGF1I	4.943185163	8.87E-09
TNF, PDGFB, VEGFA, ELANE, PDGFRB, KIT, FGF1, EGF	36.02660373	1.05E-08
HMGB1, BMP2, IL6, TNF, TP53, TLR3, SOD1, SRC, AKT1	11.33637131	1.09E-08
BMP2, PGF, RAF1, MMP14, TGFB2, CASP3, TEK, VEGFA	16.06535178	3.54E-08
AKT1, RETN, IL6, TNF, PDGFB, AIF1, SERPINF2, ELANE	31.8835443	5.10E-07
AGTR1, PTK2, TNF, XIAP, PPBP, FAS, KIT, NOS2, ABL1,	13.78747862	1.42E-06
IL6, PDGFB, PGF, KIT, KDR, TGFB2, IGF1R, PTK2, S100E	7.298093117	5.06E-06
HMGB1, BMP2, IL6, PDGFB, INS, FAS, KIT, IGFBP3, KDI	23.61744023	5.93E-06
PTK2, PDGFB, INS, TEK, PDGFRB, KIT, KDR, TGFB2	26.16085686	3.21E-05
AKT1, BMP2, PDGFB, PGF, F3, VEGFA, TEK, KDR	24.64428545	4.90E-05
AKT1, IGF1R, PDGFB, PDGFRB, PIK3CA, KIT, FGF1, EGI	18.04728923	5.07E-05
AKT1, IL6, TNF, GSK3B, VEGFA, RAF1, PIK3CA, MIF	24.29222423	5.42E-05
PTK2, HSP90AA1, NCF1, PGF, VEGFA, PIK3CA, SRC, KD	23.61744023	6.62E-05
CD34, PGF, F3, VEGFA, TEK, FGF1, ENG, IL1A, KDR	16.63489268	9.61E-05
AKT1, PDGFB, PDGFRB, PIK3CA, KIT, FGF1, EGF, PTPN	21.80071405	1.16E-04
PPBP, PDGFB, PGF, VEGFA, FGF1, IL1A, TGFB2	31.65741988	1.23E-04
PTK2, PGF, VEGFA, TEK, PIK3CA, HRG, MMP14, FGF1, I	10.48487257	1.29E-04
AKT1, HMGB1, BMP2, IL6, TNF, PPBP, AIF1, TLR3, NFK	7.290872048	2.69E-04
FGG, FGA, FGB, TEK, ABL1, KDR	45.54792043	2.77E-04
FGG, F5, FGA, FGB, F3, F13A1, F8, PROS1, PLAU, PLAUI	11.55200881	2.88E-04
PTK2, ADAM17, PIK3CA, ABL1, EGF, SRC, PTPN11	26.56962025	3.60E-04
PTK2, PDGFB, TEK, PDGFRB, KIT, SRC	41.14005717	4.74E-04
BMP2, TNF, MAPK10, KIT, FGF1, SOD1, EGF, PTPN11	15.89210931	0.001028289
AKT1, IL6, XIAP, VEGFA, RAF1, MDM2, SRC	21.56374977	0.001266074
AKT1, IL6, TNF, HSP90AA1, AIF1, INS	29.65911098	0.002566705
CASP3, IL6, INS, PDGFRB, RAF1, ENG, TGFB2	18.59873418	0.003045201
PTK2, IL6, TNF, INS, F3, TEK, SRC	17.71308017	0.004057013
TNF, INS, SRC, IL1A, MIF	42.51139241	0.008132856

FGG, FGA, FGB, INS	106.278481	0.008533307
AKT1, S100B, VEGFA, TP53, MDM2, CCNA2, SRC	15.49894515	0.008832321
CASP3, TP53, ABL1, NQO1, TGFB2	24.71592582	0.072046786
CASP3, RAF1, SRC, PTPN11	53.13924051	0.080048474
PTK2, PDGFB, PARP1, ENG, SRC, TGFB2	13.86241057	0.104140038
HMGB1, CD34, TEK, MMP14	47.23488045	0.113758461
RETN, PDGFB, PDGFRB, SRC	42.51139241	0.154302669
PDGFB, PDGFRB, ABL1, CCNA2, MIF	19.68120019	0.168356312
FGG, FGA, FGB	159.4177215	0.185395068
PDGFRB, PTPN1, ABL1	127.5341772	0.288754125
PDGFB, PDGFRB, SRC, PTPN11	29.31820166	0.406261177
AGTR1, PDGFB, TP53, PDGFRB	28.34092827	0.438598021
AKT1, SOD1, AKT2	91.09584087	0.509005585
AKT1, IGF1R, INS, PTPN1, AKT2	13.62544628	0.534413324
CASP3, GSK3B, FAS, IL1A	25.00670141	0.56815302
CASP3, TNF, F3, FAS, CASP1	12.80463627	0.620074503
GFAP, ABL1, PTPN11	70.85232068	0.702476833
FGG, FGA, FGB	70.85232068	0.702476833
GSK3B, ABL1, PARP1	37.51005212	0.988611639
AKT1, IL6, CD34	15.94177215	1
AKT1, GSK3B	85.02278481	1
LPL, APOA1, LIPG, FABP4, SCARB1	48.58796296	8.18E-04
LPL, APOA1, FABP4, FABP5	99.50814815	0.002250035
LPL, APOA1, LPA, SCARB1	65.46588694	0.008119359
PROZ, F7, PROC	169.6161616	0.037497296
APOA1, HMGCR, LIPG, ARSA	33.61761762	0.058590704
APOA1, LIPG, SCARB1	124.3851852	0.070110042
APOA1, LIPG, SCARB1	103.654321	0.100235139
APOA1, GM2A, HEXB	77.74074074	0.172606005
PROZ, F7, PROC	74.63111111	0.185971484
LPL, APOA1, SCARB1	71.76068376	0.199645633
APOA1, APCS, HEXB, CST3	21.08223478	0.211563575
LPL, LPA, LIPG, FABP5	15.84524652	0.418216609
GM2A, HEXB, ARSA	41.4617284	0.486609844
LPL, RBP4, APOA1	30.58652095	0.703268707
APOA1, ARSA, F7	28.7042735	0.747421784
APOA1, LPA, GM2A	24.5497076	0.845470457
APOA1, GM2A	310.962963	0.848084212
CTSL, CTSK, LPA, PROC, HABP2	6.219259259	0.878181199
GM2A, HEXB	207.308642	0.940798708
APOA1, SCARB1	207.308642	0.940798708
APOA1, SCARB1	207.308642	0.940798708
LPL, SCARB1	177.6931217	0.963044675
APOA1, SCARB1	124.3851852	0.99101245
APCS, HMGCR	113.0774411	0.994390317

GM2A, HEXB	113.0774411	0.994390317
PROZ, F7, PROC	11.66111111	0.99957921
APOA1, SCARB1	69.10288066	0.99979314
RNASE2, RNASE3	59.23104056	0.999949738
PROZ, F7, PROC	10.14009662	0.999958812
APOA1, FABP5	49.75407407	0.999992382
LCAT, APOC2, ABCA1, LIPC, APOM	133.2698413	1.99E-05
APOB, LCAT, APOC2, ABCA1, LIPC, APOM	44.97857143	8.29E-05
APOB, LCAT, APOC2, ABCA1, APOM	63.12781955	4.66E-04
APOB, APOC2, ABCA1, APOM	76.76342857	0.007971403
PIK3CG, BRAF, HCK, ZAP70, AURKA, CDK6, SYK, BTK	8.417042607	0.01391834
HCK, ZAP70, SYK, BTK	47.97714286	0.03311349
LCAT, APOC2, LIPC	205.6163265	0.038516862
APOB, PLA2G7, LIPC	130.8467532	0.09729276
TTR, APOB, APOC2, APOM	31.46042155	0.112338846
APOB, LCAT, ABCA1, LIPC	28.22184874	0.151553054
ZAP70, SYK, BTK	102.8081633	0.155256406
LCAT, LIPC, APOM	95.95428571	0.176702494
F11, F10, SERPINC1, SERPINA1, SERPIND1	13.03726708	0.210819927
F11, F12, F10	79.96190476	0.245932037
PIK3CG, ITGAL, OLR1, HCK, ZAP70, SYK	7.595326046	0.354767226
HCK, ZAP70, SYK, BTK	19.99047619	0.362958791
AKR1C3, CYP1A1, SULT1A1	48.81395349	0.346066704
TYMS, SHMT1	466.4444444	0.671886508
AKR1C3, AHCY, UCP3	28.36486486	0.710623155
SHMT1, ACADM	279.8666667	0.843927854
TYMS, CYP1A1, HADH, PNP	9.206140351	0.885227079
SHMT1, PSPH	233.2222222	0.892362988
CYP1A1, SULT1A1	174.9166667	0.948807631
CYP1A1, SULT1A1	174.9166667	0.948807631
TYMS, SHMT1	139.9333333	0.975654968
TYMS, SHMT1	139.9333333	0.975654968
PRDX1, GSTP1	199.9047619	0.921614468
RAC2, RHOB, RHOD	17.90191898	0.946853257
NPY, MAP2	133.2698413	0.978064068
G6PD, CRYZ, PRDX1, GLRX	5.402831403	0.999821199
MAP2, DCX	43.22265122	0.999992389
PRDX1, GSTP1	41.00610501	0.999995978
PSAP, ASAH1	35.53862434	0.999999407
RARG, THRB, RXRB, RARB	294.5964912	1.74E-06
UAP1, GNPDA1, GNPDA2	915.9272727	3.51E-05

GNPDA1, GNPDA2	2238.933333	0.010664037
GNPDA1, GNPDA2	1119.466667	0.021216228
GALK1, GALE	959.5428571	0.02470897
ERBB4, LCK, JAK3, CSK, INSR	61.73529412	7.92E-04
LCK, F2, RAC1, PF4, PIK3R1	21.47314578	0.052572395
ARG1, TGFBR1, FBN1, APAF1	40.31692677	0.084503972
THBD, F2, APOH	148.1647059	0.115740974
FGFR2, ERBB4, JAK3, INSR, EPHA2	16.13994617	0.150149861
CCL11, ADAM10, TGFBR1, LCK, JAK3, CSK, PIK3R1	7.581527348	0.156239602
FGFR2, ADAM10, ERBB4, TGFBR1, F2, INSR, CDK2	7.418833628	0.173838016
APOA2, EIF4E, LCK, PIK3R1, HSPA8, EPHA2	9.910682668	0.182220305
CCL11, CYBA, ARG1, TGFBR1	28.63086104	0.216882916
CCL11, ADAM10, TGFBR1, INSR, PIK3R1	13.42071611	0.279843127
LCK, RAC1, CSK, PIK3R1	25.32730015	0.295843031
