# Supplementary material for: Mechanism of Radix Rhei Et Rhizome Intervention in Cerebral Infarction: A Research Based on Chemoinformatics and Systematic Pharmacology
Source: Evid Based Complement Alternat Med. 2021 Sep 6;2021:6789835. doi: 10.1155/2021/6789835 (PMC8440083; doi:10.1155/2021/6789835)
Supplement: Supplementary Materials — Table S1: potential targets for potential compounds; Table S2: proteomics data; Table S3: CI gene; Table S4: enrichment analysis of clusters based on gene ontology (GO) annotation of Radix Rhei Et Rhizome-CI PPI network; Table S5: pathway enrichment analysis of Radix Rhei Et Rhizome-CI PPI network; Table S6: reactome pathways of Radix Rhei Et Rhizome-CI PPI network; and Table S7: the biological processes, signaling pathways, and reactome of proteomics proteins' PPI network. [file 6789835.f1.zip › 6789835.f1/Table S5.pdf]

Table S5 Pathway enrichment analysis

Term	Pathway	Count	%	Pvalue
hsa04610	Complement and coagulation cascade	32	0.032486	7.90E-18
hsa04014	Ras signaling pathway	50	0.050759	2.27E-12
hsa04151	PI3K-Akt signaling pathway	64	0.064971	4.90E-12
hsa04668	TNF signaling pathway	32	0.032486	1.21E-11
hsa04068	FoxO signaling pathway	36	0.036546	1.49E-11
hsa04066	HIF-1 signaling pathway	29	0.02944	1.03E-10
hsa04015	Rap1 signaling pathway	45	0.045683	1.07E-10
hsa03320	PPAR signaling pathway	23	0.023349	8.85E-10
hsa04370	VEGF signaling pathway	21	0.021319	5.39E-09
hsa04722	Neurotrophin signaling pathway	30	0.030455	6.17E-09
hsa04660	T cell receptor signaling pathway	27	0.02741	7.37E-09
hsa04915	Estrogen signaling pathway	26	0.026395	2.73E-08
hsa04931	Insulin resistance	27	0.02741	4.16E-08
hsa04620	Toll-like receptor signaling pathway	26	0.026395	1.17E-07
hsa04010	MAPK signaling pathway	44	0.044668	1.32E-07
hsa04062	Chemokine signaling pathway	34	0.034516	1.50E-06
hsa04611	Platelet activation	27	0.02741	2.01E-06
hsa04064	NF-kappa B signaling pathway	21	0.021319	3.36E-06
hsa04920	Adipocytokine signaling pathway	17	0.017258	3.46E-05

Genes	Fold Enrichment	Bonferroni
F13A1, C1R, C1S, FGG, FGA, FGB, SERPINE1, SERPINC1	6.292427751	2.13E-15
HRAS, PDGFB, PGF, PDGFA, NFKB1, AKT1, CDC42, RH	3.001780384	6.12E-10
HSP90AB1, HRAS, PDGFB, PGF, PDGFA, NFKB1, TLR4,	2.5169711	1.32E-09
CCL2, TNF, PTGS2, MMP9, EDN1, NFKB1, MMP3, CCL5,	4.057733783	3.28E-09
HRAS, GRB2, PTEN, IL10, TGFB1, TGFB2, AKT1, IGF1R,	3.645147046	4.02E-09
EDN1, HK1, NFKB1, TLR4, PDHB, AKT1, IGF1R, INS, HM	4.098680966	2.79E-08
FGFR2, ITGAL, FGFR1, HRAS, PDGFB, PDGFA, PGF, IT	2.907438715	2.88E-08
LPL, PPARA, PPARD, ACADM, OLR1, RXRB, RXRA, PP	4.657687892	2.39E-07
PIK3CG, HRAS, MAP2K1, PTGS2, RAF1, MAPKAPK2, SR	4.670967116	1.45E-06
HRAS, GRB2, NFKB1, MAPKAPK2, AKT1, CDC42, BDN	3.392011834	1.67E-06
IL4, PIK3CG, ITK, HRAS, TNF, CD8A, MAP2K1, GRB2, R	3.663372781	1.99E-06
HSP90AB1, HRAS, GRB2, MMP9, HSPA1A, MMP2, SRC,	3.563325563	7.37E-06
PPARA, TNF, NFKB1, PTEN, AKT1, NR1H2, PDPK1, INS,	3.392011834	1.12E-05
TNF, CXCL8, TLR3, TLR4, NFKB1, CCL5, AKT1, MYD88	3.328011611	3.17E-05
FGFR2, FGFR1, HRAS, TNF, PDGFB, GRB2, PDGFA, NFK	2.359660406	3.57E-05
HRAS, CCL2, GRB2, CXCL8, NFKB1, PF4, CCL5, SRC, A	2.480180696	4.06E-04
COL3A1, SRC, BTK, AKT1, FGG, PTGIR, GP6, FGA, FGB,	2.817979062	5.44E-04
ICAM1, TNF, XIAP, PTGS2, CXCL8, NFKB1, TLR4, BCL2	3.275045909	9.06E-04
PPARA, TNF, RXRB, SOCS3, RXRA, ADIPOR2, NFKB1,	3.29509721	0.009305837
