# Supplementary material for: Mechanism of Radix Rhei Et Rhizome Intervention in Cerebral Infarction: A Research Based on Chemoinformatics and Systematic Pharmacology
Source: Evid Based Complement Alternat Med. 2021 Sep 6;2021:6789835. doi: 10.1155/2021/6789835 (PMC8440083; doi:10.1155/2021/6789835)
Supplement: Supplementary Materials — Table S1: potential targets for potential compounds; Table S2: proteomics data; Table S3: CI gene; Table S4: enrichment analysis of clusters based on gene ontology (GO) annotation of Radix Rhei Et Rhizome-CI PPI network; Table S5: pathway enrichment analysis of Radix Rhei Et Rhizome-CI PPI network; Table S6: reactome pathways of Radix Rhei Et Rhizome-CI PPI network; and Table S7: the biological processes, signaling pathways, and reactome of proteomics proteins' PPI network. [file 6789835.f1.zip › 6789835.f1/Table S6.pdf]

Table S6 Reactome pathways

Pathway ide	Pathway name	Entities ratio	PValue	FDR	Genes
R-HSA-38328	Nuclear Receptor tra	0.005958567	1.11E-16	3.45E-14	RARG;THRB;THRA;N
R-HSA-67858	Interleukin-4 and Int	0.014619275	1.11E-16	3.45E-14	SMARCAL1;NOTCH3
R-HSA-76002	Platelet activation, si	0.0203007	1.11E-16	3.45E-14	APP;SERPINA1;DGK
R-HSA-12802	Cytokine Signaling i	0.087369223	1.11E-16	3.45E-14	APP;SMARCAL1;CSF
R-HSA-44914	Signaling by Interleu	0.04427354	1.11E-16	3.45E-14	APP;SMARCAL1;CSF
R-HSA-16825	Immune System	0.195524146	1.11E-16	3.45E-14	APP;CDA;SMARCAL
R-HSA-11460	Platelet degranulatio	0.009492136	3.18E-14	8.48E-12	CFD;APP;CPB2;SERP
R-HSA-76005	Response to elevatec	0.009977136	4.34E-14	1.01E-11	CFD;APP;CPB2;SERP
R-HSA-10958	Hemostasis	0.056883531	5.93E-14	1.23E-11	APP;SERPINE2;DGKI
R-HSA-90069	Signaling by Recepto	0.038384258	6.44E-13	1.09E-10	THRB;NCF1;PDE3B;I
R-HSA-19941	Negative regulation (	0.009284279	3.88E-12	6.01E-10	SRC;PTEN;PDGFB;PI
R-HSA-14087	Formation of Fibrin (	0.003810712	2.38E-11	3.40E-09	FGB;FGA;SERPINE2;
R-HSA-68115	PI5P, PP2A and IER	0.008729994	3.54E-11	4.71E-09	SRC;PDGFB;PDGFA;
R-HSA-67980	Neutrophil degranula	0.033257119	8.44E-11	1.05E-08	GPI;CDA;HSP90AB1;
R-HSA-12576	PIP3 activates AKT	0.02189427	2.32E-10	2.69E-08	GSK3B;THRB;PTEN;
R-HSA-90069	Intracellular signalin	0.025081411	1.33E-09	1.45E-07	GSK3B;THRB;PTEN;
R-HSA-56632	Diseases of signal tra	0.03436569	1.89E-09	1.95E-07	GSK3B;THRB;PTEN;
R-HSA-15923	Activation of Matrix	0.002424998	2.19E-09	2.14E-07	MMP7;CMA1;MMP1;
R-HSA-22195	Constitutive Signalir	0.007136423	2.31E-09	2.15E-07	PDGFRB;OSBPL9;SR
R-HSA-90069	Signaling by Nuclea	0.026674981	2.82E-09	2.51E-07	RARG;HSP90AB1;CC
R-HSA-14087	Common Pathway of	0.00256357	5.10E-09	4.28E-07	FGB;FGA;SERPINE2;
R-HSA-38884	Costimulation by the	0.006720709	3.19E-08	2.59E-06	PDPK1;SRC;PTPN11;
R-HSA-21243	Generic Transcriptio	0.107600637	7.26E-08	5.59E-06	CCNT1;SERPINE1;PL
R-HSA-40902	SUMOylation of intr	0.002771427	9.07E-08	6.71E-06	GSK3B;THRB;THRA;
R-HSA-96072	FLT3 Signaling	0.021547842	1.48E-07	1.04E-05	THRB;PDE3B;CTSV;I
R-HSA-16824	Innate Immune Syste	0.092011363	1.51E-07	1.04E-05	APP;CDA;SMARCAL
R-HSA-16889	Toll-like Receptor C	0.012748562	2.49E-07	1.64E-05	APP;THRB;ITGB2;PL
R-HSA-89505	Gene and protein exp	0.005057854	4.56E-07	2.92E-05	IL10;ARF1;FECH;GST
R-HSA-73857	RNA Polymerase II T	0.117231345	8.00E-07	4.96E-05	CCNT1;SERPINE1;PL
R-HSA-90205	Interleukin-12 signal	0.005819996	1.26E-06	7.56E-05	IL10;ARF1;FECH;GST
R-HSA-14742	Extracellular matrix	0.022794984	1.77E-06	1.03E-04	APP;SERPINE1;ITGB
R-HSA-67837	Interleukin-10 signal	0.005958567	1.85E-06	1.04E-04	IL10;IL1A;CSF3;IL1R
R-HSA-56849	MAPK1/MAPK3 sig	0.020577842	2.01E-06	1.08E-04	THRB;SRC;PDGFB;PI
R-HSA-38994	PD-1 signaling	0.003117855	2.12E-06	1.08E-04	LCK;CSK;PTPN11;HI
R-HSA-56830	MAPK family signal	0.024111411	2.13E-06	1.08E-04	THRB;CTSV;FGF1;FC
R-HSA-14335	Signaling by SCF-KI	0.003533569	2.42E-06	1.21E-04	CMA1;STAT1;SRC;PT
R-HSA-12363	Signaling by ERBB4	0.005681425	2.93E-06	1.43E-04	THRB;SRC;EGF;PIK3
R-HSA-44711	Interleukin-12 family	0.006651424	3.46E-06	1.63E-04	IL10;ARF1;FECH;GST
R-HSA-20243	Translocation of ZA	0.002909998	4.14E-06	1.87E-04	ZAP70;LCK;HLA-DR
R-HSA-20243	Generation of secon	0.004087854	4.15E-06	1.87E-04	ITK;ZAP70;LCK;BTK
R-HSA-56730	RAF/MAP kinase ca	0.020092843	4.85E-06	2.01E-04	THRB;SRC;PDGFB;PI
R-HSA-17482	Plasma lipoprotein a	0.006789995	4.86E-06	2.01E-04	ABCA1;SCARB1;NR1
R-HSA-74751	Insulin receptor sign	0.004988568	4.92E-06	2.01E-04	OSBPL9;PDPK1;INSR
R-HSA-90244	NR1H2 and NR1H3-	0.005889282	5.11E-06	2.01E-04	ABCA1;C1R;NR1H2;N
R-HSA-14742	Degradation of the e	0.010254278	5.16E-06	2.01E-04	HTRA1;PLG;CTSV;CT
R-HSA-38142	Regulation of Insulir	0.008799279	5.79E-06	2.20E-04	APP;SERPINA1;SERP
R-HSA-19413	Signaling by VEGF	0.009353565	6.45E-06	2.45E-04	NCF1;SRC;PIK3R1;TY
R-HSA-35419	Integrin signaling	0.002702141	8.09E-06	2.91E-04	FGB;PTPN1;FGA;SYI
R-HSA-20242	Phosphorylation of C	0.003117855	9.27E-06	3.24E-04	LCK;CSK;HLA-DRB1
R-HSA-14424	Collagen degradatior	0.004780711	9.47E-06	3.31E-04	MMP7;MME;MMP1;N
R-HSA-12519	Nuclear signaling by	0.003256426	1.53E-05	5.20E-04	ADAM17;ERBB4;SRC
R-HSA-76009	Platelet Aggregation	0.00367214	1.58E-05	5.20E-04	FGB;PTPN1;FGA;SYI
R-HSA-74160	Gene expression (Tr	0.12817848	1.68E-05	5.53E-04	CCNT1;SERPINE1;PL
R-HSA-14083	Intrinsic Pathway of	0.002217141	2.31E-05	7.40E-04	F7;F8;PROC;F10;SER

R-HSA-9615	FOXO-mediated tra	0.003394998	2.45E-05	7.85E-04	GSK3B;NPY;CAT;PL
R-HSA-7520	Dissolution of Fibri	9.70E-04	3.03E-05	9.39E-04	SERPINE2;PLAU;SE
R-HSA-9009	Extra-nuclear estrog	0.007621423	3.07E-05	9.50E-04	SRC;PIK3R1;EGFR;I
R-HSA-2428	IRS-related events tr	0.004780711	3.22E-05	9.65E-04	OSBPL9;PDPK1;PDE
R-HSA-1114	SMAC (DIABLO) b	4.85E-04	3.33E-05	9.65E-04	CASP7;APAF1;CASP
R-HSA-1114	SMAC(DIABLO)-m	4.85E-04	3.33E-05	9.65E-04	CASP7;APAF1;CASP
R-HSA-4420	VEGFA-VEGFR2 P	0.008729994	3.66E-05	0.001063	NCF1;SRC;PIK3R1;T
R-HSA-1989	PPARA activates ge	0.012055706	4.47E-05	0.001252	ABCA1;NR1H2;CYP4
R-HSA-1227	Signaling by ERBB2	0.005404282	4.87E-05	0.001363	HSP90AA1;THRB;SR
R-HSA-2107	Regulation of gene e	0.002424998	5.41E-05	0.001462	PKLR;AKT2;HNF4G;
R-HSA-2428	IGF1R signaling cas	0.004988568	5.50E-05	0.001486	OSBPL9;PDPK1;PDE
R-HSA-4002	Regulation of lipid m	0.012194277	5.51E-05	0.001489	ABCA1;NR1H2;CYP4
R-HSA-2404	Signaling by Type 1	0.005057854	6.53E-05	0.001656	OSBPL9;PDPK1;PDE
R-HSA-1779	Signaling by EGFR	0.00415714	6.72E-05	0.001656	THRB;SRC;EGF;PDG
R-HSA-1114	SMAC, XIAP-regula	5.54E-04	6.91E-05	0.001656	CASP7;APAF1;CASP
R-HSA-1114	Activation of caspas	5.54E-04	6.91E-05	0.001656	CASP7;APAF1;CASP
R-HSA-8863	Downregulation of E	0.002494284	7.04E-05	0.001656	HSP90AA1;THRB;ER
R-HSA-6802	Signaling by RAS m	0.003741426	7.20E-05	0.001656	FGB;FGA;MAP2K1;V
R-HSA-9649	Signaling downstrea	0.003741426	7.20E-05	0.001656	FGB;FGA;MAP2K1;V
R-HSA-6802	Signaling by modera	0.003741426	7.20E-05	0.001656	FGB;FGA;MAP2K1;V
R-HSA-8963	Plasma lipoprotein r	0.003741426	7.20E-05	0.001656	LIPC;APOC2;ALB;LI
R-HSA-2024	TCR signaling	0.010184993	7.32E-05	0.001683	ITK;PDPK1;WAS;PT
R-HSA-5686	Regulation of TLR b	0.002147856	8.11E-05	0.001866	FGB;FGA;PSAP;FGG;
R-HSA-6802	Paradoxical activatic	0.003810712	8.79E-05	0.001997	FGB;FGA;MAP2K1;V
R-HSA-1867	Downstream signal t	0.00256357	9.08E-05	0.001997	PDGFRB;PIK3CA;GM
R-HSA-1146	GPVI-mediated acti	0.002979284	9.41E-05	0.00207	SYK;PDPK1;PTPN11;
R-HSA-5674	Constitutive Signali	0.002217141	1.07E-04	0.00235	NOTCH3;GSK3B;CR
R-HSA-8963	Chylomicron remod	0.001177856	1.15E-04	0.002417	APOC2;APOA2;LPL;
R-HSA-1867	Signaling by PDGF	0.004849997	1.23E-04	0.002581	PDGFRB;STAT1;SRC
R-HSA-1597	Transport of gamma-	6.24E-04	1.31E-04	0.002743	F7;PROC;F10;PROS1;
R-HSA-3893	CD28 co-stimulation	0.002702141	1.47E-04	0.003087	CDC42;PIK3CA;PDP
R-HSA-1097	PI3K Cascade	0.004018569	1.55E-04	0.003105	OSBPL9;PIK3CA;PD
R-HSA-1123	IRS-mediated signal	0.004503568	1.65E-04	0.003307	OSBPL9;PDPK1;PDE
R-HSA-9029	NR1H3 & NR1H2 re	0.004572854	1.96E-04	0.003717	RXRB;ABCA1;RXRA
R-HSA-1597	Removal of aminote	6.93E-04	2.29E-04	0.004354	F7;PROC;F10;PROS1;
R-HSA-5654	Negative regulation	0.002840712	2.31E-04	0.004382	OSBPL9;THRB;SRC;
R-HSA-1625	Signal Transduction	0.228989122	2.60E-04	0.004934	APP;SERPINE1;EDN
R-HSA-7475	Signaling by Insulin	0.006720709	2.78E-04	0.005144	OSBPL9;PDPK1;INS
R-HSA-1876	Signalling to ERKs	0.002909998	2.86E-04	0.005144	MAP2K1;GM2A;SRC;

MAOB;NR1I3;NR1I2;HNF4G;RORA;NR3C1;NR3C2;RXRB;RXRA;VDR;NR1H2;NR1H4;NR1H3;ESR
3;CXCL8;CCL11;MAOA;ITGB2;F13A1;RORA;PIK3R1;PTGS2;TNF;HIF1A;FGF2;ICAM1;SOCS3;PIM
B;PROS1;SERPINE1;F13A1;PIK3CG;AKT2;RAC2;TIMP3;AKT1;RAC1;CTSF;PRKACA;LGALS7;FG
F3;IL1RN;PITPNA;F13A1;PLAT;RORA;FGF1;TNF;FGF2;ICAM1;AKT2;PIM1;AKT1;PRKACA;TNFR
F3;IL1RN;PITPNA;F13A1;PLAT;RORA;TNF;FGF2;ICAM1;PIM1;AKT1;PRKACA;MAP2K1;HGF;TA
1;IL1RN;PITPNA;ICAM2;ICAM1;IGLV2-8;LGALS3;PNP;AKT2;AKT1;PRKACA;ARSA;MIF;RNASE
PINA1;PROS1;SERPINE1;PDGFB;F13A1;PDGFA;PLG;THBS1;APOM;AKT2;APOH;PSAP;TIMP3;CT
PINA1;PROS1;SERPINE1;PDGFB;F13A1;PDGFA;PLG;THBS1;APOM;AKT2;APOH;PSAP;TIMP3;CT
B;PROS1;SERPINE1;F13A1;PLAT;IGLV2-8;PLAU;AKT2;BSG;CHEK1;AKT1;PRKACA;LGALS7;GU
PLAT;FGF1;FGF2;IGF1R;STS;GM2A;CASP3;AKT2;CHEK1;KDR;AKT1;RAC1;JAK2;PRKACA;HRA
DGFA;PIK3R1;FGF1;FGF2;EGFR;INS;ERBB4;AKT2;RAC2;MAPK1;AKT1;RAC1;PDGFRB;OSBPL9
SERPIND1;F10;VWF;SERPINC1;PROS1;F12;FGG;F11;F13A1;GP1BA;F2;F3;F5;THBD;F7;PROCR;F
PIK3R1;FGF1;FGF2;EGFR;INS;ERBB4;RAC2;MAPK1;AKT1;RAC1;PDGFRB;OSBPL9;EGF;HGF;IN
SERPINA1;HEXB;ITGB2;HP;HMGB1;PYGL;ITGAL;CTSS;FCAR;ALAD;ADAMTS4;LGALS3;PNP;A
CTSV;FGF1;FGF2;AKT2;RAC2;AKT1;RAC1;PDGFRB;CSNK2A1;PDPK1;HGF;TAP1;CREB1;PIK3C
CTSV;FGF1;FGF2;AKT2;RAC2;AKT1;RAC1;PRKACA;PDGFRB;CSNK2A1;PDPK1;HGF;TAP1;CRE
PLAT;CTSV;FGF1;FGF2;AKT2;RAC2;AKT1;RAC1;JAK2;LBR;HRAS;FGB;PDGFRB;FGA;MAP2K1
MMP2;MMP3;F11;PLG;CTSV;MMP8;MMP9;MMP14;STS;MMP13;MMP16;CTSL;CTSK;CTSG;ELA
C;EGF;HGF;PDGFB;PDGFA;PTPN11;PIK3R1;FGF1;ESR1;FGF2;EGFR;ESR2;PIK3CA;ERBB4;LCK;
NT1;MIR26B;F13A1;IGF1R;AKT2;AKT1;CTSD;HRAS;PDK2;HSP90AA1;MMP7;PDPK1;MMP1;MM
SERPIND1;F10;SERPINC1;PROS1;FGG;F13A1;F2;F5;THBD;F7;PROCR;F8;PROC;HLA-DRB1;PF4

LAT;RORA;NR3C1;CTGF;NR3C2;NPPB;LGALS3;LIPC;AKT2;CHEK1;NPPA;AKT1;PRKACA;G6PD
;VDR;NR1H2;NR1I2;NR1H4;NR1H3;RORA;NR3C1;ESR1;NR3C2;AR;RXRA;RARA;PGR;PPARG;PP
FGF1;FGF2;AKT2;JAK2;JAK3;HRAS;FGB;PDGFRB;FGA;MAP2K1;VWF;PDPK1;HGF;FGG;TAP1;I
1;NCF1;PROS1;ICAM2;HP;PLAT;IGLV2-8;LGALS3;PNP;PLAU;PRKACA;ARSA;MAP2K1;TAP1;A
AT;HMGB1;CTSV;CTSS;EEA1;MAPK8;CASP8;CTSL;CTSK;PSAP;MAPK1;LBP;APOB;CTSB;FGB

LAT;RORA;NR3C1;CTGF;NR3C2;NPPB;LGALS3;LIPC;AKT2;CHEK1;NPPA;AKT1;PRKACA;G6PD

2;ICAM2;CTSV;ITGAL;FGF2;CTSS;ICAM1;ADAMTS4;STS;CTSL;CASP3;CTSK;BSG;KDR;CTSG;I

DGFA;CTSV;FGF1;FGF2;EGFR;ERBB4;AKT2;MAPK1;CSK;JAK2;JAK3;HRAS;PDGFRB;FGB;OSB

GF2;AKT2;RAC1;JAK2;PRKACA;JAK3;HRAS;FGB;PDGFRB;FGA;MAP2K1;VWF;MMP2;HGF;FGG
TPN11;PIK3R1;MMP9;PIK3CA;LCK;CHEK1;KIT;BTK;GRB2;RAC1;JAK2;PRKACA;HRAS

DGFA;CTSV;FGF1;FGF2;EGFR;ERBB4;AKT2;MAPK1;CSK;JAK2;JAK3;HRAS;PDGFRB;FGB;OSB
1H2;APOA2;APOA1;NR1H3;LPL;LCAT;APOA5;ACAT1;LIPC;APOC2;FABP7;ALB;LIPG;APOE;LPA
R;PDE3B;PTPN11;PIK3R1;FGF1;FGF2;INS;PIK3CA;AKT2;MAPK1;GRB2;HRAS;FGFR2;FGFR1

TSS;ADAMTS4;STS;CTSL;CTSK;CASP3;BSG;CTSG;CTSD;ELANE;CTSB;MMP7;MME;CMA1;MM
PINC1;PLG;CST3;MAP2;CTSG;APOE;JAK2;APOB;FGA;SERPIND1;CMA1;MMP1;MMP2;IGFBP3;M
YMS;CDC42;AKT2;KDR;CTNNA1;AKT1;RAC1;PRKACA;HRAS;CBR1;HSP90AA1;PDPK1;NOS3;A

MMP2;MMP3;ADAM10;CTSV;MMP8;MMP9;MMP12;ADAM17;COL3A1;MMP14;STS;MMP13;CTS

LAT;RORA;NR3C1;CTGF;NR3C2;NPPB;LGALS3;LIPC;AKT2;CHEK1;NPPA;AKT1;PRKACA;G6PD

GF1R;AKT2;MAPK1;AKT1;HRAS;HSP90AA1;MMP7;PDPK1;NOS3;MMP1;EGF;MMP2;MMP3;ADA
3B;PTPN11;PIK3R1;IGF1;FGF1;FGF2;IGF1R;PIK3CA;AKT2;GRB2;HRAS;FGFR2;FGFR1

YMS;CDC42;AKT2;KDR;CTNNA1;AKT1;RAC1;PRKACA;HRAS;HSP90AA1;PDPK1;NOS3;ADAM
A11;APOA2;NR1H4;APOA1;NR1H3;RORA;HMGCR;APOA5;AGT;RXRB;FABP1;RXRA;CYP1A1;
C;EGF;ADAM33;PDGFA;PLG;PIK3R1;EGFR;RHOA;PIK3CA;ERBB4;AKT2;AKT1;GRB2;PRKACA

3B;PTPN11;PIK3R1;IGF1;FGF1;FGF2;IGF1R;PIK3CA;AKT2;GRB2;HRAS;FGFR2;FGFR1
A11;APOA2;NR1H4;APOA1;NR1H3;RORA;HMGCR;APOA5;AGT;RXRB;FABP1;RXRA;CYP1A1;
3B;PTPN11;PIK3R1;IGF1;FGF1;FGF2;IGF1R;PIK3CA;AKT2;GRB2;HRAS;FGFR2;FGFR1

N;TAP1;PLAT;PIK3R1;CTSV;NFKB1;ZAP70;PIK3CA;LCK;BTK;CSK;PRKCQ;HLA-DRB1

;PDGFB;PDGFA;PTPN11;PLAT;PLG;PIK3R1;THBS1;COL3A1;PIK3CA;GM2A;CASP3;GRB2;HRA

A;STS;PNP;AKT2;CHEK1;KDR;AKT1;PRKACA;PDK2;PRKCH;CSNK2A1;MIF;ACE2;AR;RBP4;M
;PDE3B;PTPN11;PIK3R1;FGF1;FGF2;INS;PIK3CA;AKT2;MAPK1;GRB2;HRAS;FGFR2;FGFR1

И1;AKT1;CCL2;HMOX1;LBP;JAK2;JAK3;IL10;OSBPL9;HSPA8;HSP90AA1;TGFB1;NOS2;MMP1;S
GB;FGA;CBR1;PRKCH;SYK;VWF;PDPK1;HGF;SERPINF2;FGG;ANXA5;APOA1;GP1BA;F2;DGKZ;
RSF4;PDGFRB;MAP2K1;HGF;ADIPOQ;TAP1;MIF;ADAM17;MTAP;MAPKAPK2;RAF1;PPIA;TP53;
P1;MIF;MTAP;MAPKAPK2;PPIA;TP53;NOTCH3;FECH;MAOA;PDGFA;PIK3R1;HIF1A;SOCS3;TT
E3;RNASE2;CHIT1;MTAP;BTK;PRKCQ;TP53;CFB;CES1;CFD;MAOA;PDGFB;PDGFA;PIK3R1;HIF
SF;LGALS7;FGB;FGA;CBR1;TGFB2;TGFB1;VWF;EGF;HGF;SERPINF2;FGG;ANXA5;APOA1;PPB
SF;PRKACA;LGALS7;FGB;FGA;CBR1;TGFB2;TGFB1;VWF;EGF;HGF;SERPINF2;FGG;ANXA5;A
JCY1A1;PRKCH;HGF;SERPINF2;MIF;DGKZ;PROCR;DGKQ;PRKCQ;RAF1;TP53;PPIA;CFD;CPB2;
AS;CTSB;PDGFRB;CBR1;MAP2K1;HSP90AA1;PDPK1;HGF;ITGA2;ADAM10;CYBA;NGF;MMP9;P
;EGF;HGF;INSR;PTPN11;ESR1;ESR2;GALE;PIK3CA;LCK;KIT;GRB2;MET;MYD88;FGFR2;FGFR1

ATIC;GM2A;PLAU;OLR1;CTSG;RAC1;CTSD;CTSB;ARSA;HSP90AA1;MME;APAF1;ARG1;PLAU
CA;LCK;KIT;BLVRB;PPARG;TP53;MET;NOTCH3;FECH;MAOB;C1R;SRC;PDGFB;XIAP;PDGFA;P
EB1;PIK3CA;LCK;KIT;BLVRB;PPARG;AVP;TP53;MET;NOTCH3;FECH;MAOB;C1R;SRC;PDGFB;
;HSP90AA1;VWF;PDPK1;HGF;FGG;ADAM10;TAP1;TGFBR1;TGFBR2;KAT2B;RBP4;ADAM17;C

ИP2;MMP3;MMP9;KAT2B;CREB1;PIK3CA;RARA;RARB;BLVRB;PGR;PPARD;CRABP2;ADH1C;C

;CSNK2A1;IGFBP3;TAP1;AR;DPEP1;PGR;PRKCQ;TP53;NOTCH3;FECH;MAOB;PDGFB;RTN4;SO

DUSP6;HCK;PIK3CA;KIT;RAF1;MET;SRC;PDGFB;PDGFA;PIK3R1;EGFR;ERBB4;MAPK1;CSK;OS
DAM10;CYBA;MIF;RNASE3;RNASE2;CHIT1;MAPKAPK2;BTK;PRKCQ;RAF1;PPIA;S100A9;CFB;
;FGA;MAP2K1;FGG;PTPN11;S100B;MAPK14;DUSP6;NFKB1;MAPK10;CREB1;MAPKAPK2;BTK;

;CSNK2A1;IGFBP3;TAP1;AR;DDX39B;DPEP1;PGR;PRKCQ;TP53;NOTCH3;PFKFB1;CRABP2;FEC

PRKACA;CTSD;CTSB;FGB;FGA;MMP7;MME;VWF;MMP1;MMP2;ITGA2;FGG;MMP3;ADAM10;M

PL9;FGA;MAP2K1;VWF;EGF;HGF;FGG;TAP1;BRAF;PTPN11;PTK2;IL2;DUSP6;DHFR;GALE;IL6;

G;TAP1;DUSP6;KIT;BLVRB;RAF1;MET;C1R;SRC;PDGFB;PDGFA;EGFR;CDC42;ERBB4;MAPK1;C

PL9;FGA;MAP2K1;VWF;EGF;HGF;FGG;TAP1;BRAF;PTK2;IL2;DUSP6;DHFR;GALE;PPP5C;GDN

ИP1;MMP2;MMP3;F11;ADAM10;MMP8;MMP9;MMP12;COL3A1;MMP14;ADAM17;MMP13;MMP1

ADAM33;CYBA;MAPK14;PTK2;PGF;RHOA;MAPK12;VEGFA;PIK3CA;MAPKAPK2;CALM3;CAL

;CSNK2A1;IGFBP3;TAP1;MIF;AR;DDX39B;DPEP1;PGR;PRKCQ;TP53;NOTCH3;PFKFB1;CRABP2

33;CYBA;MAPK14;PTK2;RHOA;MAPK12;VEGFA;PIK3CA;MAPKAPK2;CALM3;CALM1;CALM2

ITHFD1;BTK;AGTR1;PRKCQ;AGTR2;TP53;CFD;MAOB;CRABP2;SHMT1;HPN;PDGFB;PDGFA;PI

TAT1;MMP2;HGF;MMP3;ADAM33;MMP9;VEGFA;IL4;IL1A;IL6;IL1B;SAA1;LCN2;BIRC5;IGHE;
RHOA;F5;RHOB;F8;PIK3CA;LCK;DGKQ;PRKCQ;ALDOA;RAF1;PPIA;CFD;CPB2;SRC;PDGFB;PD
CES1;NOTCH3;FECH;MAOA;PDGFB;PDGFA;PIK3R1;HIF1A;SOCS3;TTPA;HMOX1;OSBPL9;HSP
PA;HMOX1;OSBPL9;HSPA8;TGFB1;NFKB1;IL2;SMARCA4;IL4;IL6;MDM2;LCN2;GRB2;MYD88;
1A;CD1A;APOM;RNF213;MAP2;TTPA;HMOX1;LTA4H;APOB;ELANE;BRAF;PPBP;LYZ;PTK2;SM
P;IGF1;RHOA;SOD1;F5;VEGFA;SELP;F8;FABP7;ALB;CALM3;ALDOA;CALM1;HRG;CALM2;PP
POA1;PPBP;IGF1;RHOA;SOD1;F5;VEGFA;SELP;F8;FABP7;ALB;CALM3;ALDOA;CALM1;HRG;C
PDGFB;PDGFA;UAP1;PIK3R1;ABO;APOM;TBXA2R;PRDX1;APOH;PSAP;ABL1;APOB;TRPM4;T
GF;DUSP6;RHOA;ADAM17;CREB1;PIK3CA;LCK;MAPKAPK2;KIT;BTK;PGR;MET;SRC;HPN;PD
;NME2;ADAM10;CYBA;MIF;RNASE3;MMP8;RNASE2;MMP9;RHOA;APRT;CHIT1;RAP2A;CAT;
IK3R1;PTGS2;EGFR;INS;ERBB4;MAPK1;OSBPL9;EGF;INSR;PTPN11;ESR1;ESR2;GALE;RHEB;M
XIAP;PDGFA;PIK3R1;PTGS2;EGFR;INS;ERBB4;MAPK1;OSBPL9;EGF;INSR;PTPN11;ESR1;ESR2;
REB1;MMP16;PIK3CA;LCK;KIT;RAF1;MET;NOTCH3;FECH;MAOB;SRC;PDGFB;PDGFA;PIK3R1;
1R;SRC;PLG;PIK3R1;PDHB;EGFR;RXRB;RXRA;ERBB4;MAPK1;APOE;PCK1;ABCA1;NOS3;EGF;
CS3;PRDX1;ABL1;APOE;PCK1;ZFHX3;ST14;TGFB1;VDR;BDNF;ESRRG;SMARCA2;ESR1;NFKB
BPL9;EGF;BRAF;PTPN11;PTK2;IL2;DHFR;GALE;PPP5C;GDNF;GRB2;CALM3;TEK;CALM1;CAL
S100A8;CFD;CPB2;PIK3R1;APOM;TTPA;PSAP;ABL1;MBP;LTA4H;APOB;ELANE;OSBPL9;HSPA
H;MAOB;PDGFB;RTN4;SOCS3;PRDX1;ABL1;APOE;PCK1;ZFHX3;ST14;TGFB1;VDR;BDNF;ESR
MP8;MMP9;MMP12;MMP14;ADAM17;MMP13;MMP16;ITGA9;HTRA1;PDGFB;PDGFA;PLG;THB
PPP5C;GDNF;KIT;GRB2;CALM3;TEK;CALM1;RAF1;CALM2;MET;METAP1;FGFR2;FGFR1
SK;OSBPL9;EGF;BRAF;PTPN11;PTK2;IL2;DHFR;GALE;PPP5C;IL6;GDNF;GRB2;CALM3;TEK;C
;FECH;MAOB;PDGFB;RTN4;SOCS3;PRDX1;ABL1;APOE;PCK1;OSBPL9;ZFHX3;ST14;TGFB1;VD

K3R1;HIF1A;APOM;APOE;PCK1;APOB;KCNN4;ABCA1;INSR;PLK1;BRAF;PPBP;IGF1;PTK2;SMA

GFA;UAP1;PLG;PIK3R1;THBS1;CDC42;APOM;TBXA2R;APOH;PSAP;MAPK1;CSK;P2RY12;PTPN
A8;TGFB1;BRAF;NFKB1;PTK2;IL2;SMARCA4;DHFR;IL4;ISG20;PPP5C;BHMT;IL6;GDNF;TNFSF4
3CL2L1;ARF1;CXCL8;THRB;ITGB2;HMGB1;CTSV;CA1;GM2A;CA2;CASP3;CASP1;CTSG;LBP;JA
IARCA4;DHFR;BST1;PPP5C;BHMT;CD209;TNFSF4;GRB2;KYAT1;CALM3;CALM1;CALM2;HLA-

GFB2;TGFB1;EPHX2;PPBP;IGF1;GP6;SELE;PTK2;SELP;PROC;GIPC1;ALB;CDK2;GRB2;CALM3;H
GFB;PDGFA;PLG;PIK3R1;TYMS;THBS1;EGFR;INS;CDC42;ERBB4;CTNNA1;MAPK1;CSK;APOE;C

BPI;ALDOA;ACPP;PPIA;S100A9;S100A8;CFD;ASAH1;GSTP1;RETN;TYMS;CST3;APOM;TTR;TTP

GALE;RHEB;MDM2;GRB2;CALM3;KYAT1;CALM1;CALM2;MYD88;FGFR2;METAP1;FGFR1
HDAC8;EGFR;RTN4;PAPSS1;TTR;ERBB4;MAPK1;CSK;OSBPL9;TGFB1;STAT1;EGF;ADAM33;BI

AKR1C1;NR1H2;PDX1;AKR1C3;ADAM33;NR1H3;ESR1;PTK2;ESR2;PPP5C;FABP5;FABP6;APOC

l;IL2;ESR2;SMARCA4;CLK1;IL6;BMP2;CDK7;CDK6;RHEB;CDK2;MDM2;CYCS;CALM3;CALM1;

3;PPBP;DEFA1;LYZ;SELE;NFKB1;PTK2;BST1;BHMT;GDNF;CD209;LCN2;KYAT1;GRB2;CALM3;

RG;SMARCA2;ESR1;NFKB1;IL2;ESR2;SMARCA4;CLK1;IL6;BMP2;CDK7;PROC;CDK6;RHEB;CD

R;BDNF;ESRRG;SMARCA2;ESR1;NFKB1;IL2;ESR2;SMARCA4;CLK1;IL6;BMP2;CDK7;PROC;CD

ARCA4;DHFR;PPP5C;GIPC1;GRB2;KYAT1;CALM3;CALM1;CALM2;HLA-DRB1;RARG;ARHGAP1

I1;TGFB2;TGFB1;EGF;PTPN11;PPBP;IGF1;MAPK14;GP6;PTK2;SOD1;VEGFA;SELP;FABP7;GIPC1

4;MDM2;LCN2;GRB2;CALM3;CALM1;CALM2;MYD88;FGFR2;HLA-DRB1;BCL2L1;FGFR1;ARF1;

K2;JAK3;IL10;HSP90AA1;SYK;GSTO1;MMP1;MMP2;MMP3;IL16;MMP9;DUSP6;HCK;IL1A;CREB

-DRB1;GPI;ITK;ARF1;CD40;SERPINA1;HEXB;KIF11;CTSV;PYGL;CTSS;EEA1;C8G;ATIC;CTSL;C

RG;CALM1;CALM2;RAB5A;HLA-DRB1;GALK1;SERPINA1;ITGB2;KIF11;ITGAL;TREM1;PIK3C

SBPL9;PTPN1;NOS3;STAT1;CMA1;BDNF;EGF;INSR;F12;ADAM33;BRAF;PTPN11;IGF1;S100B;M

A;PSAP;MAPK1;CD59;MBP;LTA4H;APOB;ELANE;HSPA8;PKLR;CMA1;ADAM33;PPBP;DEFA1;

AF;PTPN11;ESR1;ESR2;FKBP1A;GALE;MDM2;GRB2;CALM3;KYAT1;CALM1;CALM2;FGFR2;

CALM2;FGFR2;GALK1;GPI;GSK3B;RARG;THRB;THRA;HNF4G;PTEN;CTSV;ITGAL;ALDH2;CT

;CALM1;CALM2;MYD88;BCL2L1;GPI;ITK;HSP90AB1;SERPINA1;THRB;HEXB;ITGB2;HMGB1;C

K2;MDM2;CYCS;GRB2;CALM3;CALM1;CALM2;FGFR2;GALK1;GPI;GSK3B;RARG;THRB;THRA

K6;GDNF;RHEB;CDK2;MDM2;CYCS;GRB2;CALM3;CALM1;CALM2;FGFR2;GALK1;GPI;GSK3B

;ADM;CTSV;RAC2;RAC1;ACADM;JAK2;JAK3;HRAS;CTSD;CTSB;CBR1;EDN1;SYK;PARP1;BAZ

CD40;CXCL8;THRB;PDE3B;ITGB2;CTSV;HMGB1;CA1;GM2A;CA2;CASP3;CASP1;CTSG;LBP;JAI
31;PIK3CA;LCK;IL1B;BIRC5;IGHE;BPI;CCL11;PTGS2;EGFR;CDC42;MAPK8;CCL5;CCL2;MAPK1;
TSK;RAC2;OLR1;CTSG;RAC1;CTSF;JAK2;JAK3;HRAS;CTSD;CD34;CTSB;IL10;TPI1;MME;SYK;C

;THBD;NCS1;RAC2;TIMP3;OLR1;NOS1;RAC1;CTSF;JAK2;HRAS;FGB;FGA;CBR1;PTGIR;SYK;V
IAPK14;ESR1;PTK2;MAPK12;GFAP;VEGFA;COL3A1;GRB2;CALM3;CALM1;CALM2;FGFR2;CDK

MAPK14;LYZ;SELE;NFKB1;BST1;BHMT;FABP5;IMPDH1;CD209;FABP7;QPCT;IMPDH2;LCN2;RA

SL;CTSK;CASP1;ME2;CTSF;LBR;PARP1;APAF1;PDPK1;GP1BA;MMP8;CCNA2;KAT2B;CREB1;M

TSV;PYGL;ITGAL;TREM1;CTSS;FCAR;ALAD;ADAMTS4;EEA1;CASP8;C8G;ATIC;GM2A;CTSL;C

;HNF4G;PTEN;HMGB1;CTSV;ITGAL;ALDH2;CTSL;CTSK;CASP1;ME2;CTSF;LBR;PARP1;APAF1

;RARG;THRB;THRA;HNF4G;PTEN;HMGB1;CTSV;ITGAL;ALDH2;CTSL;CTSK;CASP1;ME2;CTSF

1A;F2;RHOD;RHOA;DUSP6;RHOB;KAT2B;KIT;RARA;RARB;TAX1BP3;PPARG;AVP;PPARD;PAI

K2;JAK3;HRAS;IL10;FGB;FGA;HSP90AA1;SYK;VWF;GSTO1;PDPK1;MMP1;MMP2;FGG;MMP3;II
NOS2;STAT1;CMA1;ADAM33;PTPN11;SOD2;S100B;MAPK14;SOD1;VEGFA;MAPK10;IMPDH1;S.
GSTO1;ARG1;NME2;IL16;F2;RHOA;DUSP6;APRT;HCK;IL1A;RAP2A;IL1B;KIT;IGHE;ALDOA;TLF

WF;PDPK1;MMP1;ITGA2;FGG;MMP3;ANXA5;PLAUR;APOA1;GP1BA;F2;F3;RHOA;F5;RHOB;F7;

MP13;CAT;KIT;RARA;BIRC5;BLVRB;RARB;PPARG;PPARA;MET;PPARD;SLC20A2;C1R;SRC;NF

CTSK;RAC2;PDE4B;CASP1;OLR1;CTSG;NOS1;RAC1;LBP;HRAS;CTSD;CTSB;FGB;FGA;HSP90AA

;PDPK1;GP1BA;MMP8;CCNA2;KAT2B;CREB1;MMP13;CAT;KIT;RARA;BIRC5;BLVRB;RARB;PP

;LBR;HSP90AA1;PARP1;APAF1;PDPK1;GP1BA;MMP8;CCNA2;ACTA2;KAT2B;CREB1;MMP13;C

[illegible]AH1B1;PTGER4;CCL11;SLC20A2;ADH1C;PTGER2;PTGER3;WAS;XIAP;TYMS;PTGS2;EGFR;PA[illegible]

L16;MMP9;DUSP6;HCK;IL1A;CREB1;PIK3CA;LCK;IL1B;KIT;LTA;BIRC5;IGHE;BPI;MET;RNASE

R4;TLR3;APCS;RNASEL;ASAH1;CCL11;GSTP1;WAS;TYMS;PTGS2;EGFR;CST3;RXRB;CD59;PKL

;F8;PIK3CA;LCK;PDE5A;ALDOA;SERPINC1;SRC;PLG;PTGS2;THBS1;CDC42;MAPK1;CSK;P2RY

R1I3;NR1I2;PLG;RETN;HDAC8;THBS1;EGFR;AURKA;INS;RXRB;RXRA;NPY;MAPK1;PTPN1;STA

A1;MME;SYK;APAF1;PDPK1;ARG1;FGG;PLAUR;NME2;F2;MMP8;MMP9;DUSP6;RHOA;APRT;HC

ARG;PPARA;MET;PPARD;SLC20A2;C1R;SRC;NR1I3;NR1I2;PLG;RETN;HDAC8;THBS1;EGFR;AU

AT;KIT;RARA;BIRC5;BLVRB;RARB;PPARG;PPARA;MET;PPARD;SLC20A2;C1R;SRC;NR1I3;NR

PSS1;RXRB;RXRA;HSD17B1;NPY;CTNNA1;LDLR;P2RY12;STAT1;AKR1C1;NR1H2;F12;F11;AKR

L;CCL11;SRC;PTGS2;EGFR;CDC42;MAPK8;ERBB4;CCL5;CCL2;MAPK1;CSK;EIF4E;PTPN1;NOS2

R;STAT1;NR1H4;ADAM33;VEGFA;FKBP1A;COL3A1;CD40LG;FABP5;QPCT;FABP7;SAA1;IL18R

12;PTPN1;NOS2;SERPIND1;F10;NOS3;EGF;F12;F11;NR1H4;PTPN11;MAPK14;SOD1;VEGFA;GAL

AT1;NR1H2;GSR;NR1H4;NR1H3;PTPN11;SOD2;MAPK14;GCK;VEGFA;FAS;METAP1;CDK5R1;PF

CK;RAP2A;CREB1;PIK3CA;LCK;IL1B;CAT;IGHE;BPI;ALDOA;ACPP;TLR4;TLR3;CRP;APCS;ASA

URKA;INS;RXRB;RXRA;NPY;MAPK1;PTPN1;STAT1;NR1H2;GSR;NR1H4;NR1H3;PTPN11;SOD2;

1I2;PLG;RETN;HDAC8;THBS1;EGFR;AURKA;INS;RXRB;RXRA;NPY;MAPK1;PTPN1;STAT1;NR1

1C3;ADAM33;AKR1C2;NR1H3;AGT;VEGFA;FKBP1A;COL3A1;FABP5;FAP;NMNAT1;FABP6;FA[illegible]

;STAT1;CMA1;EGF;ADAM33;PTPN11;S100B;SOD2;MAPK14;VEGFA;SOD1;MAPK10;GALE;CD4

1;RAB9B;HSPA1A;CSF3;NCF1;PROS1;HP;F13A1;PLAT;RORA;FGF1;TNF;FGF2;PLAU;NPPA;PIM

I1;C1S;C1R;SRC;GSTP1;WAS;RETN;TYMS;CST3;CDC42;MAPK8;TTR;MAPK1;CD59;NOS2;PKLI

H2;GSR;NR1H4;ADAM33;NR1H3;PTPN11;SOD2;MAPK14;SOD3;GCK;VEGFA;FAS;RAN;METAP

BP7;SAA1;FAS;RAMP1;RAB9B;CDK5R1;PF4;NCF1;CCNT1;DGKB;MIR26B;F13A1;PLAT;FGF1;NI

1;TNFRSF4;PDGFRB;MAP2K1;HGF;ADIPOQ;TAP1;ADAM10;CYBA;ADAM17;CD8A;MAPKAPK2

R;NOS3;CMA1;PLA2G2A;ADAM33;PTPN11;S100B;MAPK14;ATOX1;MAPK12;MAPK10;FABP5;IM

R3C1;FGF2;TNF;IGF1R;PPP1CC;FNTA;PDGFRB;MAP2K1;HGF;TAP1;ADAM10;CYBA;DGKZ;PGF

2;RAF1;PPIA;S100A9;S100A8;NOTCH3;CPB2;FECH;SOCS3;PSAP;ABL1;MBP;OSBPL9;HSPA8;TGI

;ADAM17;DGKQ;MAPKAPK2;PGR;RAF1;S100A9;CSNK1G2;S100A8;RTN4R;NOTCH3;CALCRL;I

[illegible]B1;EPHX2;DEFA1;SELE;NFKB1;IL2;IL4;ISG20;IL6;GDNF;MDM2;LCN2;MYD88;FGFR2;BCL2L1;

FECH;LPL;UAP1;RTN4;SOCS3;TBXA2R;PSAP;ABL1;OSBPL9;TGFB1;VDR;BDNF;EPHX2;HTR1A

;ESR1;NFKB1;IL2;ESR2;GFAP;IL6;BMP2;GDNF;RH
