# Supplementary material for: Mechanism of Radix Rhei Et Rhizome Intervention in Cerebral Infarction: A Research Based on Chemoinformatics and Systematic Pharmacology
Source: Evid Based Complement Alternat Med. 2021 Sep 6;2021:6789835. doi: 10.1155/2021/6789835 (PMC8440083; doi:10.1155/2021/6789835)
Supplement: Supplementary Materials — Table S1: potential targets for potential compounds; Table S2: proteomics data; Table S3: CI gene; Table S4: enrichment analysis of clusters based on gene ontology (GO) annotation of Radix Rhei Et Rhizome-CI PPI network; Table S5: pathway enrichment analysis of Radix Rhei Et Rhizome-CI PPI network; Table S6: reactome pathways of Radix Rhei Et Rhizome-CI PPI network; and Table S7: the biological processes, signaling pathways, and reactome of proteomics proteins' PPI network. [file 6789835.f1.zip › 6789835.f1/Table S7-1.pdf]

Table S7-1 The Biological Processes of Proteomics proteins' PPI Network

Term	Description	LogP	Enrichme	Z-score	Genes
GO:0099504	synaptic ves	-12	14	13	Calm1|Syp|Syt2|Syn1|Camk2a|Syt1|Bsn|Can
GO:0099003	vesicle-med	-11	13	12	Calm1|Syp|Syt2|Camk2a|Syt1|Canx|Snca|Pac
GO:0098657	import into	-11	6.6	10	Calm1|Syp|Syt2|Syt1|Clcn5|Canx|Snca|Aif1|
GO:0140238	presynaptic	-11	31	16	Calm1|Syp|Syt2|Syt1|Canx|Snca|Pacsin1|Cltc
GO:0048488	synaptic ves	-11	31	16	Calm1|Syp|Syt2|Syt1|Canx|Snca|Pacsin1|Cltc
GO:0036465	synaptic ves	-10	27	15	Calm1|Syp|Syt2|Syt1|Canx|Snca|Pacsin1|Cltc
GO:0006897	endocytosis	-9.5	6.8	9.3	Calm1|Syp|Syt2|Syt1|Clcn5|Canx|Snca|Aif1|
GO:0006836	neurotransm	-9.1	9.6	10	Calm1|Syp|Syt2|Syn1|Camk2a|Ywhaz|Syt1|S
GO:0007269	neurotransm	-9.1	13	11	Calm1|Syp|Syt2|Syn1|Camk2a|Syt1|Snca|Nsf
GO:0099643	signal releas	-9.1	13	11	Calm1|Syp|Syt2|Syn1|Camk2a|Syt1|Snca|Nsf
GO:0060627	regulation o	-8.7	6.5	8.8	Calm1|Ncam1|Syp|Syt2|Camk2a|Syt1|Snca|P
GO:0051640	organelle lo	-8.4	6.3	8.6	Calm1|Ncam1|Nefh|Syp|Syt2|Syn1|Camk2a|
GO:0001505	regulation o	-8	7.8	8.9	Calm1|Syp|Syt2|Syn1|Camk2a|Syt1|Snca|Aif
GO:0097479	synaptic ves	-7.9	12	10	Calm1|Syp|Syt2|Syn1|Camk2a|Syt1|Snca|Stx
GO:0061024	membrane c	-7.9	5.7	8	Calm1|Mbp|Syt2|Slc25a5|Cnp|Camk2a|Syt1|
GO:0098693	regulation o	-7.8	14	10	Calm1|Syp|Syt2|Syn1|Camk2a|Syt1|Bsn|Unc
GO:0023061	signal releas	-7.2	6.1	7.8	Calm1|Syp|Syt2|Syn1|Camk2a|Ywhaz|Syt1|S
GO:0031623	receptor int	-7.2	15	10	Snca|Pacsin1|Ppp3r1|Cltc|Nsf|Dnm1|Ubqln2|
GO:0033693	neurofilame	-7.2	2.40E+02	27	Nefh|Nefm|Nefl
GO:0006887	exocytosis	-7.1	7.3	8.2	Calm1|Ncam1|Syp|Syt2|Camk2a|Ywhaz|Syt1
GO:0097480	establishme	-7	11	9.3	Calm1|Syp|Syt2|Camk2a|Syt1|Snca|Stx7|Unc
GO:0048489	synaptic ves	-7	11	9.3	Calm1|Syp|Syt2|Camk2a|Syt1|Snca|Stx7|Unc
GO:0140029	exocytic pro	-6.9	18	11	Ncam1|Syt2|Camk2a|Ywhaz|Syt1|Snca|Unc1
GO:0043112	receptor me	-6.7	10	8.9	Snca|Pacsin1|Ppp3r1|Cltc|Nsf|Park7|Dnm1|U
GO:0007006	mitochondri	-6.6	16	10	Calm1|Slc25a5|Cnp|Camk2a|Snca|Hspa1b|C
GO:0048278	vesicle dock	-6.6	23	11	Ncam1|Camk2a|Syt1|Nsf|Stx7|Unc13a
GO:0016079	synaptic ves	-6.6	13	9.3	Calm1|Syp|Syt2|Camk2a|Syt1|Snca|Stx7|Unc
GO:1903937	response to	-6.6	1.80E+02	23	Nefh|Nefm|Nefl
GO:0050804	modulation	-6.5	5.3	7.1	Calm1|Ncam1|Syp|Syt2|Camk2a|Syt1|Snca|P
GO:0051648	vesicle loca	-6.5	8.3	8.1	Calm1|Syp|Syt2|Syn1|Camk2a|Syt1|Snca|Stx
GO:0099177	regulation o	-6.5	5.3	7.1	Calm1|Ncam1|Syp|Syt2|Camk2a|Syt1|Snca|P
GO:0006898	receptor-me	-6.3	9.3	8.2	Canx|Snca|Pacsin1|Ppp3r1|Cltc|Nsf|Dnm1|U
GO:0140056	organelle lo	-6.2	20	10	Ncam1|Camk2a|Syt1|Nsf|Stx7|Unc13a
GO:0048812	neuron proj	-6.2	5	6.8	Mbp|Ncam1|Nefh|Nefm|Syt2|Cnp|Camk2a|S
GO:0120039	plasma mem	-6.1	4.9	6.7	Mbp|Ncam1|Nefh|Nefm|Syt2|Cnp|Camk2a|S
GO:0051656	establishme	-6.1	5.8	7.1	Calm1|Nefh|Syp|Syt2|Camk2a|Ywhaz|Syt1|S
GO:0048858	cell projecti	-6	4.8	6.7	Mbp|Ncam1|Nefh|Nefm|Syt2|Cnp|Camk2a|S
GO:0017156	calcium ion	-5.9	10	8.2	Calm1|Syp|Syt2|Camk2a|Syt1|Snca|Stx7|Unc
GO:0032990	cell part mo	-5.9	4.7	6.5	Mbp|Ncam1|Nefh|Nefm|Syt2|Cnp|Camk2a|S
GO:0022406	membrane d	-5.9	17	9.6	Ncam1|Camk2a|Syt1|Nsf|Stx7|Unc13a
GO:0072347	response to	-5.9	13	8.7	Ncam1|Nefh|Hk1|Hspa5|Snca|Penk|Aif1
GO:0017157	regulation o	-5.8	8.1	7.6	Calm1|Ncam1|Syp|Syt2|Camk2a|Syt1|Snca|N
GO:0007568	aging	-5.8	5.5	6.8	Mbp|Mog|Ncam1|Cnp|Canx|Snca|Penk|Slc12
GO:0051650	establishme	-5.8	8	7.5	Calm1|Syp|Syt2|Camk2a|Syt1|Snca|Stx7|Unc
GO:0042063	gliogenesis	-5.7	6.8	7.1	Plp1|Cnp|Penk|Gap43|Ppp3r1|Dcx|Mapk1|Rh
GO:0045055	regulated ex	-5.7	7.8	7.4	Calm1|Syp|Syt2|Camk2a|Ywhaz|Syt1|Snca|S

GO:0045110	intermediate	-5.6	1.00E+02	17	Nefh|Nefm|Nefl
GO:0046928	regulation o	-5.6	11	8.2	Calm1|Syp|Syt2|Camk2a|Syt1|Snca|Unc13a
GO:0007005	mitochondri	-5.5	5.7	6.7	Calm1|Slc25a5|Cnp|Camk2a|Ywhaz|Snca|Pa
GO:0045109	intermediate	-5.4	37	12	Nefh|Nefm|Nefl|Krt2
GO:0030100	regulation o	-5.3	7.1	7	Calm1|Snca|Pacsin1|Ppp3r1|Nsf|Dcx|Dnm1|U
GO:0006119	oxidative ph	-5.2	13	8.3	Cox6a1|Snca|Rhoa|Park7|Dld|LOC679794
GO:2000300	regulation o	-5.1	13	8.1	Calm1|Syp|Syt2|Camk2a|Syt1|Unc13a
GO:0042775	mitochondri	-5.1	18	9.1	Cox6a1|Snca|Park7|Dld|LOC679794
GO:0001956	positive reg	-5.1	31	11	Syt2|Syt1|Snca|Unc13a
GO:0060052	neurofilame	-5.1	72	15	Nefh|Nefm|Nefl
GO:0009636	response to	-5.1	4.3	5.9	Calm1|Mbp|Nefh|Nefm|Hk1|Cnp|Penk|Nefl|M
GO:0098884	postsynaptic	-5	30	11	Pacsin1|Ppp3r1|Dnm1|Sirt2
GO:0140239	postsynaptic	-5	30	11	Pacsin1|Ppp3r1|Dnm1|Sirt2
GO:0034599	cellular resp	-5	6.6	6.6	Nefh|Nefm|Snca|Penk|Aif1|Mapk1|Park7|Mp
GO:0048678	response to	-5	12	8	Ncam1|Nefh|Nefm|Gap43|Aif1|Nefl
GO:1902803	regulation o	-5	12	7.9	Calm1|Syp|Syt2|Camk2a|Syt1|Unc13a
GO:0031103	axon regene	-5	17	8.8	Ncam1|Nefh|Nefm|Gap43|Nefl
GO:0042773	ATP synthe	-4.9	17	8.7	Cox6a1|Snca|Park7|Dld|LOC679794
GO:0046034	ATP metabo	-4.9	7.5	6.8	Hk1|Cox6a1|Snca|Rhoa|Park7|Hspa1b|Dld|L(
GO:0048284	organelle fu	-4.9	12	7.7	Syt2|Syt1|Snca|Nsf|Stx7|Chchd3
GO:0002090	regulation o	-4.9	17	8.6	Pacsin1|Ppp3r1|Nsf|Ubqln2|Sirt2
GO:0010001	glial cell dif	-4.9	7.4	6.7	Plp1|Cnp|Gap43|Ppp3r1|Mapk1|Rhoa|Wdr1|S
GO:0060359	response to	-4.9	8.9	7.1	Ncam1|Nefh|Hspa5|Snca|Penk|Aif1|Mapk1
GO:0051588	regulation o	-4.8	8.7	7	Calm1|Syp|Syt2|Camk2a|Syt1|Snca|Unc13a
GO:0072583	clathrin-dep	-4.7	25	9.7	Canx|Cltc|Dnm1|Ubqln2
GO:0015696	ammonium	-4.7	11	7.3	Syt2|Ywhaz|Syt1|Snca|Slc12a2|Park7
GO:0099590	neurotransm	-4.7	25	9.6	Pacsin1|Ppp3r1|Dnm1|Sirt2
GO:0031102	neuron proj	-4.7	15	8.1	Ncam1|Nefh|Nefm|Gap43|Nefl
GO:0010720	positive reg	-4.7	4.3	5.6	Syt2|Hspa5|Syt1|C1qbp|Pacsin1|Unc13a|Nefl
GO:0032386	regulation o	-4.7	5.2	5.9	Calm1|Nefh|Syp|Syt2|Camk2a|Syt1|Unc13a|N
GO:0009205	purine ribon	-4.7	6.8	6.4	Hk1|Cox6a1|Snca|Rhoa|Park7|Hspa1b|Dld|L(
GO:0045666	positive reg	-4.6	5.1	5.8	Syt2|Hspa5|Syt1|Pacsin1|Unc13a|Nefl|Dcx|C
GO:0009167	purine ribon	-4.6	6.7	6.3	Hk1|Cox6a1|Snca|Rhoa|Park7|Hspa1b|Dld|L(
GO:0009126	purine nucle	-4.6	6.7	6.3	Hk1|Cox6a1|Snca|Rhoa|Park7|Hspa1b|Dld|L(
GO:0009199	ribonucleosi	-4.6	6.7	6.3	Hk1|Cox6a1|Snca|Rhoa|Park7|Hspa1b|Dld|L(
GO:0009144	purine nucle	-4.6	6.6	6.2	Hk1|Cox6a1|Snca|Rhoa|Park7|Hspa1b|Dld|L(
GO:0097201	negative reg	-4.5	48	12	Bag3|Hspa1b|Sirt2
GO:0009117	nucleotide m	-4.5	4.5	5.5	Hk1|Cnp|Cox6a1|Snca|Dlat|Mapk1|Rhoa|Parl
GO:0009161	ribonucleosi	-4.5	6.4	6.1	Hk1|Cox6a1|Snca|Rhoa|Park7|Hspa1b|Dld|L(
GO:0006753	nucleoside p	-4.5	4.4	5.5	Hk1|Cnp|Cox6a1|Snca|Dlat|Mapk1|Rhoa|Parl
GO:0022904	respiratory e	-4.4	13	7.6	Cox6a1|Snca|Park7|Dld|LOC679794
GO:0006906	vesicle fusio	-4.4	13	7.6	Syt2|Syt1|Snca|Nsf|Stx7
GO:0017158	regulation o	-4.4	9.6	6.8	Calm1|Syp|Syt2|Camk2a|Syt1|Unc13a
GO:0009141	nucleoside t	-4.4	6.2	6	Hk1|Cox6a1|Snca|Rhoa|Park7|Hspa1b|Dld|L(
GO:0010975	regulation o	-4.4	4.3	5.4	Mbp|Nefm|Syt2|Hspa5|Syt1|Pacsin1|Unc13a|
GO:0010038	response to	-4.3	4.7	5.5	Calm1|Mbp|Ncam1|Syt2|Hspa5|Syt1|Snca|Pe
GO:0045104	intermediate	-4.3	20	8.5	Nefh|Nefm|Nefl|Krt2
GO:0021700	developmen	-4.3	6.1	5.9	Plp1|Ywhaz|Unc13a|Nefl|Rhoa|Dld|Sirt2|Cen

GO:0090174	organelle m	-4.3	13	7.3	Syt2|Syt1|Snca|Nsf|Stx7
GO:0099072	regulation o	-4.3	12	7.3	Camk2a|Pacsin1|Ppp3r1|Dnm1|Sirt2
GO:0009123	nucleoside r	-4.3	6	5.8	Hk1|Cox6a1|Snca|Rhoa|Park7|Hspa1b|Dld|L(
GO:0043269	regulation o	-4.2	3.9	5.2	Calm1|Syt2|Hk1|Camk2a|Syt1|Snca|Kcna6|R
GO:0022604	regulation o	-4.2	4.6	5.4	Mbp|Nefm|Syt2|Syt1|C1qbp|Unc13a|Nefl|Dc
GO:0051590	positive reg	-4.2	18	8.1	Syt2|Syt1|Snca|Unc13a
GO:0045333	cellular resp	-4.2	8.6	6.4	Cox6a1|Snca|Dlat|Park7|Dld|LOC679794
GO:0045103	intermediate	-4.1	18	8	Nefh|Nefm|Nefl|Krt2
GO:0007626	locomotory	-4.1	6.7	5.9	Cnp|Snca|Penk|Calb1|Park7|Dnm1|Cend1
GO:0008344	adult locom	-4.1	11	6.8	Cnp|Snca|Park7|Dnm1|Cend1
GO:0046902	regulation o	-4	17	7.8	Slc25a5|Cnp|Camk2a|Hspa1b
GO:0045664	regulation o	-4	3.6	4.9	Mbp|Nefm|Syt2|Hspa5|Syt1|Pacsin1|Unc13a|
GO:0009150	purine ribon	-4	4.8	5.3	Hk1|Cox6a1|Snca|Dlat|Rhoa|Park7|Hspa1b|D
GO:0055086	nucleobase-	-4	3.9	5	Hk1|Cnp|Cox6a1|Snca|Dlat|Mapk1|Rhoa|Parl
GO:0045744	negative reg	-4	16	7.5	Snca|Ywhab|Dnm1|Ubqln2
GO:0043279	response to	-3.9	7.9	6	Ncam1|Nefh|Hspa5|Snca|Penk|Aif1
GO:0048259	regulation o	-3.9	10	6.6	Pacsin1|Ppp3r1|Nsf|Ubqln2|Sirt2
GO:0006839	mitochondri	-3.9	7.8	6	Slc25a5|Cnp|Camk2a|Ywhaz|Bag3|Hspa1b
GO:0015872	dopamine tr	-3.9	16	7.5	Syt2|Syt1|Snca|Park7
GO:0016050	vesicle orga	-3.9	6.3	5.7	Syt2|Syt1|Snca|Nsf|Stx7|Unc13a|Dnm1
GO:0000226	microtubule	-3.9	4.2	5	Nefh|Nefm|Cnp|Snca|Cltc|Nefl|Dcx|Rhoa|Hsp
GO:0099149	regulation o	-3.9	30	9.2	Pacsin1|Ppp3r1|Sirt2
GO:0009259	ribonucleoti	-3.9	4.6	5.1	Hk1|Cox6a1|Snca|Dlat|Rhoa|Park7|Hspa1b|D
GO:0051668	localization	-3.9	7.6	5.9	Camk2a|Pacsin1|Ppp3r1|Stx7|Dnm1|Sirt2
GO:0010976	positive reg	-3.8	5.2	5.3	Syt2|Hspa5|Syt1|Pacsin1|Unc13a|Nefl|Dcx|C
GO:0006163	purine nucle	-3.8	4.5	5.1	Hk1|Cox6a1|Snca|Dlat|Rhoa|Park7|Hspa1b|D
GO:0050769	positive reg	-3.8	4.1	4.9	Syt2|Hspa5|Syt1|Pacsin1|Unc13a|Nefl|Dcx|C
GO:0019693	ribose phos	-3.8	4.5	5	Hk1|Cox6a1|Snca|Dlat|Rhoa|Park7|Hspa1b|D
GO:1903861	positive reg	-3.7	27	8.7	Syt2|Syt1|Unc13a
GO:0090559	regulation o	-3.7	14	6.9	Slc25a5|Cnp|Camk2a|Hspa1b
GO:0030900	forebrain de	-3.7	4.4	4.9	Ncam1|Nefh|Nefm|Cnp|Myo1d|Nrgn|Nefl|Dc
GO:0006914	autophagy	-3.7	4.9	5.1	Snca|Cltc|Pip4k2a|Park7|Bag3|Ubqln2|Sirt2|S
GO:0061919	process utili	-3.7	4.9	5.1	Snca|Cltc|Pip4k2a|Park7|Bag3|Ubqln2|Sirt2|S
GO:1903305	regulation o	-3.7	7	5.6	Calm1|Syp|Syt2|Camk2a|Syt1|Unc13a
GO:0008277	regulation o	-3.6	9.1	6	Syp|Snca|Ywhab|Dnm1|Ubqln2
GO:1903859	regulation o	-3.6	24	8.2	Syt2|Syt1|Unc13a
GO:0009410	response to	-3.6	4.8	5	Calm1|Ncam1|Nefh|Hk1|Hspa5|Snca|Penk|Ai
GO:0061025	membrane f	-3.6	8.7	5.9	Syt2|Syt1|Snca|Nsf|Stx7
GO:0061564	axon develo	-3.6	4.2	4.8	Mbp|Ncam1|Nefh|Nefm|Plp1|Cnp|Gap43|Nef
GO:0045927	positive reg	-3.6	5.5	5.1	Ncam1|Syt2|Syt1|Unc13a|Dcx|Mapk1|Rhoa
GO:0002237	response to	-3.6	4.2	4.8	Ncam1|Cnp|Snca|Penk|Mapk1|Rhoa|Park7|M
GO:0048639	positive reg	-3.5	6.7	5.4	Ncam1|Syt2|Syt1|Unc13a|Dcx|Mapk1
GO:0021510	spinal cord (	-3.5	8.6	5.8	Nefh|Nefm|Nefl|Dcx|Bag3
GO:0022900	electron trai	-3.5	8.6	5.8	Cox6a1|Snca|Park7|Dld|LOC679794
GO:0007017	microtubule	-3.5	3.5	4.5	Nefh|Nefm|Cnp|Bsn|Snca|Cltc|Nefl|Dcx|Rhoa
GO:0050806	positive reg	-3.5	5.5	5.1	Syt2|Syt1|Snca|Nrgn|Unc13a|Calb1|Mapk1
GO:0006979	response to	-3.5	4.2	4.7	Nefh|Nefm|Snca|Penk|Aif1|Mapk1|Park7|Mp
GO:0017144	drug metabo	-3.5	3.5	4.5	Hk1|Cox6a1|Snca|Aif1|Dlat|Rhoa|Park7|Hspa

GO:0048168	regulation o	-3.5	12	6.5	Syp|Camk2a|Snca|Unc13a
GO:0071616	acyl-CoA bi	-3.5	23	7.9	Snca|Dlat|Dld
GO:0035384	thioester bio	-3.5	23	7.9	Snca|Dlat|Dld
GO:1902074	response to	-3.5	23	7.9	Nefh|Hspa5|Nefl
GO:0072521	purine-conta	-3.5	4.1	4.7	Hk1|Cox6a1|Snca|Dlat|Rhoa|Park7|Hspa1b|D
GO:1905897	regulation o	-3.5	12	6.4	Hspa5|Park7|Hspa1b|Ubqln2
GO:0006091	generation c	-3.4	4.6	4.8	Hk1|Cox6a1|Snca|Dlat|Rhoa|Park7|Dld|LOC
GO:0042220	response to	-3.4	12	6.3	Ncam1|Nefh|Hspa5|Snca
GO:0021987	cerebral cor	-3.4	8.1	5.6	Nefh|Nefm|Nefl|Dcx|Rhoa
GO:0034620	cellular resp	-3.4	12	6.3	Hspa5|Canx|Bag3|Hspa1b
GO:0051962	positive regu	-3.4	3.6	4.5	Syt2|Hspa5|Syt1|Pacsin1|Unc13a|Nefl|Dcx|C
GO:0032410	negative reg	-3.4	11	6.2	Calm1|Snca|Rem2|Park7
GO:0048167	regulation o	-3.4	5.2	4.9	Syp|Camk2a|Snca|Nrgn|Unc13a|Calb1|Mapk
GO:0000904	cell morpho	-3.4	3.3	4.3	Mbp|Ncam1|Nefh|Nefm|Cnp|Camk2a|Gap43|
GO:0009896	positive regu	-3.4	4.4	4.7	Snca|Nsf|Pip4k2a|Bag3|Hspa1b|Ubqln2|Sirt2
GO:0015893	drug transpo	-3.3	6.1	5.1	Syt2|Slc25a5|Syt1|Snca|Slc6a9|Park7
GO:0051051	negative reg	-3.3	3.9	4.4	Calm1|Slc25a5|Snca|Pacsin1|Rem2|Park7|Ba
GO:0097435	supramolecu	-3.3	3.5	4.3	Nefh|Nefm|Snca|Aif1|Pacsin1|Nefl|Rhoa|Hsp
GO:0072595	maintenance	-3.2	19	7.1	Hk1|Hspa5|Park7
GO:0031503	protein-cont	-3.2	5.8	4.9	Camk2a|Pacsin1|Ppp3r1|Stx7|Dnm1|Sirt2
GO:0051937	catecholami	-3.2	10	5.8	Syt2|Syt1|Snca|Park7
GO:0072655	establishme	-3.2	10	5.8	Calm1|Hk1|Ywhaz|Bag3
GO:1902108	regulation o	-3.2	18	6.9	Slc25a5|Camk2a|Hspa1b
GO:0097484	dendrite ext	-3.2	18	6.9	Syt2|Syt1|Unc13a
GO:0015980	energy deriv	-3.2	5.7	4.8	Cox6a1|Snca|Dlat|Park7|Dld|LOC679794
GO:0007409	axonogenesi	-3.2	4.1	4.4	Mbp|Ncam1|Nefh|Nefm|Cnp|Gap43|Nefl|Dcx
GO:0071869	response to	-3.2	17	6.8	Penk|Mapk1|Sirt2
GO:0010506	regulation o	-3.2	5.6	4.8	Snca|Pip4k2a|Bag3|Ubqln2|Sirt2|Sptlc2
GO:0050808	synapse org	-3.1	4.1	4.4	Syn1|Ywhaz|Bsn|Snca|Gap43|Unc13a|Nefl|R
GO:0070585	protein loca	-3.1	9.7	5.6	Calm1|Hk1|Ywhaz|Bag3
GO:0033866	nucleoside b	-3.1	16	6.6	Snca|Dlat|Dld
GO:0034030	ribonucleosi	-3.1	16	6.6	Snca|Dlat|Dld
GO:0071867	response to	-3.1	16	6.6	Penk|Mapk1|Sirt2
GO:0034033	purine nucle	-3.1	16	6.6	Snca|Dlat|Dld
GO:0031346	positive regu	-3.1	4	4.3	Syt2|Hspa5|Syt1|Pacsin1|Unc13a|Nefl|Dcx|C
GO:0031331	positive regu	-3.1	4.6	4.5	Snca|Pip4k2a|Bag3|Hspa1b|Ubqln2|Sirt2|Sptl
GO:0006986	response to	-3.1	9.4	5.5	Hspa5|Canx|Bag3|Hspa1b
GO:0051592	response to	-3.1	6.7	5	Calm1|Syt2|Hspa5|Syt1|Penk
GO:0014059	regulation o	-3	16	6.5	Syt2|Syt1|Snca
GO:2001258	negative reg	-3	16	6.5	Calm1|Rem2|Park7
GO:1903573	negative reg	-3	16	6.5	Hspa5|Park7|Hspa1b
GO:0006904	vesicle dock	-3	16	6.5	Ncam1|Camk2a|Unc13a
GO:0014046	dopamine se	-3	16	6.5	Syt2|Syt1|Snca
GO:0035967	cellular resp	-3	9.3	5.5	Hspa5|Canx|Bag3|Hspa1b
GO:0072594	establishme	-3	4.5	4.4	Calm1|Hk1|Ywhaz|Hspa5|Ppp3r1|Mapk1|Bag
GO:0045185	maintenance	-3	9.2	5.4	Hk1|Hspa5|Ywhab|Park7
GO:0032768	regulation o	-3	15	6.4	Calm1|Snca|Park7
GO:0008038	neuron reco	-3	15	6.4	Ncam1|Ywhaz|Gap43

GO:0001504	neurotransm	-3	15	6.4	Snca|Slc6a9|Park7
GO:0007610	behavior	-3	3.2	4	Ncam1|Cnp|Ptgds|Snca|Penk|Nrgn|Calb1|Parl
GO:0010638	positive reg	-3	3.5	4.1	Syt2|Syt1|Mapk1|Pip4k2a|Rhoa|Hspa1b|Wdr
GO:0016241	regulation o	-3	9	5.4	Pip4k2a|Bag3|Ubqln2|Sptlc2
GO:0048667	cell morpho	-3	3.5	4.1	Mbp|Ncam1|Nefh|Nefm|Cnp|Camk2a|Gap43|
GO:0030534	adult behavi	-2.9	6.4	4.8	Cnp|Snca|Park7|Dnm1|Cend1
GO:0015844	monoamine	-2.9	8.8	5.3	Syt2|Syt1|Snca|Park7
GO:0030307	positive reg	-2.9	6.3	4.8	Syt2|Syt1|Unc13a|Dcx|Rhoa
GO:0072593	reactive oxy	-2.9	4.9	4.4	Snca|Aif1|Rhoa|Park7|Mpo|Sirt2
GO:0060291	long-term sy	-2.9	6.1	4.6	Snca|Nrgn|Unc13a|Calb1|Mapk1
GO:0033365	protein loca	-2.8	3.1	3.8	Calm1|Hk1|Camk2a|Ywhaz|Hspa5|Ppp3r1|M
GO:0043618	regulation o	-2.8	13	5.9	Bag3|Hspa1b|Sirt2
GO:0010823	negative reg	-2.8	13	5.9	Slc25a5|Bag3|Hspa1b
GO:0032434	regulation o	-2.8	8	5	Park7|Hspa1b|Ubqln2|Sirt2
GO:2001023	regulation o	-2.8	8	5	Syt2|Syt1|Snca|Park7
GO:0051353	positive reg	-2.8	13	5.8	Calm1|Snca|Park7
GO:0042743	hydrogen pe	-2.8	13	5.8	Snca|Park7|Mpo
GO:0021766	hippocampu	-2.8	7.8	4.9	Nefh|Nefm|Nefl|Dcx
GO:0048709	oligodendro	-2.8	7.8	4.9	Plp1|Cnp|Wdr1|Sirt2
GO:0007030	Golgi organ	-2.8	7.8	4.9	Ywhaz|Cltc|Mapk1|Surf4
GO:0018107	peptidyl-thr	-2.8	7.8	4.9	Hk1|Camk2a|Mapk1|Sirt2
GO:2000377	regulation o	-2.8	5.8	4.5	Snca|Aif1|Rhoa|Park7|Sirt2
GO:0043620	regulation o	-2.7	12	5.6	Bag3|Hspa1b|Sirt2
GO:0044283	small molec	-2.7	3.2	3.8	Plp1|Hk1|Cnp|Ptgds|Snca|Cltc|Park7|Mpo|Sp
GO:0035966	response to	-2.7	7.7	4.8	Hspa5|Canx|Bag3|Hspa1b
GO:0071241	cellular resp	-2.7	4.6	4.2	Ncam1|Syt2|Hspa5|Syt1|Snca|Mapk1
GO:1903409	reactive oxy	-2.7	7.5	4.8	Aif1|Rhoa|Park7|Mpo
GO:0018210	peptidyl-thr	-2.7	7.4	4.7	Hk1|Camk2a|Mapk1|Sirt2
GO:0002931	response to	-2.7	12	5.4	Hk1|Camk2a|Hspa1b
GO:0040008	regulation o	-2.6	3.1	3.7	Ncam1|Syt2|Syt1|Gap43|Unc13a|Dcx|Mapk1
GO:0072678	T cell migra	-2.6	11	5.3	Aif1|Slc12a2|Rhoa
GO:0032787	monocarbox	-2.6	3.4	3.7	Plp1|Hk1|Ptgds|Snca|Dlat|Park7|Echs1|Dld
GO:0016239	positive reg	-2.6	11	5.2	Pip4k2a|Bag3|Sptlc2
GO:0043388	positive reg	-2.6	11	5.2	Calm1|Park7|Sirt2
GO:0071216	cellular resp	-2.6	4.3	4	Ncam1|Hspa5|Mapk1|Rhoa|Park7|Sirt2
GO:0021537	telencephalo	-2.6	4.3	4	Nefh|Nefm|Nrgn|Nefl|Dcx|Rhoa
GO:0031329	regulation o	-2.6	3	3.6	Snca|Pip4k2a|Park7|Bag3|Hspa1b|Ubqln2|Sir
GO:0071407	cellular resp	-2.5	3	3.6	Nefh|Nefm|Hspa5|Penk|Aif1|Mapk1|Rhoa|Pa
GO:0021543	pallium dev	-2.5	5.2	4.1	Nefh|Nefm|Nefl|Dcx|Rhoa
GO:1902115	regulation o	-2.5	5.1	4.1	Gap43|Pip4k2a|Rhoa|Hspa1b|Ubqln2
GO:0051235	maintenance	-2.5	4.2	3.9	Calm1|Hk1|Hspa5|Snca|Ywhab|Park7
GO:0001819	positive reg	-2.5	3.7	3.7	Mbp|Hk1|Aif1|C1qbp|Park7|Hspa1b|Atp6ap2
GO:0034976	response to	-2.5	5.1	4.1	Hspa5|Canx|Park7|Hspa1b|Ubqln2
GO:0071248	cellular resp	-2.5	5.1	4.1	Syt2|Hspa5|Syt1|Snca|Mapk1
GO:0032886	regulation o	-2.5	5	4.1	Nefh|Snca|Cltc|Rhoa|Hspa1b
GO:2000378	negative reg	-2.5	10	5	Rhoa|Park7|Sirt2
GO:0032507	maintenance	-2.5	10	5	Hk1|Hspa5|Park7
GO:0021549	cerebellum	-2.5	6.5	4.3	Hspa5|Aif1|Cntn1|Cend1

GO:0045862	positive reg	-2.5	4.1	3.8	Mbp|Snca|Rhoa|Hspa1b|Ubqln2|Sirt2
GO:2000058	regulation o	-2.5	6.5	4.3	Park7|Hspa1b|Ubqln2|Sirt2
GO:0051781	positive reg	-2.5	9.9	4.9	Rhoa|Sirt2|Ybx1
GO:0016482	cytosolic tra	-2.4	6.4	4.3	Myo1d|Cltc|Mapk1|Surf4
GO:0034605	cellular resp	-2.4	9.8	4.9	Hspa5|Bag3|Hspa1b
GO:0050433	regulation o	-2.4	9.8	4.9	Syt2|Syt1|Snca
GO:0032413	negative reg	-2.4	9.6	4.8	Calm1|Rem2|Park7
GO:0016999	antibiotic m	-2.4	6.3	4.2	Snca|Dlat|Park7|Mpo
GO:0034765	regulation o	-2.4	3.5	3.6	Calm1|Hk1|Snca|Kcna6|Rem2|Park7|Wdr1
GO:0009060	aerobic resp	-2.4	9.5	4.8	Cox6a1|Dlat|LOC679794
GO:0021761	limbic syste	-2.4	6.2	4.2	Nefh|Nefm|Nefl|Dcx
GO:1900182	positive reg	-2.4	9.4	4.8	Mapk1|Park7|Bag3
GO:0051302	regulation o	-2.4	6.2	4.2	Calm1|Rhoa|Sirt2|Ybx1
GO:0010970	transport alo	-2.4	6.2	4.2	Nefh|Nefm|Bsn|Nefl
GO:0099111	microtubule	-2.4	6.1	4.2	Nefh|Nefm|Bsn|Nefl
GO:0050432	catecholami	-2.4	9.3	4.7	Syt2|Syt1|Snca
GO:0007422	peripheral n	-2.4	9.2	4.7	Nefh|Ppp3r1|Sirt2
GO:0022037	metencepha	-2.4	6	4.1	Hspa5|Aif1|Cntn1|Cend1
GO:1903532	positive reg	-2.4	3.4	3.5	Mbp|Syt2|Hk1|Syt1|Snca|Aif1|Unc13a
GO:0008088	axo-dendriti	-2.3	9	4.7	Nefm|Bsn|Nefl
GO:0051129	negative reg	-2.3	2.8	3.3	Mbp|Slc25a5|Snca|Pacsin1|Rhoa|Bag3|Hspa1
GO:0032496	response to	-2.3	3.4	3.5	Cnp|Snca|Penk|Mapk1|Rhoa|Park7|Mpo
GO:0042552	myelination	-2.3	5.9	4.1	Mbp|Plp1|Ppp3r1|Sirt2
GO:0032436	positive reg	-2.3	8.8	4.6	Hspa1b|Ubqln2|Sirt2
GO:0007272	ensheathme	-2.3	5.8	4	Mbp|Plp1|Ppp3r1|Sirt2
GO:0008366	axon enshea	-2.3	5.8	4	Mbp|Plp1|Ppp3r1|Sirt2
GO:0022898	regulation o	-2.3	4.5	3.7	Calm1|Hk1|Snca|Rem2|Park7
GO:0042176	regulation o	-2.3	3.8	3.5	Snca|Nsf|Park7|Hspa1b|Ubqln2|Sirt2
GO:0006637	acyl-CoA m	-2.3	8.5	4.5	Snca|Dlat|Dld
GO:0035383	thioester me	-2.3	8.5	4.5	Snca|Dlat|Dld
GO:0030705	cytoskeleton	-2.3	5.7	4	Nefh|Nefm|Bsn|Nefl
GO:0060560	developmen	-2.3	4.4	3.7	Syt2|Syt1|Unc13a|Slc12a2|Dcx
GO:0015672	monovalent	-2.2	3.2	3.3	Cox6a1|Clcn5|Nsf|Kcna6|Slc12a2|Cntn1|Parl
GO:0071277	cellular resp	-2.2	8.2	4.4	Syt2|Hspa5|Syt1
GO:0032409	regulation o	-2.2	4.3	3.6	Calm1|Hk1|Snca|Rem2|Park7
GO:0010256	endomembr	-2.2	3.7	3.4	Ywhaz|Pacsin1|Cltc|Mapk1|Dnm1|Surf4
GO:0044272	sulfur comp	-2.2	8.1	4.3	Snca|Dlat|Dld
GO:2000243	positive reg	-2.2	8	4.3	C1qbp|Park7|Sirt2
GO:0051258	protein poly	-2.2	4.3	3.6	Snca|Aif1|Nefl|Rhoa|Hspa1b
GO:0050803	regulation o	-2.2	4.3	3.6	Ywhaz|Snca|Gap43|Rhoa|Dnm1
GO:1904063	negative reg	-2.2	7.9	4.3	Calm1|Rem2|Park7
GO:0051924	regulation o	-2.2	4.2	3.6	Calm1|Camk2a|Snca|Rem2|Rhoa
GO:0048638	regulation o	-2.2	3.6	3.4	Ncam1|Syt2|Syt1|Unc13a|Dcx|Mapk1
GO:0031647	regulation o	-2.2	4.2	3.5	Snca|Mapk1|Park7|Bag3|Hspa1b
GO:0051341	regulation o	-2.2	7.9	4.3	Calm1|Snca|Park7
GO:0043271	negative reg	-2.2	5.4	3.8	Calm1|Snca|Rem2|Park7
GO:0061136	regulation o	-2.2	5.3	3.8	Park7|Hspa1b|Ubqln2|Sirt2
GO:0000045	autophagoso	-2.2	7.8	4.2	Pip4k2a|Bag3|Ubqln2

GO:0043524	negative reg	-2.2	5.3	3.8	Snca|Nefl|Rhoa|Park7
GO:0072659	protein loca	-2.2	4.2	3.5	Camk2a|Pacsin1|Cltc|Nsf|Stx7
GO:2000060	positive reg	-2.2	7.7	4.2	Hspa1b|Ubqln2|Sirt2
GO:2000241	regulation o	-2.1	5.2	3.7	Ptgds|C1qbp|Park7|Sirt2
GO:0032535	regulation o	-2.1	3.5	3.3	Nefm|Nefl|Slc12a2|Dcx|Rhoa|Wdr1
GO:1905037	autophagoso	-2.1	7.5	4.1	Pip4k2a|Bag3|Ubqln2
GO:0051047	positive reg	-2.1	3.1	3.2	Mbp|Syt2|Hk1|Syt1|Snca|Aif1|Unc13a
GO:0072676	lymphocyte	-2.1	7.4	4.1	Aif1|Slc12a2|Rhoa
GO:0018105	peptidyl-ser	-2.1	4	3.4	Hk1|Camk2a|Snca|Mapk1|Park7
GO:0072657	protein loca	-2.1	3	3.1	Calm1|Camk2a|Hspa5|Pacsin1|Cltc|Nsf|Stx7
GO:0051651	maintenance	-2	7	4	Hk1|Hspa5|Park7
GO:0071219	cellular resp	-2	3.9	3.3	Ncam1|Mapk1|Rhoa|Park7|Sirt2
GO:1990138	neuron proj	-2	4.9	3.5	Syt2|Syt1|Unc13a|Dcx
GO:0010959	regulation o	-2	3.3	3.2	Calm1|Camk2a|Snca|Rem2|Cntn1|Rhoa
GO:1900542	regulation o	-2	7	3.9	Snca|Rhoa|Park7
GO:0009108	coenzyme b	-2	4.9	3.5	Hk1|Snca|Dlat|Dld
GO:0051402	neuron apop	-2	3.9	3.3	Hspa5|Snca|Nefl|Rhoa|Park7
GO:0007215	glutamate re	-2	6.9	3.9	Camk2a|Unc13a|Park7
GO:0046677	response to	-2	2.9	3.1	Nefh|Hspa5|Penk|Nefl|Rhoa|Park7|Hspa1b
GO:0031099	regeneration	-2	3.9	3.3	Ncam1|Nefh|Nefm|Gap43|Nefl
GO:0001558	regulation o	-2	3.3	3.1	Syt2|Syt1|Unc13a|Dcx|Rhoa|Hspa1b
GO:0045921	positive reg	-2	6.8	3.9	Syt2|Syt1|Snca
GO:0016236	macroautop	-2	4.8	3.5	Pip4k2a|Bag3|Ubqln2|Sptlc2
GO:0045936	negative reg	-2	2.9	3	Myo1d|Snca|Ywhab|Ensa|Rhoa|Park7|Sirt2
GO:0010563	negative reg	-2	2.9	3	Myo1d|Snca|Ywhab|Ensa|Rhoa|Park7|Sirt2

Pacsin1|Ppp3r1|Cltc|Nsf|Slc12a2|Dcx|Slc6a9|Mapk1|Park7|Dnm1|Ubqln2|Sirt2
