# Supplementary material for: Mechanism of Radix Rhei Et Rhizome Intervention in Cerebral Infarction: A Research Based on Chemoinformatics and Systematic Pharmacology
Source: Evid Based Complement Alternat Med. 2021 Sep 6;2021:6789835. doi: 10.1155/2021/6789835 (PMC8440083; doi:10.1155/2021/6789835)
Supplement: Supplementary Materials — Table S1: potential targets for potential compounds; Table S2: proteomics data; Table S3: CI gene; Table S4: enrichment analysis of clusters based on gene ontology (GO) annotation of Radix Rhei Et Rhizome-CI PPI network; Table S5: pathway enrichment analysis of Radix Rhei Et Rhizome-CI PPI network; Table S6: reactome pathways of Radix Rhei Et Rhizome-CI PPI network; and Table S7: the biological processes, signaling pathways, and reactome of proteomics proteins' PPI network. [file 6789835.f1.zip › 6789835.f1/Table S7-2.pdf]

Table S7-2 The Signaling Pathways of Proteomics proteins' PPI Network

Term	Description	LogP	Enrichme	Z-score	Genes
rno04721	Synaptic vesicle cycle	-5.2	19	9.4	Syt1|Cltc|Nsf|Unc13a|Dnm1
rno05014	Amyotrophic lateral sclero:	-4.1	17	7.8	Nefh|Nefm|Ppp3r1|Nefl
rno05010	Alzheimer's disease	-3.9	7.9	6	Calm1|Cox6a1|Snca|Ppp3r1|Mapl
rno04720	Long-term potentiation	-3.8	15	7.1	Calm1|Camk2a|Ppp3r1|Mapk1
rno05020	Prion diseases	-3.4	21	7.6	Ncam1|Hspa5|Mapk1
rno05012	Parkinson's disease	-3.4	7.9	5.5	Slc25a5|Cox6a1|Snca|Park7|Nduf
rno04921	Oxytocin signaling pathwa	-3.3	7.8	5.5	Calm1|Camk2a|Ppp3r1|Mapk1|Rh
rno04022	cGMP-PKG signaling path	-3.2	7.2	5.2	Calm1|Slc25a5|Ppp3r1|Mapk1|Rh
rno04961	Endocrine and other factor	-2.9	14	6.2	Cltc|Calb1|Dnm1
rno01200	Carbon metabolism	-2.8	7.8	4.9	Hk1|Dlat|Echs1|Dld
rno04722	Neurotrophin signaling pat	-2.7	7.7	4.9	Calm1|Camk2a|Mapk1|Rhoa
rno05214	Glioma	-2.6	11	5.3	Calm1|Camk2a|Mapk1
rno05031	Amphetamine addiction	-2.6	11	5.2	Calm1|Camk2a|Ppp3r1
rno00010	Glycolysis / Gluconeogene	-2.5	10	5	Hk1|Dlat|Dld
rno05133	Pertussis	-2.4	9.8	4.9	Calm1|Mapk1|Rhoa
rno05100	Bacterial invasion of epithe	-2.4	9.2	4.7	Cltc|Rhoa|Dnm1
rno04141	Protein processing in endo|	-2.3	5.8	4	Hspa5|Canx|Hspa1b|Ubqln2
rno04360	Axon guidance	-2.2	5.4	3.8	Camk2a|Ppp3r1|Mapk1|Rhoa
rno04912	GnRH signaling pathway	-2.2	7.8	4.2	Calm1|Camk2a|Mapk1
rno04915	Estrogen signaling pathway	-2.1	7.6	4.2	Calm1|Mapk1|Hspa1b
rno04020	Calcium signaling pathway	-2.1	5.1	3.7	Calm1|Slc25a5|Camk2a|Ppp3r1
rno04713	Circadian entrainment	-2.1	7.3	4.1	Calm1|Camk2a|Mapk1
rno04916	Melanogenesis	-2.1	7.2	4	Calm1|Camk2a|Mapk1
rno04612	Antigen processing and pre	-2.1	7.2	4	Hspa5|Canx|Hspa1b
rno04024	cAMP signaling pathway	-2	4.9	3.6	Calm1|Camk2a|Mapk1|Rhoa
rno04922	Glucagon signaling pathwa	-2	7	4	Calm1|Camk2a|Ppp3r1
rno04145	Phagosome	-2	4.8	3.5	Canx|Stx7|Tubb4b|Mpo
rno05016	Huntington's disease	-2	4.8	3.5	Slc25a5|Cox6a1|Cltc|Ndufb10

x1|Ndufb10
