# Supplementary material for: Mechanism of Radix Rhei Et Rhizome Intervention in Cerebral Infarction: A Research Based on Chemoinformatics and Systematic Pharmacology
Source: Evid Based Complement Alternat Med. 2021 Sep 6;2021:6789835. doi: 10.1155/2021/6789835 (PMC8440083; doi:10.1155/2021/6789835)
Supplement: Supplementary Materials — Table S1: potential targets for potential compounds; Table S2: proteomics data; Table S3: CI gene; Table S4: enrichment analysis of clusters based on gene ontology (GO) annotation of Radix Rhei Et Rhizome-CI PPI network; Table S5: pathway enrichment analysis of Radix Rhei Et Rhizome-CI PPI network; Table S6: reactome pathways of Radix Rhei Et Rhizome-CI PPI network; and Table S7: the biological processes, signaling pathways, and reactome of proteomics proteins' PPI network. [file 6789835.f1.zip › 6789835.f1/Table S7-3.pdf]

**Table S7-3 The Reactome Pathways of Proteomics proteins' PPI Network**

| Term | Descriptio | LogP | Enrichme | Z-score | Genes |
|---|---|---|---|---|---|
| R-RNO-37 | L1CAM in | -6.9 | 25 | 12 | Ncam1\|Gap43\|Cltc\|Mapk1\|Dnm1\|Tubb4b |
| R-RNO-38 | Beta-caten | -6.4 | 21 | 11 | Calm1\|Camk2a\|Ppp3r1\|Cltc\|Rhoa\|Rps27a |
| R-RNO-88 | Clathrin-m | -6.1 | 14 | 9.1 | Syt2\|Syt1\|Pacsin1\|Cltc\|Dnm1\|Ubqln2\|Rps27a |
| R-RNO-11 | Transmissi | -6 | 10 | 8.3 | Calm1\|Syn1\|Camk2a\|Syt1\|Epb41l1\|Nsf\|Nrgn |
| R-RNO-56 | RAF/MAP | -5.2 | 10 | 7.6 | Calm1\|Ncam1\|Camk2a\|Ywhab\|Nefl\|Mapk1\|R |
| R-RNO-56 | MAPK1/M | -5.2 | 9.9 | 7.5 | Calm1\|Ncam1\|Camk2a\|Ywhab\|Nefl\|Mapk1\|R |
| R-RNO-44 | Signaling b | -5.1 | 6.7 | 6.7 | Calm1\|Ncam1\|Camk2a\|Ywhaz\|Canx\|Ywhab\| |
| R-RNO-45 | Regulation | -5.1 | 31 | 11 | Ywhaz\|Ywhab\|Hspa1b\|Rps27a |
| R-RNO-96 | FLT3 Sign | -5 | 9.4 | 7.3 | Calm1\|Ncam1\|Camk2a\|Ywhab\|Nefl\|Mapk1\|R |
| R-RNO-44 | Activation | -4.9 | 28 | 10 | Calm1\|Camk2a\|Nrgn\|Nefl |
| R-RNO-43 | Recycling | -4.9 | 28 | 10 | Cltc\|Mapk1\|Dnm1\|Tubb4b |
| R-RNO-11 | Activation | -4.8 | 60 | 13 | Ywhaz\|Ppp3r1\|Ywhab |
| R-RNO-44 | Other inter | -4.8 | 8.8 | 7 | Calm1\|Ncam1\|Camk2a\|Ywhab\|Nefl\|Mapk1\|R |
| R-RNO-56 | MAPK fan | -4.8 | 8.8 | 7 | Calm1\|Ncam1\|Camk2a\|Ywhab\|Nefl\|Mapk1\|R |
| R-RNO-11 | Neurotrans | -4.8 | 11 | 7.5 | Calm1\|Camk2a\|Epb41l1\|Nsf\|Nrgn\|Nefl |
| R-RNO-19 | Signaling b | -4.7 | 8.4 | 6.8 | Calm1\|Camk2a\|Ywhaz\|Ppp3r1\|Cltc\|Rhoa\|Rps |
| R-RNO-42 | Axon guid | -4.6 | 8.2 | 6.7 | Ncam1\|Gap43\|Cltc\|Mapk1\|Rhoa\|Dnm1\|Tubb |
| R-RNO-11 | Neuronal S | -4.5 | 6.5 | 6.2 | Calm1\|Syn1\|Camk2a\|Syt1\|Epb41l1\|Nsf\|Nrgn |
| R-RNO-88 | Cargo reco | -4.4 | 13 | 7.6 | Syt2\|Syt1\|Cltc\|Ubqln2\|Rps27a |
| R-RNO-12 | Cytokine S | -4.4 | 5.4 | 5.7 | Calm1\|Ncam1\|Camk2a\|Ywhaz\|Canx\|Ywhab\| |
| R-RNO-12 | Adaptive I | -4.3 | 4.7 | 5.5 | Calm1\|Ywhaz\|Hspa5\|Canx\|Ppp3r1\|Cltc\|Ywha |
| R-RNO-11 | Activation | -4.3 | 40 | 11 | Ywhaz\|Ppp3r1\|Ywhab |
| R-RNO-43 | Unblocking | -4.1 | 36 | 10 | Calm1\|Camk2a\|Nefl |
| R-RNO-39 | Glutamate | -3.9 | 31 | 9.4 | Camk2a\|Epb41l1\|Nsf |
| R-RNO-39 | Trafficking | -3.9 | 31 | 9.4 | Camk2a\|Epb41l1\|Nsf |
| R-RNO-15 | Signaling b | -3.9 | 30 | 9.2 | Ywhaz\|Ybx1\|Rps27a |
| R-RNO-22 | Cellular re | -3.9 | 6.2 | 5.6 | Camk2a\|Hspa5\|Mapk1\|Bag3\|Hspa1b\|Tubb4b |
| R-RNO-12 | Developme | -3.8 | 5.2 | 5.3 | Ncam1\|Gap43\|Cltc\|Mapk1\|Rhoa\|Dnm1\|Tubb |
| R-RNO-11 | Downstrea | -3.8 | 29 | 9 | Calm1\|Ppp3r1\|Rps27a |
| R-RNO-19 | Membrane | -3.8 | 4.6 | 5.1 | Syt2\|Syt1\|Pacsin1\|Cltc\|Nsf\|Dnm1\|Tubb4b\|Ub |
| R-RNO-56 | Negative r | -3.8 | 28 | 8.8 | Ywhab\|Mapk1\|Rps27a |
| R-RNO-33 | Cellular re | -3.7 | 14 | 6.9 | Camk2a\|Hspa5\|Bag3\|Hspa1b |
| R-RNO-56 | Vesicle-me | -3.7 | 4.4 | 4.9 | Syt2\|Syt1\|Pacsin1\|Cltc\|Nsf\|Dnm1\|Tubb4b\|Ub |
| R-RNO-56 | RAF activa | -3.6 | 24 | 8.2 | Calm1\|Camk2a\|Ywhab |
| R-RNO-40 | PCP/CE pa | -3.5 | 22 | 7.8 | Cltc\|Rhoa\|Rps27a |
| R-RNO-56 | CLEC7A ( | -3.4 | 20 | 7.4 | Calm1\|Ppp3r1\|Rps27a |
| R-RNO-19 | RHO GTP | -3.3 | 6 | 5 | Calm1\|Ywhaz\|Ywhab\|Mapk1\|Rhoa\|Tubb4b |
| R-RNO-10 | Intrinsic Pa | -3.3 | 19 | 7.2 | Ywhaz\|Ppp3r1\|Ywhab |
| R-RNO-89 | Cellular re | -3.2 | 4.9 | 4.7 | Camk2a\|Hspa5\|Mapk1\|Bag3\|Hspa1b\|Tubb4b |
| R-RNO-53 | Programme | -3.2 | 10 | 5.8 | Ywhaz\|Ppp3r1\|Ywhab\|Rps27a |
| R-RNO-19 | Gap juncti | -3.2 | 17 | 6.8 | Cltc\|Dnm1\|Tubb4b |
| R-RNO-40 | Ca2+ path | -3.2 | 17 | 6.8 | Calm1\|Camk2a\|Ppp3r1 |
| R-RNO-21 | MHC class | -3.2 | 9.9 | 5.7 | Canx\|Cltc\|Dnm1\|Tubb4b |
| R-RNO-15 | Gap juncti | -3.1 | 17 | 6.7 | Cltc\|Dnm1\|Tubb4b |
| R-RNO-18 | Signaling b | -3.1 | 16 | 6.5 | Cltc\|Mapk1\|Rhoa |
| R-RNO-98 | Signaling b | -3 | 16 | 6.5 | Calm1\|Ppp3r1\|Rps27a |

| | | | | | |
|---|---|---|---|---|---|
| R-RNO-33 | Regulation | -2.9 | 14 | 6 | Hspa5\|Bag3\|Hspa1b |
| R-RNO-56 | C-type lect | -2.9 | 14 | 6 | Calm1\|Ppp3r1\|Rps27a |
| R-RNO-56 | RHO GTP | -2.8 | 13 | 5.7 | Ywhaz\|Ywhab\|Rhoa |
| R-RNO-16 | Signaling l | -2.7 | 12 | 5.6 | Cltc\|Mapk1\|Rhoa |
| R-RNO-76 | Platelet act | -2.7 | 5.6 | 4.4 | Calm1\|Ywhaz\|Mapk1\|Rhoa\|Wdr1 |
| R-RNO-10 | Hemostasis | -2.7 | 3.9 | 3.9 | Calm1\|Ywhaz\|C1qbp\|Mapk1\|Rhoa\|Tubb4b\|W |
| R-RNO-24 | Fc epsilon | -2.6 | 11 | 5.3 | Calm1\|Ppp3r1\|Rps27a |
| R-RNO-19 | Signaling l | -2.6 | 4.4 | 4 | Calm1\|Ywhaz\|Ywhab\|Mapk1\|Rhoa\|Tubb4b |
| R-RNO-16 | Innate Imn | -2.6 | 3 | 3.6 | Calm1\|Ppp3r1\|Mapk1\|Rhoa\|Hspa1b\|Tubb4b\| |
| R-RNO-98 | Ion channe | -2.6 | 6.8 | 4.5 | Calm1\|Camk2a\|Clcn5\|Rps27a |
| R-RNO-26 | Stimuli-ser | -2.3 | 8.7 | 4.5 | Calm1\|Clcn5\|Rps27a |
| R-RNO-10 | Apoptosis | -2.2 | 8.2 | 4.4 | Ywhaz\|Ppp3r1\|Ywhab |
| R-RNO-90 | Signaling l | -2.2 | 5.5 | 3.9 | Calm1\|Dlat\|Mapk1\|Dld |
| R-RNO-38 | Transport | -2.2 | 3.2 | 3.3 | Calm1\|Slc25a5\|Camk2a\|Clcn5\|Cltc\|Slc6a9\|R |
| R-RNO-69 | G2/M Che | -2.1 | 7.6 | 4.2 | Ywhaz\|Ywhab\|Rps27a |
| R-RNO-67 | Neutrophil | -2.1 | 3.4 | 3.2 | Mapk1\|Rhoa\|Hspa1b\|Tubb4b\|Atp6ap2\|Mpo |

ab|Dnm1|Tubb4b|Rps27a
